# A quantitative epigenetic approach for the assessment of cigarette consumption

**DOI:** 10.3389/fpsyg.2015.00656

**Published:** 2015-06-02

**Authors:** Robert Philibert, Nancy Hollenbeck, Eleanor Andersen, Terry Osborn, Meg Gerrard, Frederick X. Gibbons, Kai Wang

**Affiliations:** ^1^Department of Psychiatry, University of Iowa, Iowa City, IAUSA; ^2^Behavioral Diagnostics, Iowa City, IAUSA; ^3^Department of Psychology–Center for Health Intervention and Prevention, University of Connecticut, Storrs, CTUSA; ^4^Department of Biostatistics, College of Public Health, University of Iowa, Iowa City, IAUSA

**Keywords:** DNA methylation, epigenetics, aryl hydrocarbon receptor repressor, cg05575921, diagnostics, smoking, e-cigarettes

## Abstract

Smoking is the largest preventable cause of morbidity and mortality in the world. Despite the development of numerous preventive and treatment interventions, the rate of daily smoking in the United States is still approximately 22%. Effective psychosocial interventions and pharmacologic agents exist for the prevention and treatment of smoking. Unfortunately, both approaches are hindered by our inability to accurately quantify amount of cigarette consumption from the point of initial experimentation to the point of total dependency. Recently, we and others have demonstrated that smoking is associated with genome-wide changes in DNA methylation. However, whether this advance in basic science can be employed as a reliable assay that is useful for clinical diagnosis and treatment has not been shown. In this communication, we determine the sensitivity and specificity of five of the most consistently replicated CpG loci with respect to smoking status using data from a publically available dataset. We show that methylation status at a CpG locus in the aryl hydrocarbon receptor repressor, cg05575921, is both sensitive and specific for smoking status in adults with a receiver operated curve characteristic area under the curve of 0.99. Given recent demonstrations that methylation at this locus reflects both intensity of smoking and the degree of smoking cessation, we conclude that a methylation-based diagnostic at this locus could have a prominent role in understanding the impact of new products, such as e-cigarettes on initiation of cigarette smoking among adolescents, while improving the prevention and treatment of smoking, and smoking related disorders.

## Introduction

Smoking is the largest preventable cause of morbidity and mortality in the United States. Each year, nearly 1/2 million Americans die secondary to the effects of smoking (Centers for Disease Control and Prevention, 2008). Beyond the personal toll, smoking has an enormous financial impact on the United States. Each year, the U.S. spends nearly $100 billion on the treatment of smoking-related illnesses and suffers an additional $100 billion in lost wages (Centers for Disease Control and Prevention, 2008).

In response to this public health crisis, state and federal governments have implemented a series of policy measures and supported the implementation of preventive interventions by public health workers. In addition, large Pharma has collaborated with academia to develop effective medications, such as bupropion and varenicline for smoking cessation (Mills et al., 2012). Despite these efforts, 22% of all U.S. adults reported daily smoking in 2010 (Centers for Disease Control and Prevention, 2011).

Surprisingly, one of the largest barriers to developing more effective smoking prevention and cessation interventions has been our relative inability to objectively quantify tobacco consumption. Currently, there are three principal methods for determining tobacco consumption. The first is self-report. In general population samples, self-report is an adequate measure of tobacco consumption. However, in high risk populations and in adolescents, self-report is often unreliable (Fendrich et al., 2005; Jarvis et al., 2008; Gorber et al., 2009). This is especially true in higher risk clinical settings, such as pregnancy, where patients are sometimes reluctant to confide to physicians their inability to quit (Shipton et al., 2009; Dietz et al., 2011).

In attempts to supplement self-report, objective measures of tobacco consumption, such as serum or salivary cotinine or exhaled carbon monoxide levels, are sometimes used. Unfortunately, each of these approaches for determining smoking status has its limitations (Florescu et al., 2009). While easy to perform, exhaled carbon monoxide levels are only useful for detecting smoking within 3–4 h of the last cigarette (Jatlow et al., 2008; Florescu et al., 2009). Serum and salivary cotinine levels are more sensitive, generally detecting use with 48 hours, but are usually determined using more difficult to perform enzyme linked immunoassays (ELISA; Jatlow et al., 2008). These relatively narrow time windows for detection limit the usefulness of these approaches in detecting nascent smoking among adolescents during the critical smoking initiation period, or for “chippers,” i.e., light and intermittent non-daily smokers that use cigarettes only in specific situations such as bars or with their first cup of coffee in the morning (Levinson et al., 2007).

Over the past several years, the limitations of cotinine based assays of smoking have made more apparent by the introduction of e-cigarettes. These devices, which vaporize a solution of propylene glycol that contains nicotine, are gaining popularity use among adolescents, with prevalence data showing that use at least doubled in the U.S. and Britain every year from 1% in 2009 to 2% in 2010, and 6–7% in 2012 (Pepper et al., 2013; Centers for Disease Control and Prevention, 2014). Although perceived by teens as being healthier than cigarettes, many e-cigarette users also smoke cigarettes, and there is considerable concern from public health experts that these devices will further increase teen smoking (Grana et al., 2014; Wills et al., 2015). Since use of these e-cigarettes, nicotine replacement agents, such as the “patch,” and non-smoked forms of tobacco consumption also results in positive serum and salivary cotinine results, the usefulness of cotinine determinations in differentiating between their use and surreptitious cigarette smoking and guiding smoking cessation treatment is relatively limited. Hence, there is urgent need for new measures for the detection of cigarette consumption.

Recently developed epigenetic approaches to determine smoking status may provide the necessary tools to bridge the chasm in our ability to detect and quantitate cigarette consumption. Beginning in the first decade of this millennium, we and others demonstrated gene specific changes in DNA methylation in response to smoking (Philibert et al., 2008; Breton et al., 2009; Launay et al., 2009). When the first truly genome-wide platform for measuring smoking consumption was developed (Illumina HumanMethylation450 BeadChip), we used it to show that demethylation at a CpG residue interrogated by probe cg05575921 in the aryl hydrocarbon receptor is a sensitive and highly specific indicator of cigarette consumption (Monick et al., 2012). Since that time, numerous independent studies using this chip have confirmed these findings in DNA from newborns, adolescents, and adults (see **Table 1**; Joubert et al., 2012; Philibert et al., 2012, 2013; Shenker et al., 2012; Zeilinger et al., 2013; Besingi and Johansson, 2014; Dogan et al., 2014; Elliott et al., 2014; Harlid et al., 2014; Tsaprouni et al., 2014; Guida et al., 2015). In addition, three groups have shown that smoking induced methylation changes can revert as a function of smoking cessation and that cg05575921 is the most sensitive residue in the genome in response to smoking cessation (Zeilinger et al., 2013; Harlid et al., 2014; Guida et al., 2015). Finally, we have shown that the effects of smoking on DNA methylation are unique to smoking and are not affected by alcohol consumption, thus allowing smoking and alcohol consumption status to be assessed simultaneously from the same dataset (Philibert et al., 2014a). Taken together, these studies indicate that DNA methylation assessments hold considerable promise as a tool for supplementing self-report information in smoking prevention and smoking cessation efforts. The question is as to how they will be integrated into our current prevention and treatment framework.

For now, the studies listed in **Table 1** indicate a potential for DNA methylation to be used as an independent method to unequivocally establish the presence of smoking. This capability may be potentially useful under certain circumstances. For example, it is well established that smoking is a modifiable risk factor for certain high risk medical procedures with many physicians refusing to operate unless the patient has quit smoking (Peters et al., 2004). Or, in efforts to promote a healthier workforce, prominent governmental bodies such as the World Health Organization (WHO) as well as many private employers are refusing to employ smokers (Cage, 2005). By taking advantage of the inherent stability of methylation signatures over short periods of time, the potential surety of detection afforded by these methylation technologies provides a framework around which the appropriate incentives can be placed to improve medical outcomes and decrease overall healthcare costs.

**Table 1:** Results of replication attempts of the original findings by Monick et al. (2012) with respect to methylation status at AHRR probe cg05575921 using independent populations.

Reference	Rank (of 485557 probes)	FDR *P*-values	Population
Philibert et al. (2012)	1st	3 × 10^-7^	Adolescents
Joubert et al. (2012)	1st	8 × 10^-33^	Newborns
Shenker et al. (2012)	1st	2 × 10^-15^	Adults
Philibert et al. (2013)	1st	2 × 10^-3^	Young adults
Zeilinger et al. (2013)	1st	3 × 10^-182^	Adults
Dogan et al. (2014)	2nd	6 × 10^-19^	Adults
Besingi and Johansson (2014)	1st	7 × 10^-70^	Adults
Elliott et al. (2014)	1st	6 × 10^-59^	Adults
Tsaprouni et al. (2014)	1st	9 × 10^-69^	Adults
Harlid et al. (2014)	2nd	2 × 10^-2^	Adults
Guida et al. (2015)	1st	1 × 10^-106^	Adults

However, in order for that vision be realized, the current genome wide approaches need to be reduced to a potentially clinical format. In hopes of accomplishing this, using the information generated in these studies, our academic/corporate consortium devised an easy to use quantitative PCR assay of cg05575921 methylation status referred to as Smoke Signature (Dogan et al., 2014). Nevertheless, the question remains as to whether determination of methylation at this locus or any other of the loci that were commonly identified in the prior studies are solely capable of determining smoking status.

As a first step in answering this question, in this study, we use publically available methylation data from a recently completed study and standard analytic approaches to test single and multiple locus approaches to the determination of smoking consumption.

## Materials and Methods

The data used in the study are derived from subjects who participated in a previously described National Institutes of Health study that examined the effects of alcohol on DNA methylation (R43AA022041; Philibert et al., 2014a). All protocols and procedures used in this study were approved by the University of Iowa Institutional Review Board.

In brief, the drinking participants (drinkers) were recruited from either local alcohol treatment centers or the University of Iowa Hospitals and Clinics for the treatment of alcohol dependence. Participants were approached after they had detoxified from alcohol intake (between 3–7 days after the last drink). The inclusion criteria for the study specified good overall health and the absence of active substance use outside of alcohol or tobacco. Furthermore, participants could not be taking any medication hypothesized to affect DNA methylation (such as valproic acid). The controls (non-drinkers) were recruited from the University of Iowa community and were required to be abstinent from alcohol and all other forms of substance use with the exception of tobacco. All participants reported the number of cigarettes smoked per day over the past month and past year.

After consent was obtained, all participants were interviewed with a modified version of the Semi Structured Assessment for the Genetics of Alcoholism, Version 2 (SSAGA-II) by a trained research assistant (Bucholz et al., 1994). The SSAGA-II is a publically available standardized interview that demographic and modules for each of the major behavioral disorders with particular emphasis on the substance use disorders (see Appendix 1). This information was supplemented by a questionnaire that assessed consumption of substances over the past day, past week, past month, past 6 months, and past year (see Appendix 2). They were then phlebotomized to provide the biomaterial for the current study. Serum samples were obtained using standard serum separator tubes and stored at -80°C until analyzed. Mononuclear cell pellets were obtained via gradient centrifugation of whole blood through Ficoll as previously described (Philibert et al., 2012). DNA was then prepared from these samples using a QiaAMP DNA (Qiagen, Germany) according to the manufacturer’s instructions.

We defined smokers as those individuals who reported the recent use of cigarettes or other forms of combustible tobacco while we defined those who did not use any type of combusted tobacco or cannabis as non-smokers. In order to confirm self-reported smoking status, serum cotinine and hydroxy-tetrahydro-cannabinol (hydroxy-THC) levels were assessed using immuno ELISA supplied by Abnova (Taiwan) according to manufacturer’s directions. Data from one participant whose serum assessments were not consistent with self-report were excluded from further analysis in the study. Because the serum cotinine levels are highly dependent as to the time of the last cigarette and the two of the facilities where we recruited subjects did not allow free access to cigarettes at all time, we used serum cotinine levels as only as an indicator of smoking status and not as an indicator of total cigarette consumption.

The methylation data for the five loci described in the current study were extracted from the previously conducted genome-wide methylation assessments which are publicly available (GEO accession number GSE57853). These DNA methylation assessments were conducted by the University of Minnesota Genome Center using the Illumina HumanMethylation450 BeadChip (Illumina, San Diego, CA, USA; Philibert et al., 2012, 2013). The resulting data were inspected for complete bisulfite conversion. Then average β-values (the ratio of the methylated probe fluorescence intensity to the sum of the methylated and unmethylated probe fluorescence intensities) were determined using the GenomeStudio^®^ suite of programs. These values were then cleaned using a Perl-based algorithm to remove unreliable data points before deposition into the Gene Expression Omnibus (GEO) website (Dogan et al., 2014).

Clinical and demographic data were then analyzed using JMP version 10 (SAS Institute, Cary, NC, USA, software company) using the tests indicated in the text. The Receiver Operator Characteristic analyses were also conducted using this package.

## Results

In the previous study of the effects of alcohol consumption on DNA methylation, we used data from a total of 66 participants. For the purposes of the current study, we excluded the data from five of those participants. The first was excluded because his substance use self-report of abstinence was not consistent with our serum ELISA assessments. The second and third were excluded because while they were not current smokers, they were both cigarette smokers in the past 10 years and were currently smoking cannabis-which is commonly mixed with tobacco to improve pyrolysis. The fourth and fifth were excluded from the primary analyses because they used chew or snuff which precluded serum verification of smoke free status.

The demographic characteristics of the remaining 61 participants whose data are included in the main analyses are given in **Table 2**. Overall, the middle-aged participants were mostly male and white. Only two of the smokers did not have a history of recent alcohol consumption. All of the participants who reported daily smoking had detectable levels of cotinine in their serum (average 99 ± 42 ng/ml). Please note that because all of the drinkers were ascertained in smoke-free facilities several days after admission when they had detoxified from alcohol intake, the levels of cotinine observed in the current study are probably not representative of daily cigarette consumption prior to admission. Nine of the smokers had positive tests for cannabis consumption.

**Table 2:** Clinical and demographic characteristics of subjects included in main analysis.

	Non-smokers	Smokers
***N***	35	26
**Age**	47 ± 8	46 ± 7
**Gender**		
Male	28	18
Female	7	8
**Ethnicity**		
White	33	24
African American	1	2
Hispanic	1	0
**Daily cigarette consumption in the past month**	0	20 ± 8
**Substance use status**		
Alcohol	4	24
Positive Cotinine	0	26
Positive Hydroxy THC	0	9
**Average methylation (% β)**		
cg05575921	90.3 ± 1.9	68.8 ± 11.6
cg01940273	59.8 ± 4.4	49.9 ± 4.6
cg21566642	46.2 ± 4.8	34.2 ± 7.0
cg05951221	39.5 ± 4.2	31.1 ± 5.2
cg23576855	67.0 ± 13.1	49.2 ± 11.1

**FIGURE 1:**
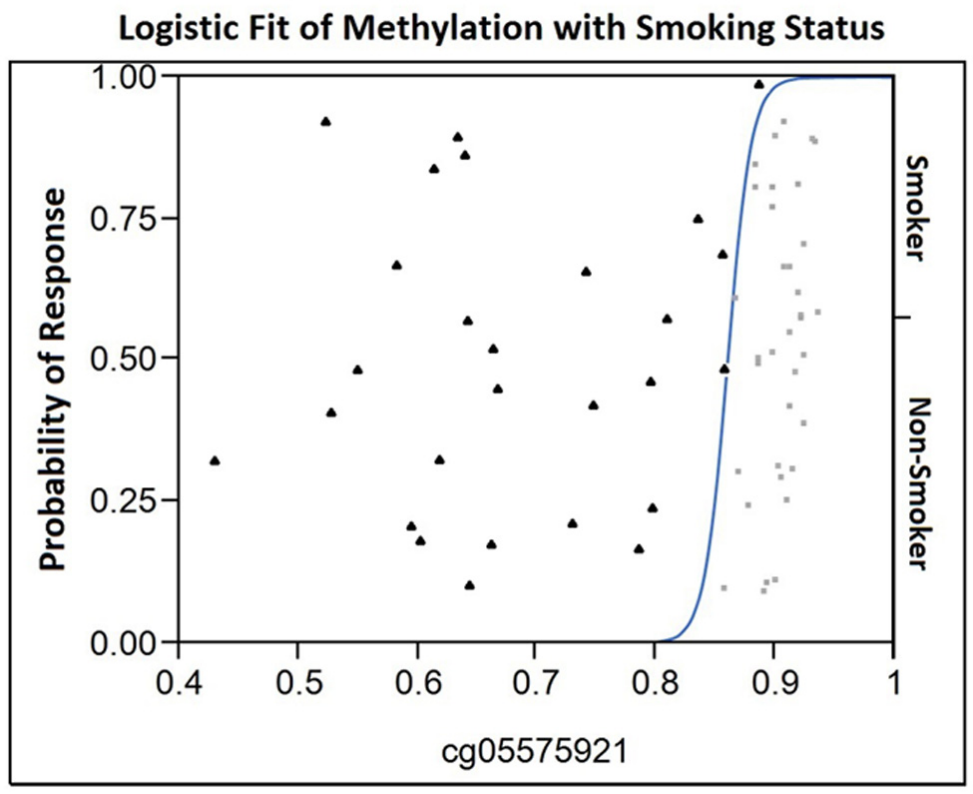
**The Logistic Fit plot of the relationship between methylation at cg05575921 and smoking status.** Observed cg05575921 methylation for each 61 subjects in the study is shown on the *X*-axis (smokers are represented by a filled triangle, non-smokers by a light gray box) with their position on the *Y*-axis not having meaning. In contrast, the position on the *Y*-axis with respect to the fitted curve indicates the probability for each response (either smoker or non-smoker) for a given value on the *X*-axis. In essence, for those subjects with a methylation level less than 0.82 have essentially no chance of being in the non-smoker group. At a methylation level of approximately 0.85, the probability of being a smoker is approximately 0.75 and the probability of being a non-smoker is 0.25. Then, the probability of being non-smoker then rises as a function of increasing methylation becoming virtually certain at levels of 0.9 or greater.

The loci selected for this study are the five most commonly replicated loci and the only five loci that are consistently demethylated in both European and African American populations (Dogan et al., in submission). As a first step of our analyses, we conducted ANOVA analysis of the case and control data using methylation at these loci as the dependent variable (see **Figure 1**). Overall, the model that included cg05575921 provided the best fit and the largest arithmetic differences (21%) between cases and controls (adjusted *R*^2^ = 0.66). The results from the three loci on Chromosome 2, cg01940273, cg21566642, and cg05951221 provided the next best fits with adjusted *R*^2^-values of 0.55, 0.50, and 0.44, respectively. However, the differences in the means of the Chromosome 2 loci were much more modest, ranging from approximately 8–10%. Finally, the model that used data only from cg23576855 was the worst fit with an adjusted *R*^2^ of 0.34. Consistent with recent studies showing that methylation in these arrays and at this locus in particular is often affected by local genotype (Shenker et al., 2012; Philibert et al., 2014b) visual inspection of the data showed strong evidence of GxMeth effects with respect to smoking (data not shown).

Although our smoking subjects did not exclusively smoke cigarettes, the main mode of tobacco consumption for our subjects was cigarette smoking. Therefore, we next analyzed the relation between DNA methylation at each locus with self-reported average smoking in the past month and past year using a linear bivariate fit model. In general, methylation at cg05575921 produced the best fit, with the three Chromosome 2 probes producing intermediate levels of fit, and cg23576855 produced the worst fit (see **Table 3**). In attempts to improve the goodness of fit of the model, we then tested whether log transformation of either absolute methylation or number of cigarettes consumed could improve the fit of the models. Unfortunately, no consistent improvements in model effects were obtained.

**Table 3:** The relationship of DNA methylation to average cigarette consumption in the past month and past year.

	Adjusted *R*^2^ of linear fit model for average cigarette consumption	
CpG probe	Past month	Past year
cg05575921	0.64	0.63
cg01940273	0.57	0.56
cg21566642	0.46	0.45
cg05951221	0.47	0.45
cg23576855	0.30	0.32

*The adjusted *R*^2^ is based on a linear fit model.*

As the final step of our analyses, we used data from all five loci alone and in combination with one another, in an attempt to determine whether data from a single marker or multiple markers is optimal for the discrimination of smokers from non-smokers. When only single markers were considered, receiver operating characteristic model (ROC) analyses of the data showed that cg05575921 provided the best discrimination with area under the curve (AUC) of 0.99 (**Table 4**). Review of the logistic fit curve for cg05575921 with respect to smoking status shows excellent sensitivity for these smokers at all ranges of specificity (**Figure 1**). DNA methylation at the other four loci, in particular, cg01940273 were slightly less discriminative with the use of a two marker set consisting of the Chromosome 2 probe cg01940273 and the AHRR probe cg23576855 resulting in an AUC of 0.947.

**Table 4:** Summary of ROC area under the curve (AUC) analyses for single and two marker sets.

		When combined with methylation from another locus		
	Alone	cg21566642	cg05951221	cg23576855
cg05575921	0.990			
cg01940273	0.940	0.939	0.946	0.947
cg21566642	0.905		0.918	0.923
cg05951221	0.903			0.919
cg23576855	0.860			

## Discussion

In this limited, but well characterized set of participants, we show that DNA methylation status at cg05575921, and to a lesser extent at three Chromosome 2 loci, can be used to accurately quantify the amount of smoking. Important limitations of the current study include sample size and the limited diversity in the subject pool.

To a large extent, the strength of the findings is in large part due to the careful selection and characterization of the participants. In our experience with several large cohorts of subjects from longitudinal studies, we often find that participants who deny ever smoking cigarettes often have markedly elevated levels of cotinine in their serum and have medical illnesses, such as chronic obstructive lung disease (COPD), that are generally found in association with smoking. Review of the literature suggests that our experience is not unique. In 2007, Gorber et al. (2009) conducted a meta-analysis of 67 studies of the relationship between self-reported smoking status and smoking status as determined by serum, urine, or salivary cotinine levels. They found trends of underestimation of the true rate of smoking when smoking status is based only on self-report depending on the population studied. These findings confirmed the earlier results from Fendrich et al. (2005) who found that the sensitivity of self-report in a large (*n* = 627) cohort from an epidemiological study was less than 90%, even after generous compensation for passive exposure. In order to minimize the likelihood that our controls smoked, we recruited our controls from the employee pool of our hospital complex which forbids smoking. Even so, it is notable that one of the participants who reported no smoking history had a positive test for cannabis consumption. Hence, the level of methylation at cg05575921 observed in the non-smoking participants in our study (β% of 90.3 ± 1.9) is probably an accurate reflection of adult methylation values in the complete absence of smoking and highlights the need for intense scrutiny and serum confirmation of non-smoking controls. In this regard, the substance use status participants from almost all of the studies listed in **Table 1** were not biochemically verified. Hence, it is likely that small numbers of smokers were misclassified and that as a result, the average β-values for the non-smoking groups were underestimated.

Even though several of the loci showed considerable promise for possible clinical translation, it is important to realize that the ROC AUC calculations were conducted using data from methylation microarrays. This hybridization-based approach is performed under meticulous conditions and takes several days to complete. After the assessment is complete, sophisticated computational processing is then required to extract the normalized β-values. It is unlikely that this assessment approach can be adapted to the point of care (POC) or hospital-based pathology lab practice.

In contrast, quantitative PCR (qPCR) techniques are becoming increasingly common in clinical settings (Lorincz, 2014). At least one epigenetic diagnostic is already FDA approved, and it is likely that several others will gain approval in the near future (Heichman, 2014; Lorincz, 2014). Like all qPCR assessments, the power of these tests to distinguish groups from one another is dependent on the variability of the assay itself, and the absolute difference between the two groups. In normal practice, inter-assay variability of approximate 1–2% is routinely observed for most qPCR assays. Because the average difference at cg05575921 between adult smoker and non-smokers is approximately 21%, while the absolute difference at the three Chromosome 2 loci is only 8–10%, it is readily apparent that the AHRR site is a better choice for clinical systems. This is why our initial assay was targeted at this locus (Dogan et al., 2014). However, with the appropriate amount of effort, it is still may be feasible to pursue clinical tests for adults based on the other loci. Unfortunately, this is not the case for any diagnostic targeted at adolescents because the magnitude of change at the Chromosome 2 loci in nascent smokers is only on the order of 1–2% (Philibert et al., 2012, 2013). In contrast, the change at cg05575921 is much more robust (5–10%) and is a much more suitable locus for detection of adolescent smokers.

This methylation-based assessment technique could be particularly valuable for understanding the relation between the use of e-cigarettes and cigarette smoking. The changes in AHRR methylation are not secondary to nicotine consumption itself. Rather, AHRR methylation is an exquisite indicator of exposure to the dioxins and polyaromatic hydrocarbons (PAH) found in cigarette smoke. Indeed, in the current study, the two subjects excluded from our study secondary to the use of “chew” had the exact same methylation at cg05575921 (90.3 and 90.9 β%) as our non-smoking controls (β% of 90.3 ± 1.9) confirming prior findings by Besingi and Johansson (2014) that nicotine ingestion itself has no effect on AHRR methylation. Although it is true that the heat filament induced vaporization of the propylene glycol solution also produces small amounts of potentially concerning byproducts, the extent of these pollutants, in particular dioxins and PAH, appears to be relatively small (McAuley et al., 2012; Schober et al., 2014). To date, the most incriminating study of dioxins or PAH in e-cigarette aerosols showed a total of 96 ng of PAH and no dioxins being produced from the pyrolysis of an entire e-cigarette cartridge (equivalent to the puffs of about 15 cigarettes; Laugesen, 2008). For the sake of reference, this corresponds to approximately 11 pg/ml in the typical 35 ml puff-, which is about the 30 times the PAH content in urban or rural air (Li et al., 2005; Primbs et al., 2008). Because the average human breathes 8–12 times per minute with an average tidal volume of 500 ml, smoking “e-cigarettes” essentially doubles the amount of PAH inhaled *only* while smoking the e-cigarette. In contrast, the PAH just the mainstream smoke of the equivalent number of cigarettes is between 15000 and 24000 ng of PAH (Ding et al., 2005). Hence, those who smoke e-cigarettes only should not have an appreciable change at cg05575921 but have positive cotinine levels while those who are smoking real cigarettes will have both changes at cg05575921 and a positive cotinine level. Therefore, the amount of incorporation of DNA methylation assessments into research protocols could provide valuable biological information to longitudinal studies of the relationship of e-cigarette use to subsequent cigarette smoking.

An additional boon to potential clinical translation is the fact that methylation in DNA from blood is closely correlated to that obtained from saliva. In fact, one recent study that provided analyses of paired samples from the same person demonstrated a correlation of 0.90 of cg05575921 methylation in DNA drive from blood and saliva (Smith et al., 2015). Unfortunately, unlike blood, the principal cell components found in saliva differ significantly with respect to their methylation set point at this locus. Therefore, techniques that can compensate for cellular heterogeneity will be required before saliva DNA methylation approaches can be used alongside blood-based approaches in the assessment of smoking status. Our group is currently working on one such technique.

Somewhat ironically, these methylation assessments may increase our ability to improve self-report measures. It goes without saying that bad questions asked poorly illicit are likely to elicit unreliable answers. A shortcoming of prior assessments of self-report reliability with respect to adolescent smoking was that the methods to assess reliability themselves seldom performed objective testing and when they did they only tested cotinine levels (Gorber et al., 2009). The current findings suggest that the addition of methylation assessments may increase our confidence in identifying true positives and true negatives, resulting in an improved mechanism through which to evaluate methods of obtaining substance use histories.

A critical question not addressed in this manuscript is whether changes in DNA methylation at cg05575921 can be used as a marker of smoking cessation. Already, three independent studies have shown that this is also the locus that shows the most significant change in response to cessation of smoking. There are two principal challenges to the use of methylation status at this AHRR locus in this regard. First, since the average methylation for heavy smokers seems to vary widely, any assessment of tobacco cessation will have to take into account the initial methylation status of the patient in question. Second, the half-life for decay of the smoking induced changes at this locus will have to be much better characterized. All three of the studies that showed the primacy of cg05575921 remethylation in response to smoking cessation were based solely on self-report data. Since the self-reports of “former smokers” can be unreliable as to the extent and timeframe of smoking cessation (Attebring et al., 2001), and the true “set point” of cg05575921 is still being refined, examination of this phenomenon in large, well-characterized samples (i.e., frequent biochemical validation) will be required before the viability of this approach for assessing smoking cessation can be considered. Still, given the positive response of smokers to biofeedback information from exhaled carbon monoxide measurements, the possibility that patients could gain enhanced motivation to quit smoking by seeing methylation changes at loci, such as F2RL3, which is implicated in heart disease risk (Breitling et al., 2012; Zhang et al., 2014), as a function of smoking cessation suggests that this possibility deserves further exploration. Currently, in efforts funded by the National Institute of Drug Abuse, our consortium is pursuing a small pilot study to explore the feasibility of this approach.

In summary, using data from well-characterized, biochemically verified participants, we show that DNA methylation assessments, particularly at cg05575921, are very sensitive and specific indicators of smoking status in adults. We suggest that additional study of large, well characterized, biochemically confirmed, epidemiological representative populations are the next logical step in the translation of this approach into routine clinical, research, and commercial usage.

## Conflict of Interest Statement

Dr. Robert Philibert is the majority owner and Chief Scientific Officer of Behavioral Diagnostics. He has pending and granted intellectual property rights for the use of DNA methylation techniques to quantify substance use consumption. Dr. Terry Osborn is the Chief Excecutive Officer and partial owner of Behavioral Diagnostics.
