# Supplementary material for: A quantitative epigenetic approach for the assessment of cigarette consumption
Source: Front Psychol. 2015 Jun 2;6:656. doi: 10.3389/fpsyg.2015.00656 (PMC4451580; doi:10.3389/fpsyg.2015.00656)
Supplement: Supplementary file 1 [file Data_Sheet_1.PDF]

SEMI STRUCTURED ASSESSMENT
FOR THE GENETICS OF ALCOHOLISM - II

SSAGA-II

The SSAGA-II is an instrument designed to assess physical, psychological and social manifestations of alcoholism and related disorders. It is a semi-structured interview which capitalizes on prior research in psychiatric epidemiology. As such, it relies on items previously validated by other research interviews, including the DIS, CIDI, HELPER, SAM, SADS, and SCID.

The members of the COGA Assessment Committee are indebted to the many researchers who developed the interviews upon which, in part, the SSAGA-II is based.

RESPONDENT'S I.D.: ____ ____ ____ ____ ____ ____ ____ ____

INTERVIEWER'S I.D.: ____ ____

DATE OF INTERVIEW: ____ ____/ ____ ____/ ____ ____ ____ ____
MO DAY YEAR

TIME STARTED: ____ ____:____ ____ (USE 24 HOUR CLOCK)

TIME ENDED: ____ ____:____ ____ (USE 24 HOUR CLOCK)

DATE EDITED: ____ ____/ ____ ____/ ____ ____ ____ ____
MO DAY YEAR

DATE ENTERED: ____ ____/ ____ ____/ ____ ____ ____ ____
MO DAY YEAR

CONTENTS

A DEMOGRAPHICS 3

B MEDICAL HISTORY 16

D TOBACCO 26

E ALCOHOL 32

F MARIJUANA 57

G DRUGS 68

I DEPRESSION 88

K MANIA 104

L PSYCHOSIS 117

M ANTISOCIAL PERSONALITY 123

O POST-TRAUMATIC STRESS DISORDER 137

Q OBSESSIVE-COMPULSIVE DISORDER 143

R SOCIAL PHOBIA 147

S AGORAPHOBIA 150

T PANIC 153

W SUBJECT COMMENTS 157

X INTERVIEWER OBSERVATIONS 158

TIME STARTED: _____:_____

(USE 24 HR CLOCK)

RECORD ITEMS MARKED "*t*" ON THE TIMELINE.

A1	RECORD SEX AS OBSERVED.	MALE ....1<br>FEMALE 2
A2	How tall are you?	___ ___ ___<br>FT IN
A3	How much do you weigh?	___ ___ ___ LBS
	A. What is the most you have <u>ever</u> weighed (when you were not pregnant)?	___ ___ ___ LBS
	B. How old were you when you first weighed (# LBS. IN A) (when you were not pregnant)?	___ ___ AGE
A4	How old are you now?	___ ___ AGE

A5 What is your birth date? ___ ___/___ ___/___ ___ ___ ___

MO DAY YEAR

A6 Were you adopted?

NO 1

YES 5

A7 Are you a twin or other multiple?

NO 1

YES 5

HAND R CARD A1.

A8 A. This card has the names of some racial groups. CODE: ___ ___

To which group do you belong?

IF OTHER, SPECIFY:___________________

HAND R CARD A2.

	I	II
B. This card is a list of origins and descents. What is the origin or descent of your grandparents? Let's start with your mother's		
MATERNAL GRANDMOTHER	__ __	__ __
MATERNAL GRANDFATHER	__ __	__ __
PATERNAL GRANDMOTHER	__ __	__ __

mother. PATERNAL GRANDFATHER

__ __ __ __

IF KNOWN, RECORD THE GRANDPARENT'S FATHER IN COL. I

AND THE GRANDPARENT'S MOTHER IN COL. II.

C. What is your religious preference?

IF NONE, SKIP TO A9. CODE: ___ ___

RECORD: ________________

1. Does your religion have rules forbidding the use of any alcohol?

NO...1

YES 5

D. In the past twelve months, how many times did you attend religious services? ___ ___ ___ TIMES

A9	Are you presently married or are you widowed, separated, divorced, or have you never been married?<br><br>**CODE RELIGIOUS ANNULMENT AS DIVORCED. CODE LEGAL ANNULMENT AS NEVER MARRIED.**	MARRIED..............................1<br>WIDOWED . . .(CODE YR).... 2<br>___ ___ ___ ___YEAR *t*<br>SEPARATED......................... 3<br>DIVORCED .... 4<br>NEVER MARRIED................. 5
A10	Have you ever lived with someone (else) for at least a year as though you were married? **DO NOT COUNT INDIVIDUALS R HAS MARRIED.**	NO ....1<br>YES 5

BOX A10 IF R NEVER MARRIED (A9=5), SKIP TO BOX A12.

A11 How many times have you been legally married?

___ ___ TIMES

YEARS OF ALL MARRIAGES

___ ___ ___ ___ YR *t*

___ ___ ___ ___ YR *t*

___ ___ ___ ___ YR *t*

___ ___ ___ ___ YR *t*

ASPFGN

A12 (So you've never been/How many times have you been) divorced? **IF NEVER, CODE 00.**

___ ___ TIMES

YEARS OF ALL DIVORCES

___ ___ ___ ___ YR *t*

___ ___ ___ ___ YR *t*

___ ___ ___ ___ YR *t*

___ ___ ___ ___ YR *t*

BOX A12 IF R NEVER MARRIED (A9=5) AND NEVER COHABITATED (A10=1), SKIP TO A14. OTHERS CONTINUE.

ASPFGN

A13 (Other than when you separated just before a divorce,) have you and your partner(s) ever separated for 3 days or longer because of not getting along?

NO. . . . . .(SKIP TO A14) 1

YES 5

ASPFGN

A. How many times did you separate? **COUNT ALL MARRIAGE AND LIVE-IN SITUATIONS.**

___ ___ TIMES

B. How old were you the last time you separated?

AGE REC: ___/___

REC: 1 2 3 4 5

BOX A14	IF R IS MALE, SKIP TO A14C.

A14 How many times have you been pregnant? ___ ___ TIMES

IF NEVER, SKIP TO A15.

A. Are you currently pregnant?

NO 1
YES 5

B. How many stillbirths and miscarriages have you had? ___ ___ NUMBER

C. How many children have you had, not counting any who are yours by adoption, who are stepchildren, or who were stillborn? **RECORD SEX AND DOB.**

___ ___ CHILDREN

	DATE OF BIRTH				DATE OF BIRTH	
SEX	MO	YEAR		SEX	MO	YEAR
M F	___ ___/	___ ___ ___ ___	*t*	M F	___ ___/	___ ___ ___ ___ *t*
M F	___ ___/	___ ___ ___ ___	*t*	M F	___ ___/	___ ___ ___ ___ *t*
M F	___ ___/	___ ___ ___ ___	*t*	M F	___ ___/	___ ___ ___ ___ *t*
M F	___ ___/	___ ___ ___ ___	*t*	M F	___ ___/	___ ___ ___ ___ *t*

A15 What is the highest grade in school you completed?

CODE ACTUAL GRADE (00-17).

___ ___ GRADE

IF A15 IS 12 OR LESS, ASK A.

OTHERS SKIP TO C.

TECHNICAL SCHOOL OR 1 YR COLLEGE.................................... 13

2 YRS COLLEGE......................... 14

3 YRS COLLEGE......................... 15

4 YRS COLLEGE: B.A., B.S....... 16

GRADUATE: M.A., M.S., J.D., M.D., Ph.D. 17

A. Do you have a high school diploma?

NO..1

YES. . . . . (SKIP TO C)........ 5

B. Did you pass a high school equivalency test (GED)?

NO...1

YES 5

C. Did you graduate from the last school you attended?

NO...1

YES 5

D. When did you graduate from ...

HIGH SCH:___ ___ ___ ___ YR *t*

GED: ___ ___ ___ ___ YR *t*

COLLEGE:___ ___ ___ ___ YR *t*

GRAD:___ ___ ___ ___ YR *t*

OTHER:___ ___ ___ ___ YR *t*

NO..1

E. Are you currently in school, in a program leading to a degree?

YES 5

A16 Now I want to ask you about work for pay. In the past twelve months, how many months have you been employed? **COUNT SELF-EMPLOYMENT OR SALARIED. IF NONE, CODE 00 AND SKIP TO A17B. IF LESS THAN 1 MONTH, CODE 01.**

___ ___ MONTHS

A17 Are you employed now?

NO. . . . . . . (SKIP TO B).......1

YES 5

A. Do you work full-time?

NO..1

YES 5

B. What is your current <u>household gross</u> income?

CODE: ___ ___

HAND R CARD A3.

$0-$192/week -$0-$833/month	$0-$9,999/year	01
$193-$384/week -834-$1,666/month	$10,000-$19,999/year	02
$385-$576/week -$1,667-$2,499/month	$20,000-$29,999/year	03
$577-$769/week-$2,500-$3,333/month	$30,000-$39,999/year	04
$770/$961/week -3,334-$4,166/month	$40,000-$49,999/year	05
$962-$1,442/week-4,167-$6,249/month	$50,000-$74,999/year	06
$1,443-$1,923/week-$6,250-8,333/month	$75,000-$99,999/year	07
$1,924-$2,884/week-$8,334-2,499/month	$100,000-$149,999/year	08
$2,885+/week-$12,500 +/month	$150,000 or more/year	09

A18	Have you ever been on active duty in the military? **NATIONAL GUARD AND RESERVES ARE NOT CONSIDERED ACTIVE DUTY UNLESS OFFICIALLY ACTIVATED.**	NO. . . (SKIP TO B1, p. 5)......1<br>YES 5

A. What kind of discharge did you have?

OTHER THAN HONORABLE INCLUDES WITHOUT HONOR AND UNDESIRABLE.

STILL IN THE MILITARY 0
HONORABLE 1
GENERAL 2
MEDICAL 3
OTHER THAN HONORABLE 4
DISHONORABLE 6

B. What's the highest rank you achieved?

1. RANK:

______________________________ CODE: ___ ___ ___

_____

2. BRANCH OF MILITARY:

____ CODE: ___

B1	Now I have some questions about your physical health and medical history. First, at the present time, would you say your health is excellent, very good, good, fair, or poor?	EXCELLENT .... 1<br>VERY GOOD .... 2<br>GOOD .... 3<br>FAIR .... 4<br>POOR 5
B2	Has your health always been (ANSWER IN B1), or has it been better or worse?<br><br>A. Please explain: ______________________________	NO, WORSE .... 1<br>NO, BETTER .... 2<br>YES, SAME . . (SKIP TO B3) 5<br>BOTH BETTER & WORSE ... 6

B3 Has a doctor ever told you that you have (had):

	NO	YES	YEAR DIAGNOSED
1. High blood pressure?	1	5	— — — —
2. Migraine headaches?	1	5	— — — —
3. A brain injury or concussion?	1	5	— — — —
4. Been unconscious for longer than 5 min?	1	5	— — — —
5. Epilepsy or have had a seizure?	1	5	— — — —

6.	Meningitis or encephalitis?	1	5	__ __ __ __
7.	A stroke?	1	5	__ __ __ __
8.	Heart disease?	1	5	__ __ __ __
9.	Liver disease?	1	5	__ __ __ __
10.	Thyroid disease?	1	5	__ __ __ __
11.	Asthma?	1	5	__ __ __ __
12.	Diabetes?	1	5	__ __ __ __
13.	Cancer?			
	**SPECIFY:**_________________________	1	5	__ __ __ __
14.	HIV/AIDS?	1	5	__ __ __ __
15.	A sexually transmitted disease?	1	5	__ __ __ __
16.	Any other illness(es)?	1	5	__ __ __ __
	_________________________			
17.	Other?	1	5	__ __ __ __
	______________________________________			

B4 A. How many times have you been in a hospital overnight (including surgery and pregnancy), <u>excluding</u> psychiatric or substance abuse treatment? ___ ___ TIMES

Please tell me about your hospital stays, starting with the most recent one.

YEAR	LENGTH OF STAY (DAYS)	REASON FOR HOSPITALIZATION	HOSPITAL/FACILITY CITY/STATE	ADM. PHYSICIAN & SPECIALTY
___ ___ ___ ___	_____	____________________	____________________	________________
___ ___ ___ ___	_____	____________________	____________________	________________
___ ___ ___ ___	_____	____________________	____________________	________________
___ ___ ___ ___	_____	____________________	____________________	________________

B4 B. How many times have you had surgery when you did not have to stay in a hospital overnight (that is, outpatient surgery)? ___ ___ TIMES

C. How many times have you been examined or treated in the emergency room because of an accident or injury? ___ ___ TIMES

B5 In the last 6 months, how many visits have you made to a doctor, clinic, or emergency room for your physical health? **DO NOT COUNT CHIROPRACTORS.** ___ ___ VISITS

B6 A. Have you ever taken any prescription medications for two weeks or longer . . . (READ 1-7)

IF YES, ASK: What did you take? **DO NOT COUNT OTC.**

	NO	YES	MEDICATIONS	CODE #1	CODE #2
1. To make you feel less nervous?	1	5	________________	__ __ __	__ __ __
2. To help you sleep?	1	5	________________	__ __ __	__ __ __
3. To feel less depressed?	1	5	________________	__ __ __	__ __ __
4. For headaches?	1	5	________________	__ __ __	__ __ __
5. To have more energy?	1	5	________________	__ __ __	__ __ __
6. For birth control?	1	5	________________	__ __ __	__ __ __
7. Containing steroids?	1	5	________________	__ __ __	__ __ __

IF ALL ARE CODED 1, SKIP TO B6B.8.

FOR EVERY 5 CODED IN B6A.1-7, ASK B6B.1-7, AND ASK B6B.8.

B. In the last 30 days, have you taken any prescription medications for two weeks or longer . . .

IF YES, ASK: What did you take? **DO NOT COUNT OTC.**

	NO	YES	MEDICATIONS	CODE #1	CODE #2	
1. To make you feel less nervous?	1	5	____________	__ __ __	__ __ __	
2. To help you sleep?	1	5	____________	__ __ __	__ __ __	
3. To feel less depressed?	1	5	____________	__ __ __	__ __ __	
4. For headaches?....	1	5	____________	__ __ __	__ __ __	
5. To have more energy?	1	5	____________	__ __ __	__ __ __	
6. For birth control?	1	5	____________	__ __ __	__ __ __	
7. Containing steroids?	1	5	____________	__ __ __	__ __ __	
8. For anything else? .(SPECIFY) .	1	5	____________	__ __ __	__ __ __	____

IF YES, SPECIFY REASON(S): ____________ B6B.8: CODE #4 __ __ __ CODE #5 __ __

B7 Have you ever had any emotional problems or times that stand out as particularly troubling or upsetting during your life?

NO. . . . . (SKIP TO B8).........1

YES 5

IF YES: Would you tell me about this?

B8 Have you ever spoken to a professional about any emotional problems you might have had?

NO. . . . . (SKIP TO B9)................1
YES 5

A. Did you speak to a ...?

	NO	YES
1. Psychiatrist	1	5
2. Psychologist	1	5
3. Social worker	1	5
4. Counselor	1	5
5. Other medical doctor	1	5
6. Nurse practitioner	1	5
7 Clergy	1	5
8. Other: ______________________	1	5

B9 How many times have you been an inpatient in a psychiatric hospital or ward or in a chemical dependency program where you stayed overnight?

___ ___ TIMES

IF NEVER, SKIP TO B10.

A. When was the first time you were treated as an inpatient? __ __ / __ __ __ __ *t*

MO YEAR

REASON FOR TREATMENT CODES:

1 = Psychiatric (non-alcohol or drug)

2 = Alc/Drug Treatment

3 = Combined Psychiatric & A/D Txmt

Please tell me about your inpatient stays, starting with the most recent one:

YEAR	LENGTH OF STAY (DAYS)	REASON FOR TREATMENT	REASON CODE	HOSPITAL/FACILITY CITY/STATE	ADM. PHYSICIAN & SPECIALTY
___ ___ ___ ___ *t*	_____	__________________	1 2 3	__________________	____________________
___ ___ ___ ___ *t*	_____	__________________	1 2 3	__________________	____________________
___ ___ ___ ___ *t*	_____	__________________	1 2 3	__________________	____________________
___ ___ ___ ___ *t*	_____	__________________	1 2 3	__________________	____________________

B10 Have you ever received outpatient treatment for psychiatric, emotional, or chemical dependency problems? This includes any visits to a psychiatrist, psychologist, therapist, or counselor.

NO . . .(SKIP TO BOX B11).....1
YES 5

CODES FOR NUMBER OF VISITS:

1= 1-10 visits
2= 11-20 visits
3= more than 20 visits

REASON FOR TREATMENT CODES:

1= Psychiatric (non-alcohol or drug)
2= Alc/Drug Treatment
3= Combined Psychiatric & A/D Txmt

Please tell me about your outpatient treatment, starting with the most recent one:

YEAR	NUMBER OF VISITS	REASON FOR TREATMENT	REASON CODE	HOSPITAL/FACILITY CITY/STATE	ADM. PHYSICIAN & SPECIALTY
___ ___ ___ ___	1 2 3	________	1 2 3	________	________
___ ___ ___ ___	1 2 3	________	1 2 3	________	________
___ ___ ___ ___	1 2 3	________	1 2 3	________	________
___ ___ ___ ___	1 2 3	________	1 2 3	________	________

BOX B11 A. IS R CURRENTLY IN TREATMENT?

NO 1
YES 5

B. DOES R VOLUNTEER MORE THAN 4 SEPARATE OUTPATIENT TREATMENT PROGRAMS?

NO 1
YES 5

Now I'm going to ask you some questions about using tobacco.

(3) D1 A. Have you ever tried any form of tobacco? — NO... 1 / YES(SKIP TO C) 5

B. So, you never have experimented with any form of tobacco (including cigarettes) even one time? — NEVER. . . .(SKIP TO E1, p. 32) 1 / YES, HAS USED 5

C. Have you ever:

	NO	YES
1. smoked a cigarette?...	1	5
2. smoked a cigar?	1	5
3. smoked a pipe?	1	5
4. used chewing tobacco or snuff?..............................	1	5

D. How old were you the (first/last) time you used any form of tobacco?

AGE ONS: ___/___
ONS: 1 2 3 4 5

AGE REC: ___/___
REC: 1 2 3 4 5

---

(4) D2 OMITTED

IF NEVER SMOKED CIGARETTES (D1C.1=1), CODE D3 "NO" SILENTLY.

(5) D3 Over your lifetime, have you smoked a total of 100 cigarettes? — NO(SKIP TO E1, p. 32) 1 / YES . 5

BEGIN SCORING ASTERISKED ITEMS ON TALLY SHEET D.

(1 & 6) D4 A. When you were smoking regularly, how many days per week did you usually smoke cigarettes? — ___ DAYS
IF NOT AS OFTEN AS ONCE A WEEK, CODE 0.

ND45(=20) B. How many cigarettes did you usually smoke in a day? — ___ ___ CIGS *
IF 20 OR MORE CIGS 2+ DAYS PER WEEK, MARK TALLY SHEET D.

C. For about how long did you smoke this many cigarettes at that rate? — __ __ __ UNITS

CODE UNITS:
DAYS 1
WEEKS 2
MONTHS 3
YEARS 4

	D. How old were you the (first/last) time you smoked cigarettes at that rate?	AGE ONS: ___/___<br>ONS: 1 2 3 4 5<br><br>AGE REC: ___/___<br>REC: 1 2 3 4 5

Think about the period lasting a month or more when you were smoking the most.

(7)	D5	During this period when you were smoking the most, about how many minutes after you woke up did you smoke your first cigarette? **IF DK, ASK A. OTHERS SKIP TO D6.**	___ ___ ___ MINUTES
		A. **IF DK:** Was it usually (READ OPTIONS)?	WITHIN 5 MINUTES 1<br>WITHIN 6-30 MINUTES 2<br>WITHIN 31-60 MINUTES 3<br>MORE THAN ONE HOUR 4
(8)	D6	During the period when you were smoking the most, did you usually smoke more frequently during the first hours after waking than during the rest of the day?	NO1<br>YES 5
(9)	D7	During the period when you were smoking the most, did you usually find it difficult to keep from smoking in places where it was forbidden; for example, on airplanes, in movie theaters, in "no smoking" sections of restaurants or office buildings, or perhaps in situations where someone asked you not to?	NO1<br>YES 5
	D8	During the period when you were smoking the most, which cigarette would you have hated most to give up: the first one in the morning, after eating, while watching television, or some other one?	FIRST ONE IN MORNING . . .. 5<br>ANY OTHERS 1
(10)	D9	During the period when you were smoking the most, were there times you smoked even when you were so ill that you had to be in bed most of the day?	NO1<br>YES 5

Now I'd like you to think about your cigarette smoking throughout your life as I ask you more questions about experiences people sometimes have when they smoke cigarettes. (Since you don't smoke now, I'd like to ask you about the times when you used to smoke cigarettes.)

(11)	D10	Did you ever chain smoke; that is, where you smoked several cigarettes, one right after another?	NO. . . . . . (SKIP TO D11) 1<br>YES 5
		A. For how many hours in a row did you smoke like that? **CODE LESS THAN 1 HOUR = 00.**	___ ___ HOURS

BOX D10 IF LESS THAN 3 HOURS, SKIP TO D11.

ND45		B. What is the longest period of time you have chain smoked every day or nearly every day?<br>**IF 7 OR MORE DAYS, MARK TALLY SHEET D.**	___ ___ ___ UNITS<br>**CODE UNITS:**<br>DAYS 1<br>WEEKS 2 *<br>MONTHS 3 *<br>YEARS 4 *
(12)<br>ND46	D11	Have you <u>often</u> given up or spent much less time in activities important to you such as work, sports, going to movies, or seeing friends or relatives because you would not be able to smoke?	NO 1<br>YES 5*

---

(13)<br>ND43	D12	Have you <u>often</u> smoked a lot more than you intended or for more days in a row than you intended? For example, smoking half a pack or more when trying to limit yourself to only 1 or 2 cigarettes?	NO 1<br>YES 5*
ND43		A. Have you <u>often</u> found that you've run out of cigarettes sooner than you intended?	NO 1<br>YES 5*
(14)<br>ASP3RC7<br>ASP4A5	D13	Have you smoked in situations where it was dangerous to smoke; for example, smoking in bed, when getting gasoline, or when using paint thinners or cleaning fluids?	NO (SKIP TO D14) 1<br>YES 5
		A. Did this happen a total of 3 or more times?	NO (SKIP TO D14) 1<br>YES 5
		B. Did this ever happen 3 or more times in any 12-month period?	NO 1<br>YES 5
(15)<br>ND44	D14	Have you <u>often</u> wanted to quit or cut down on smoking?<br>**SPECIFY** (DO <u>NOT</u> COUNT PREGNANCY):__________<br>____	NO 1<br>YES (SPECIFY) 5*
		A. Have you ever tried to quit smoking?	NO (SKIP TO D15) 1<br>YES 5
		B. How many times did you try to quit?	___ ___ ___ TIMES
		C. Were you <u>always</u> able to stop or cut down when you tried to?	NO (SKIP TO D) 1<br>YES 5
		1. Was this for at least 1 month?	NO 1<br>YES (SKIP TO D15) 5
ND44		D. Have you 3 or more times found that you were unable to stop or cut down on smoking (for at least 1 month)?	NO 1<br>YES 5*

---

(16)

___ ___ ___ UNITS
CODE UNITS:
DAYS 1
WEEKS 2
MONTHS 3
YEARS 4

D15 Since you began smoking regularly, what is the longest period of time you have gone without using any form of tobacco for any reason, like when you had an illness, or lost interest in tobacco, or intentionally quit? **IF NEVER, CODE 000 DAYS. IF LESS THAN ONE DAY, CODE 001 DAY.**

**BOX D15**	**IF D15 = 000 DAYS, SKIP TO D17. OTHERS CONTINUE.**

(23)

A. Have you ever attended a class or group for people trying to quit or reduce their use of tobacco? — NO 1, YES 5

B. Have you ever tried nicotine gum or a nicotine patch (to quit or reduce your use of tobacco)? — NO 1, YES 5

C. Have you ever tried nicotine-free cigarettes (to quit or reduce your use of tobacco)? — NO 1, YES 5

D. Have you tried any other form of treatment or medicine to quit or reduce your use of tobacco? — NO 1, YES (SPECIFY) 5

SPECIFY: ________________________________

____

IF ANY 5 IS CODED IN D15A-D, CONTINUE. OTHERS SKIP TO D16.

E. How old were you the (first/last) time you tried any of these methods to quit or cut down?

AGE ONS: ___/___
ONS: 1 2 3 4 5

AGE REC: ___/___
REC: 1 2 3 4 5

(17)

D16 I'm going to ask you about some problems that you might have had when you stopped smoking or smoked less tobacco than usual. Think about the time when you had the <u>most</u> problems when you went without cigarettes or had less than usual.
CODE IN COLUMN I.

I-SX
II-CLSTR
III-24HRS

During that time:	(SX) COL. I NO	YES	(CLSTR) COL. II NO	YES	(24 HRS) COL. III NO	YES
1. Were you irritable, angry, or frustrated?.........................	1	5	1	5	1	5

2.	Were you nervous or anxious?	1	5	1	5	1	5
3.	Were you restless?..	1	5	1	5	1	5
4.	Did you have trouble concentrating?.............................	1	5	1	5	1	5
5.	Did your heart slow down?	1	5	1	5	1	5
6.	Did you feel down or depressed?	1	5	1	5	1	5
7.	Did you have such a strong desire for cigarettes that you couldn't think of anything else?	1	5	1	5	1	5
8.	Did your appetite increase or did you gain weight?	1	5	1	5		
9.	Did you have trouble sleeping?	1	5	1	5		

BOX D16 HOW MANY 5'S CODED IN COLUMN I?

NONE . . . . (SKIP TO D17) 1
1-3 . . . . (SKIP TO B) 2
4 OR MORE 3

ND42A

A. Did at least four of these (SX CODED 5 IN COL. I) occur together in the first 24 hours after you stopped or cut down?

NO. . . . . . .(SKIP TO B) 1
YES 5*

1. Which ones? **CODE IN COLUMN II.**

2. How old were you the (first/last) time?

AGE ONS:
___/___
ONS:
1 2 3 4 5

AGE REC:
___/___
REC:
1 2 3 4 5

FOR EACH 5 CODED IN D16.1-7 IN COL. I, ASK B.

B. Did (SX) last for at least 24 hours?
CODE IN COL. III. ONLY COUNT SYMPTOMS THAT LAST FOR MOST WAKING HOURS.

C. Did the problems you had after quitting or cutting down on smoking <u>often</u> interfere with your work, school, or household responsibilities?

NO1
YES 5

ND42B

D. Did you start smoking again or use other sources of nicotine to avoid having the problems that quitting might cause?

NO 1
YES 5*

(18) D17 Has smoking ever made you nervous or jittery or caused you any other emotional or mental problem?

NO(SKIP TO D18) 1
YES 5

A. Did feeling nervous, jittery, or having other emotional or mental problems from smoking interfere with your functioning?

NO(SKIP TO D18) 1
YES(SPECIFY) 5

SPECIFY: ___________________________________

ND47		B. Did you continue to smoke after you knew it caused you problems like these?	NO<br>YES	1<br>5*
(19)	D18	Has smoking caused you any health problem such as a problem with your heart or blood pressure, lung trouble, a cough that wouldn't go away, or any other health problem?	NO(SKIP TO D19)<br>YES . . . . .(SPECIFY)	1<br>5
		**SPECIFY:** ______________________________________	CODE:___ ___ ___	
ND47		A. Did you continue to smoke after you knew it caused you (this/these) health problem(s)?	NO<br>YES	1<br>5*
(20)<br>ND47	D19	Have you continued to smoke when you had another serious illness that you knew was made worse by smoking, for example: asthma or bronchitis?	NO<br>YES(SPECIFY)	1<br>5*
		**SPECIFY:** ______________________________________	CODE: ___ ___ ___	
(21)	D20	A. After you had been smoking regularly for some time, did you need to increase your daily use to feel comfortable?	NO<br>YES(SKIP TO C)	1<br>5
		B. After you had been smoking regularly, did you come to need more cigarettes each day?	NO(SKIP TO D)<br>YES	1<br>5
ND41A		C. Was this 50% more? So, if you used to smoke 10 cigarettes a day, you would increase to 15 a day, or go from 20 to 30?	NO<br>YES . . (SKIP TO BOX D21)	1<br>5*
ND41B		D. After you had been smoking for some time, did you find that cigarettes had less effect on you than before?	NO<br>YES	1<br>5*

BOX D21 IF 3 OR MORE BOXES MARKED ON TALLY D, CONTINUE. OTHERS SKIP TO E1, p. 23.

HAND R TOBACCO TALLY SHEET.

(22)	D21	I'd like to review the experiences you've told me you had with smoking cigarettes. You've said that: **(READ SX MARKED ON TALLY SHEET D).**		
ND4		Did you ever have experiences from 3 or more boxes in any 12-month period? **IF YES:** Which ones? **CIRCLE THE SYMPTOMS THAT CLUSTER. MUST BE FROM 3 DIFFERENT BOXES.**	NO. . . . (SKIP TO E1, p. 32)<br>YES	1<br>5
		A. How old were you the (first/last) time?	AGE ONS: ___/___<br><br>AGE REC: ___/___	

(1) E1 Now I would like to ask you some questions about your use of alcoholic beverages, like beer, wine, wine coolers, champagne, or hard liquor like vodka, gin, or whiskey. Have you ever had a drink of alcohol?

NO 1
YES. . . . . (SKIP TO E2) 5

A. So, you have never had even one full drink of alcohol?

NEVER. (SKIP TO F1, p. 57) 1
YES, HAD A DRINK 5

---

(7) E2 I'd like to ask you about reactions that some people have when they drink any type of alcohol.

A. While drinking, has one or two drinks of alcohol ever caused you to . . . **CODE IN COL. A.**

**DO NOT COUNT IF ONLY ONE TYPE OF ALCOHOL CAUSED THE REACTION.**	COL A NO	COL A YES	COL B NO	COL B YES
1.flush or blush--that is, your face and hands felt hot and your face turned red?	1	5	1	5
a. **IF E2A.1=5, ASK**: Did the flushing or blushing begin within the first few minutes after the first drink?	1	5		
2.break out into hives?	1	5	1	5
3.feel very sleepy (when you weren't already tired)?	1	5	1	5
4.have nausea?	1	5	1	5
5.have headaches, head pounding, or throbbing?	1	5	1	5
6.have heart palpitations, where your heart beat so hard you could feel it?	1	5	1	5

FOR EACH 5 CODED IN COL. A, ASK B. OTHERS SKIP TO E3.

B. Did (SX) ever keep you from drinking any alcohol on at least one other occasion? **CODE IN COL. B.**

---

(2) E3 Did you have any drink containing alcohol in the last week?

NO…1 IF NO SKIP TO BOX E3

YES 5

A. We would like to know the number of alcoholic drinks you've had each day in the last week.

Today is _____________. Let's begin with yesterday.

How many drinks of (beer, wine, liquor) did you have on (DAY OF WEEK)?

REFER TO CARD E1 FOR THE DEFINITION OF A STANDARD DRINK.
IF OTHER, RECORD SPECIFIC DRINK NAME.

	BEER	WINE	LIQUOR	OTHER	(SPECIFY DRINK)
M	_____	_____	_____	_____	______________________
Tu	_____	_____	_____	_____	______________________
W	_____	_____	_____	_____	______________________
Th	_____	_____	_____	_____	______________________
F	_____	_____	_____	_____	______________________
Sa	_____	_____	_____	_____	______________________
Su	_____	_____	_____	_____	______________________

B. Did you have anything to drink today?

NO.. 1

YES 5

BOX E3 IF R DRANK THIS WEEK (E3=5), CODE E3C SILENTLY. OTHERS CONTINUE.

C. When was the last time you had a drink containing alcohol?

__ __ / __ __
MO YR

IF DK DATE, ASK:

AGE REC: ___/___

REC: 1 2 3 4 5

(3) E4 A. Think about your use of alcohol over the past 6 months. How many weeks in the past 6 months have been weeks in which you drank alcohol? **IF EVERY WEEK, CODE 26. IF 00, SKIP TO E5.** ___ __ WEEKS

B. We would like to know the number of drinks containing alcohol you would have each day in a typical week when you drank any alcohol. On a typical (Monday, Tuesday...) in the past 6 months, how many drinks of (beer, wine, liquor...) would you have?

REFER TO CARD E1 FOR THE DEFINITION OF A STANDARD DRINK.
IF OTHER, RECORD SPECIFIC DRINK NAME.

	BEER	WINE	LIQUOR	OTHER	(SPECIFY DRINK)
M	_____	_____	_____		____________________
Tu	_____	_____	_____		____________________
W	_____	_____	_____		____________________
Th	_____	_____	_____		____________________
F	_____	_____	_____		____________________
Sa	_____	_____	_____		____________________
Su	_____	_____	_____		____________________

C. I'd like you to think about the week in the last 6 months when you drank the most. How many days did you drink during that week? ___ DAYS
IF R VOLUNTEERS THAT NO WEEK STANDS OUT AS THE HEAVIEST (I.E., TYPICAL= HEAVIEST), CODE 0 AND SKIP TO E5.

D. How many drinks did you have on a typical day during that week? ___ __ DRINKS

E. During what month did a week like that (last) occur? ____ / ___ ___ ___ ___
MO YEAR

(4) E5 At what age did you begin to drink regularly; that is, drinking at least once a month for 6 months or more? **IF NEVER, CODE 00.**

AGE: ______

A. How old were you the first time you got drunk, that is, your speech was slurred or you were unsteady on your feet? **IF DK, ASK A1.**
IF NEVER, CODE 00 AND SKIP TO BOX E5.
IF DRUNK BEFORE AGE 15, SKIP TO B.
OTHERS SKIP TO BOX E5.

AGE: ________

1. Was it before you were 15 years old?

NO (SKIP TO BOX E5)…1
YES5

B. Did you get drunk more than once before you were 15 years old?

NO..1
YES 5

BOX E5 IF D3=5, CONTINUE. OTHERS SKIP TO E6.

C. When drinking, did you almost always smoke cigarettes at the same time? **COUNT TOBACCO ONLY.**

NO..1
YES 5

(5) E6 In your lifetime, what is the largest number of drinks you have ever had in a 24-hour period (including all types of alcohol)?

___ __ DRINKS

A. In the past 6 months, what is the largest number of drinks you've drunk in a 24-hour period?

___ __ DRINKS

BOX E7 IF E6=3 DRINKS OR FEWER (LIFETIME), SKIP TO F1, p. 57. IF E5 AND E5A BOTH CODED 00, SKIP TO F1, p. 57. OTHERS CONTINUE.

(6) E8 Was there ever a time when you drank almost every day for a week or more? By "almost every day" I mean at least 4 days out of 7.

NO (SKIP TO E9)…1
YES5

A. Think about those periods of time when you drank almost every day for at least a week (again, at least 4 out of 7 days). What was the largest number of drinks that you would drink almost every day for at least 1 week?

___ __ DRINKS

ALMOST EVERY DAY = 4 OUT OF 7.

B. So, almost every day during this period you drank <u>at least</u> (# FROM A) drinks?

NO (RE-ASK A)…1
YES5

C. How old were you when this period began? AGE ONS ___/___

ONS 1 2 3 4 5

D. How long did this period last? ___ __ WEEKS

BEGIN SCORING ALCOHOL TALLY SHEETS A, B, &C.

(20) E9 (After you started drinking regularly,) did you ever become tolerant to alcohol; that is, you drank a great deal more in order to get an effect, or found you could no longer get high on the amount you used to drink? **SHOW R CARD E2.**

NO. . . . (SKIP TO E)1

YES.. 5

A1. When you first started drinking regularly, how many drinks did it take you to get an effect? ___ ___ DRINKS

A2. After you had been drinking for some years, how many drinks did you usually need to get an effect? ___ ___ DRINKS

CODE THE TYPICAL UPPER BOUND OF TOLERANCE. DO NOT COUNT AN ISOLATED EXPERIENCE.

B. How old were you the (first/last) time you needed (# IN A2) drinks to get an effect?

AGE ONS: ____/____ 1 2 3 4 5

AGE REC: ____/____ 1 2 3 4 5

C. WAS THE INCREASE IN A2 TO 5 DRINKS (WOMEN)/ 6 DRINKS (MEN) OR MORE?

NO . . . . (SKIP TO E) 1

YES.. 5

AD3RA7
AD41A
ADICD4

A,B,C

D. WAS INCREASE 50% OR MORE? CHECK CARD E2.

NO 1

YES . . (SKIP TO E10) 5

E. Did you ever find you could drink a lot more before you got drunk?

NO . . . .(SKIP TO E10)…1

YES 5

F1. When you first started drinking regularly, how many drinks did it take you to get drunk? ___ ___ DRINKS

F2. After you had been drinking for some years, how many drinks did it take you to get drunk? ___ ___ DRINKS

CODE THE TYPICAL UPPER BOUND OF TOLERANCE. DO NOT COUNT AN ISOLATED EXPERIENCE.

G. How old were you the (first/last) time you needed (# IN F2) drinks to get drunk?

AGE ONS: ____/____ 1 2 3 4 5
ONS:

AGE REC: ____/____ 1 2 3 4 5
REC:1

AD3RA7<br>AD41B<br>ADICD4	**H.**	**WAS THE INCREASE IN F2 TO 5 DRINKS (WOMEN)/ 6 DRINKS (MEN) OR MORE?**	**NO . . . (SKIP TO E10) 1**<br>**YES.. 5**
	**I.**	**WAS INCREASE 50% OR MORE? CHECK CARD E2.**	**NO 1**<br>**YES 5 A,B,C**

---

(9)
AD3RA2/B
AD44
ADICD2

E10 Have you 3 or more times wanted to stop or cut down on drinking?
DO NOT COUNT DIETING OR PREGNANCY.

NO . . . (SKIP TO B) 1
YES 5A,B,C

A. How old were you the (first/last) time?

AGE ONS: ____/____ 1 2 3 4 5
ONS:

AGE REC: ____/____ 1 2 3 4 5
REC:

B. Have you ever tried to stop or cut down on drinking?
COUNT ANY REASON.

NO . . . . (SKIP TO E11) 1
YES …5A

AD3RA2
ALCFGNB1

C. Were you always able to stop or cut down when you tried to?

NO, UNABLE 1
YES (SKIP TO E11) 5

AD44
ADICD2

D. How many times were you unable to stop or cut down?
IF 3 OR MORE, MARK TALLY SHEETS B AND C AND SKIP TO E. IF DK, ASK D1. OTHERS SKIP TO E.

___ TIMES B,C

AD44
ADICD2

1. Was it 3 or more times?

NO 1
YES . 5 B,C

E. How old were you the (first/last) time?

AGE ONS: ____/____ 1 2 3 4 5
ONS:

AGE REC: ____/____ 1 2 3 4 5
REC:

---

(21)
ALCFGNB2

E11 Some people try to control their drinking by making rules, like not drinking before 5 o'clock or never drinking alone. Have you ever made any rules to control your drinking?

SPECIFY:______________________________

NO . .(SKIP TO E12) 1
YES . .(SPECIFY) 5

		A. How old were you the first time?	AGE ONS:<br>ONS:	____/____<br>1 2 3 4 5
(13)<br>ALCFGNB1	E12	Have you ever started drinking at times you <u>promised</u> yourself that you wouldn't, or have you ever drunk more than you intended? For example, when you decided to drink 2 drinks and ended up drinking 4 or more?	NO<br>YES	1<br>. . . (SKIP TO B) 5
		A. Have you ever continued drinking for more days in a row than you intended?	NO<br>YES	(SKIP TO E13) 1<br>5
		B. How old were you the (first/last) time?	AGE ONS:<br>ONS:<br>AGE REC:<br>REC:	____/____<br>1 2 3 4 5<br>____/____<br>1 2 3 4 5
AD3RA1/B<br>AD43<br>ADICD2		C. Did this happen 3 or more times?	NO..1<br>YES 5	A,B,C
(14)	E13	Have you ever started drinking and become drunk when you didn't want to?	NO<br>YES	(SKIP TO E14)..1<br>5
		A. How old were you the (first/last) time?	AGE ONS:<br>ONS:<br>AGE REC:<br>REC:	____/____<br>1 2 3 4 5<br>____/____<br>1 2 3 4 5
AD3RA1/B<br>AD43<br>ADICD2		B. Did this happen 3 or more times?	NO..1<br>YES 5	A,B,C
(22)	E14	Have you ever given up or greatly reduced important activities while drinking -- like sports, work, or associating with friends or relatives?<br><br>**SPECIFY:** ___________________________________<br>____	NO<br>YES	. . . (SKIP TO E15) 1<br>. . . . (SPECIFY) 5

A. How old were you the (first/last) time?

AGE ONS: ____/____ 1 2 3 4 5
ONS:

AGE REC: ____/____ 1 2 3 4 5
REC:

AD3RA5/B
AD46
ADICD5

B. Did this happen 3 or more times or for a month or more?

NO.. 1 A,B,C

YES 5

---

(15) E15 Has there ever been a period of several days or more when you spent so much time drinking or recovering from the effects of alcohol that you had little time for anything else?

NO . . . . (SKIP TO E16) 1
YES 5

AD3RA3
AD45
ADICD5

A. Did this period last for a month or more or did you have 3 or more periods like that?

NO . . . . (SKIP TO E16)…1
YES.. 5

B. How old were you the (first/last) time?

AGE ONS: ____/____ 1 2 3 4 5

AGE REC: ____/____ 1 2 3 4 5

---

(12) E16 Have you ever gone on binges or benders when you kept on drinking for 2 days or more without sobering up, except for sleeping?

NO (SKIP TO E17) 1
YES. 5

A. Did you neglect some of your usual responsibilities then?

NO . . . (SKIP TO E17) 1
YES.. 5

AD3RA4/B
ADICD5
ALCFGNA4

B. How many binges like that have you had?
IF 3 OR MORE, MARK TALLY SHEETS A AND C AND SKIP TO C. IF DK, ASK B1. OTHERS SKIP TO C.

___ ___ TIMES A,C

AD3RA4/B
ADICD5
ALCFGNA4

1. Did you go on binges 3 or more times?

NO.... 1
YES 5 A,C

C. How old were you the (first/last) time (you binged and neglected your responsibilities)?

AGE ONS: ____/____ 1 2 3 4 5
ONS:
AGE REC: ____/____ 1 2 3 4 5
REC:

AA4A1

IF FEWER THAN 3 BINGES, CODE "NO" SILENTLY.

D. Did this happen 3 or more times in any 12-month period? NO1

YES 5

---

(31)
ALCFGNA3

E17 Have you ever had blackouts, that is when you did not pass out while drinking, but you drank enough so that the next day you could not remember things you had said or done?

NO (SKIP TO E18) 1
YES .. 5

A. How old were you the (first/last) time?

AGE ONS: ____/____
1 2 3 4 5

AGE REC: ____/____
1 2 3 4 5

B. How many blackouts have you had from drinking? ___ __ TIMES
IF DK, ASK B1. OTHERS SKIP TO E18.

1. Did you have 3 or more blackouts? NO1
YES5

---

(10)

E18 Did you ever need a drink just after you had gotten up (that is, before breakfast)?

NO 1
YES . 5

ALCFGNB3

A. Did you ever take a drink just after you had gotten up? NO1
YES5

IF E18 AND E18A ARE BOTH CODED 1, SKIP TO E19. OTHERS CONTINUE.

B. How old were you the (first/last) time you took (needed) a drink just after you had gotten up?

AGE ONS: ____/____
1 2 3 4 5

AGE REC: ____/____
1 2 3 4 5

C. Did this happen 3 or more times?

NO.. 1
YES 5

---

(11)
ADICD1

E19 In situations where you couldn't drink, did you ever have such a strong desire for it that you couldn't think of anything else?

NO . . . (SKIP TO E20) 1
YES 5C

A. How old were you the (first/last) time?

AGE ONS: ____/____
1 2 3 4 5

AGE REC: ____/____
1 2 3 4 5

(19) ALCFGNB4	E20 Did you ever drink unusual things such as rubbing alcohol, mouthwash, vanilla extract, cough syrup, or any other non-beverage alcohol?	NO<br>YES ..	(SKIP TO E21) 1<br>5
	A. How old were you the first time?	AGE ONS:	____/____<br>1 2 3 4 5
(36) AD3RA4/B AA3RA2 AA4A2	E21 Have you used alcohol 3 or more times while taking medications or drugs you knew were dangerous to mix with alcohol? **PROBE FOR AMOUNTS OF ALC/DRUGS AND REASON THOUGHT DANGEROUS.**<br><br>**SPECIFY:** ______________________________________<br>____	NO<br>YES	. . . (SKIP TO E22) 1<br>. . . . (SPECIFY) 5 A
	A. What medication(s) or drug(s)<br>____<br>____	CODE:<br>CODE:	
	B. How old were you the (first/last) time you mixed alcohol and drugs when you knew it was dangerous?	AGE ONS:<br><br>AGE REC:	____/____<br>1 2 3 4 5<br><br>____/____<br>1 2 3 4 5
AA4A2	C. Did this happen 3 or more times in any 12-month period?	NO..1<br>YES 5	
ADICD6	D. Did you have any harmful effects from mixing alcohol and (DRUG)?<br><br>**SPECIFY:**<br>_________________________________<br>____	NO<br>YES	1<br>. . . . .(SPECIFY) 5C
(30)	E22 When you were drunk, did you ever drive a car, motorcycle or boat; use a knife, power equipment or gun; cross against traffic; climb or swim; or put yourself in any other situation where you might have gotten hurt?	NO<br>YES ..	(SKIP TO E23) 1<br>5
	A. How old were you the (first/last) time?	AGE ONS:	____/____<br>1 2 3 4 5

AGE REC: ____/____
1 2 3 4 5

AD3RA4/B
AA3RA2/B
ASP3RC7
ASP4A5

B. How many times has this happened? **IF 3 OR MORE, MARK TALLY A AND SKIP TO C. IF FEWER THAN 3, SKIP TO E23. IF DK, ASK B1.** ___ __ TIMES A

AD3RA4/B
AA3RA2/B
ASP3RC7
ASP4A5

1. Did this happen 3 or more times?

NO . . . .(SKIP TO E23) 1
YES .. 5 A

AA4A2

C. Did this happen 3 or more times in any 12-month period?

NO 1
YES .. 5

---

(27)
ALCFGNC2

E23 Have you ever been arrested for drunk driving?

NO . . . .(SKIP TO E24) 1
YES .. 5

A. How old were you the (first/last) time?

AGE ONS: ____/____
1 2 3 4 5

AGE REC: ____/____
1 2 3 4 5

AD3RA4/B
AA3RA2/B
ASP3RC7
ASP4A5

B. How many times has this happened? **IF 3 OR MORE, MARK TALLY A AND SKIP TO C. IF FEWER THAN 3, SKIP TO E24. IF DK, ASK B1.** ___ __ TIMES A

AD3RA4/B
AA3RA2/B
ASP3RC7
ASP4A5

1. Did this happen 3 or more times?

NO . . . .(SKIP TO E24) 1
YES 5 A

AA4A3

C. Did this happen 3 or more times in any 12-month period?

NO1
YES 5

---

(27)
ALCFGNC2

E24 Has your drinking and driving ever resulted in your damaging your car or having an accident?
COUNT ALL ACCIDENTS, EVEN IF NOT REPORTED TO THE POLICE.

NO (SKIP TO E25) 1
YES .. 5

ASP3RC7

A. How old were you the (first/last) time?

AGE ONS: ____/____
1 2 3 4 5

AGE REC: ____/____ 1 2 3 4 5

AD3RA4/B
AA3RA2/B

B. How many times has this happened? **IF 3 OR MORE, MARK TALLY A AND SKIP TO C. IF FEWER THAN 3, SKIP TO E25. IF DK, ASK B1.** ___ __ TIMES A

AD3RA4/B
AA3RA2/B

1. Did this happen 3 or more times?

NO . . . .(SKIP TO E25) 1
YES.. 5A

AA4A2

C. Did this happen 3 or more times in any 12-month period?

NO.. 1

YES 5

---

(23)
AD3RA4/B

E25 Has your drinking or being drunk or hung over <u>often</u> interfered with your work, school, household, or child care responsibilities?

NO (SKIP TO E26) 1
YES. . . . .(SPECIFY) 5 A

SPECIFY: ____________________________________

____

A. How old were you the (first/last) time?

AGE ONS: ____/____ 1 2 3 4 5

AGE REC: ____/____ 1 2 3 4 5

AA4A1

B. Did this happen 3 or more times in any 12-month period?

NO.. 1

YES 5

FOR EACH 5 CODED IN E26A.1-8, GET AGE ONSET AND ASK, "Did this happen 3 or more times?" **CODE IN COL II.**

			COL I NO	COL I YES	AGE ONS	COL II NO	COL II YES
(17) ALCFGND2 ALCFGND4 AD3RA6 AA3RA1/B	E26A	1. Did your drinking ever result in objections from or problems with your family, friends, doctors, clergy, or people at work or school?	1	5	___/___	1	5A
ALCFGND3 AD3RA6		2. Have you ever lost friends on account of your drinking?	1	5	___/___	1	5 A
ALCFGNC3 AD3RA6		3. Did your drinking ever cause you to have problems at					

AA3RA1/B	work or school?	1	5	___/___	1	5 A	
	4. Did you ever get into arguments when you had been drinking?	1	5	___/___	1	5	
ALCFGNC4	5. Did you ever hit things or throw something when you had been drinking?	1	5	___/___	1	5	
ALCFGNC4	6. Did you ever hit a significant other or anyone in your family when you had been drinking?	1	5	___/___	1	5	
ALCFGNC4	7. Did you ever hit anyone else when you had been drinking without getting into a fight?	1	5	___/___	1	5	
ALCFGNC4	8. Did you ever get into physical fights while drinking?	1	5	___/___	1	5	

IF ANY 5 IS CODED IN COL. I, CONTINUE. OTHERS SKIP TO E27.

B. How old were the last time any of these happened **(REVIEW SX CODED 5 IN COL. I)**?

AGE REC: ____/____ 1 2 3 4 5

AA4A4

C. Did any of these experiences happen 3 or more separate times in any 12-month period?

NO.. 1

YES 5

---

(24) E27 Did your drinking cause serious or repeated problems in any marriage or love relationship?

NO . . . . (SKIP TO E28) 1

YES .. 5

A. How old were you the (first/last) time?

AGE ONS: ____/____ 1 2 3 4 5

AGE REC ____/____ 1 2 3 4 5

AA4A4

B. Did this happen 3 or more times in any 12-month period?

NO.. 1

YES 5

AD3RA6

C. Did you continue to drink knowing it caused these problems?

NO.. 1 A

YES 5

---

(28)
ALCFGNC1 E28 Have you ever been arrested or detained by the police even for a few hours because of drunk behavior (other than for drunk driving)?

NO . . . .(SKIP TO E29) 1

YES .. 5

A. How old were you the (first/last) time? | AGE ONS: ____/____ 1 2 3 4 5

AGE REC: ____/____ 1 2 3 4 5

AD3RA6 B. How many times has this happened? **IF 3 OR MORE, MARK TALLY A AND SKIP TO C. IF FEWER THAN 3, SKIP TO E29. IF DK, ASK B1.** ___ ___ TIMES A

AD3RA6 1. Did this happen 3 or more times?

NO . . . (SKIP TO E29) 1
YES .. 5A

AA4A3 C. Did this happen 3 or more times in any 12-month period?

NO.. 1

YES 5

---

(29) E29 Have you ever accidentally injured yourself when you were drinking; that is, had a bad fall or cut yourself badly, been hurt in a traffic accident, or anything like that?

NO . . . (SKIP TO E31) 1
YES .. 5

A. How old were you the (first/last) time?

AGE ONS: ____/____ 1 2 3 4 5

AGE REC: ____/____ 1 2 3 4 5

AD3RA4/B
ADICD6
AA3RA2
B. How many times has this happened? **IF 3 OR MORE, MARK TALLY A AND C, AND SKIP TO C. IF FEWER THAN 3, SKIP TO E31. IF DK, ASK B1.** ___ ___ TIMES A,C

AD3RA4/B
ADICD6
AA3RA2
1. Did this happen 3 or more times?

NO . . . (SKIP TO E31) 1
YES .. 5 A,C

AA4A2 C. Did this happen 3 or more times in any 12-month period?

NO.. 1

YES 5

---

(16) E30 OMITTED.

---

(35)
ALCFGNA2
E31 There are several health problems that can result from long stretches of drinking. Did drinking ever cause you to have...

	NO	YES
1. liver disease or yellow jaundice?	1	5
2. stomach disease or make you vomit blood?	1	5

3.pancreatitis?	1	5	
4.damage to your heart (cardiomyopathy)?	1	5	
5.your feet tingle or feel numb for many hours?	1	5	
6. memory problems even when you weren't drinking (so, not counting blackouts)?	1	5	
7.any other physical health problems? **IF YES, SPECIFY.**	1	5	

SPECIFY:___________________________________

IF ALL CODED 1, SKIP TO E32. OTHERS CONTINUE.

A. How old were you when you first found out that drinking had given you any of these health problems?

AGE ONS: ____/____ 1 2 3 4 5

AD3RA6
AD47
ADICD6
AA3RA1

B. Did you continue to drink knowing that drinking caused you to have health problems?

NO.. 1
YES 5 A,B,C

(36)
AD3RA6
AD47
ADICD6
AA3RA1

E32 Have you ever continued to drink when you knew you had any (other) serious physical illness or condition that might be made worse by drinking?

NO (SKIP TO E33) 1
YES .. 5 A,B,C

A.What illness or condition?

CODE: _____________

____

____

____

B. How old were you the (first/last) time?

AGE ONS: ____/____ 1 2 3 4 5

AGE REC: ____/____ 1 2 3 4 5

C. Did drinking make your illness or condition worse?

NO.. 1

YES 5

---

(37)

E33 Has drinking ever caused you emotional or psychological problems like:

	NO	YES
1. feeling depressed or uninterested in things for more than 24 hours to the point that it interfered with your functioning?	1	5
2. feeling jumpy or easily startled or nervous for more than 24 hours to the point that it interfered with your functioning?	1	5
3. having such trouble thinking clearly for more than 24 hours that it interfered with your functioning?	1	5
4. feeling paranoid or suspicious of people for more than 24 hours to the point that it interfered with your relationships?	1	5
5. hearing, seeing, or smelling things that weren't really there?	1	5

IF ALL ARE CODED 1, SKIP TO E34. OTHERS CONTINUE.

AD3RA6
AD47
ADICD6
AA3RA1

A. Did you continue to drink after you knew it caused you any of these problems?

NO (SKIP TO E34) 1
YES.. 5 A,B,C

B. How old were you the (first/last) time?

AGE ONS: ____/____
1 2 3 4 5

AGE REC: ____/____
1 2 3 4 5

---

(25)
ALCFGND1

E34 Did you ever think that you were an excessive drinker or think that you drank too much for your own good?

NO SKIP TO E35) 1

YES.. 5

A. How old were you the first time you thought that?

AGE ONS: ____/____
1 2 3 4 5

---

(26)
ALCFGND5

E35 Have you ever felt guilty about drinking?

NO . . (SKIP TO BOX E36) 1
YES.. 5

A. How old were you the first time?

AGE ONS: ____/____
1 2 3 4 5

(18) E36 OMITTED.

BOX E36 CHECK TALLY A, B, AND C. IF NO MARKS, SKIP TO F1, p. 57. OTHERS CONTINUE.

---

(32)
ALCFGNA1

E37 People who cut down, stop, or go without drinking after drinking steadily for some time may not feel well. These feelings are more intense and can last longer than the usual hangover.

When you stopped, cut down or went without drinking, did you ever experience any of the following problems for most of the day for 2 days or longer? **REPEAT STEM OFTEN. CODE IN COL. I. (NO=1, YES=5)**

II-AD3R
III-AD4
IV-AICD

	I	II (DSM3R)	III (DSM4)	IV (ICD)
1.Did you have the shakes (hands trembling)?	1 5	1 5	1 5	1 5
2.Were you unable to sleep?	1 5	1 5	1 5	1 5
3.Did you feel anxious?	1 5	1 5	1 5	
4.Did you feel depressed or irritable?	1 5	1 5		
5.Did your heart beat fast or did you sweat?	1 5	1 5	1 5	1 5
6.Did you have nausea or vomiting?	1 5	1 5	1 5	1 5
7.Did you feel physically weak?	1 5	1 5		1 5
8.Did you have headaches?	1 5	1 5		1 5
9.Did you see or hear things that weren't there?	1 5	1 5	1 5	1 5
10.Were you fidgety or restless?	1 5		1 5	1 5

BOX E37 IF NO 5'S CODED IN COLUMN I, SKIP TO E38. IF R HAD SHAKES (E37.1= 5), ASK A. IF NO SHAKES (E37.1=1), SKIP TO B.

A. How old were you the (first/last) time you had the shakes (hands trembling)?

AGE ONS: ____/____
1 2 3 4 5

AGE REC: ____/____
1 2 3 4 5

AD3RB

B. What was the longest time that (this/any of these) problem(s) lasted? ___ __ DAYS

IF ONLY ONE SX IS CODED 5 IN E37.1-10, SKIP TO H. OTHERS CONTINUE.

C. Was there ever a time when two or more of these problems occurred together?

NO . . . . (SKIP TO F) 1

YES 5

AD3RA8
AD42A
ADICD3

D. Which ones? **CODE IN COL. II, III, IV. (NO=1, YES=5)**

IF SHAKES IN COL. II AND 1+ SX IN COL. II, MARK TALLY A. A
IF 2+ SX IN COL. III, MARK TALLY B. B
IF 3+ SX IN COL IV, MARK TALLY C. C

E. How old were you the (first/last) time these problems occurred together?

AGE ONS: ____/____ 1 2 3 4 5

AGE REC: ____/____ 1 2 3 4 5

AD3RB

F. How many times did you have problems like these (occur together)? **IF DK, ASK F1. OTHERS SKIP TO G.** ___ __ TIMES

AD3RB

1. Did this occur 3 or more times?

NO.. 1

YES 5

IF NO 5'S IN COL. III, SKIP TO H.
OTHERS CONTINUE.

G. You said you (**REVIEW ALL 5'S CODED IN COL. III**). Did (this/these) problem(s) interfere with your functioning at work, school, or home?

NO.. 1

YES 5

H. Have you ever taken a drink to keep from having <u>any</u> of these problems (or to make them go away) (**REVIEW ALL 5'S CODED IN COL. I**)?

NO (SKIP TO J) 1

YES.. 5

1. How old were you the (first/last) time?

AGE ONS: ____/____ 1 2 3 4 5

AGE REC: ____/____ 1 2 3 4 5

AD3RA9/B
AD42B
ADICD3

I. Did this happen 3 or more times?

NO.. 1

YES 5 A,B,C

J. Did you ever take any medication or drug to avoid any of these problems (or to make them go away)?
DO NOT COUNT ASPIRIN, TYLENOL, ETC. <u>DO</u> COUNT MEDS GIVEN IN TREATMENT.

NO. 1

YES . . . . (SPECIFY) 5

		**SPECIFY:**______________________________ ____	CODE ___ ___ ___<br>CODE: ___ ___ ___
(33)<br>AD3RA8<br>AD42A<br>ADICD3<br>ALCFGNAI	E38	When you stopped, cut down, or went without drinking, did you ever have fits, seizures, or convulsions, where you lost consciousness, fell to the floor, and had difficulty remembering what happened?	NO SKIP TO E39) 1<br>YES 5 A,B,C
	A.	How old were you the (first/last) time this happened?	AGE ONS: ____/____ 1 2 3 4 5<br>AGE REC: ____/____ 1 2 3 4 5
_____<br>AD3RB	B.	How many times did this happen? **IF DK, ASK B1. OTHERS SKIP TO C.**	___ __ TIMES
		1. Did this occur 3 or more times?	NO.. 1<br>YES 5
AD3RA9/B<br>AD42B<br>ADICD3	C.	On 3 or more different occasions have you taken a drink to keep from having fits, seizures, or convulsions or to make them go away?	NO (SKIP TO D) 1<br>YES .. 5 A,B,C
		1. How old were you the (first/last) time this happened?	AGE ONS: ____/____ 1 2 3 4 5<br>AGE REC: ____/____ 1 2 3 4 5
	D.	Did you ever take any medication or drug to avoid having fits, seizures, or convulsions (that occurred because you went without drinking) or to make them go away?<br><br>**DO NOT COUNT ASPIRIN, TYLENOL, ETC. <u>DO</u> COUNT MEDS GIVEN IN TREATMENT.**<br><br>**SPECIFY**:________________________ ____	NO.. 1<br>YES . . (SPECIFY) 5<br><br>CODE:<br>CODE:
(34)<br>AD3RA8<br>AD42A	E39	When you stopped, cut down, or went without drinking, did you ever have the DT's, that is, where you were very	NO (SKIP TO E40) 1

ADICD3<br>ALCFGNA1		confused, extremely shaky, felt very frightened or nervous, or saw things that weren't really there?	YES ..	5 A,B,C
	A.	How old were you the (first/last) time this happened?	AGE ONS:<br><br>AGE REC:	____/____<br>1 2 3 4 5<br><br>____/____<br>1 2 3 4 5
AD3RB	B.	How many times did this happen? **IF DK, ASK B1. OTHERS SKIP TO C.**	___ __	TIMES
		1. Did this occur 3 or more times?	NO<br>YES	1<br>5
AD3RA9/B<br>AD42B<br>ADICD3	C.	On 3 or more different occasions have you taken a drink to keep from having the DT's or to make them go away?	NO<br>YES ..	(SKIP TO D) 1<br>5 A,B,C
		1. How old were you the (first/last) time this happened?	AGE ONS:<br><br>AGE REC:	____/____<br>1 2 3 4 5<br><br>____/____<br>1 2 3 4 5
	D.	Did you ever take any medication or drug to avoid the DT's or to make them go away?<br>**DO NOT COUNT ASPIRIN, TYLENOL, ETC. <u>DO</u> COUNT MEDS GIVEN IN TREATMENT.**<br><br>**SPECIFY**:________________________<br>____	NO .. 1<br><br>YES<br><br>CODE:<br>CODE:	. . . . (SPECIFY) 5<br><br><br>____________________

DSMIIIR	**BOX E40**	**IF 3 OR MORE BOXES MARKED ON TALLY SHEET A, CONTINUE. OTHERS SKIP TO BOX E41.**	

HAND R ALCOHOL TALLY A.

(40)<br>AD3RB	E40	A. I have checked on this sheet the experiences with alcohol that you told me about. The experiences are grouped into boxes. You told me **(REVIEW SX)**. I'd like you to tell me whether there has ever been a period lasting a month or longer when you had experiences from 3 or more boxes occurring together? **IF YES:**	NO .. 1<br><br>YES	..(SKIP TO C) 5

Please tell me the box and number for each experience. **CIRCLE SYMPTOMS THAT CLUSTER. MUST BE FROM 3 DIFFERENT BOXES. <u>DO NOT</u> COUNT SYMPTOMS THAT OCCURRED AS A RESULT OF AN ISOLATED INCIDENT.**

B. Was there ever a period lasting a month or longer when you had experiences from 2 boxes occurring together? **IF YES:** Please tell me the box and number for each experience. **CIRCLE SYMPTOMS THAT CLUSTER. MUST BE FROM 2 DIFFERENT BOXES. <u>DO NOT</u> COUNT SYMPTOMS THAT OCCURRED AS A RESULT OF AN ISOLATED INCIDENT.**

NO . (SKIP TO BOX E41) 1
YES 5

C. How old were you the (first/last) time you had experiences from 3(2) boxes occur within a period lasting a month or more?

t
AGE ONS: ____/____
1 2 3 4 5

t
AGE REC: ____/____
1 2 3 4 5

DSMIV

BOX E41 IF 3 OR MORE BOXES MARKED ON TALLY SHEET B, CONTINUE. OTHERS SKIP TO BOX E42.

HAND R ALCOHOL TALLY B.

E41 A. I have checked the experiences with alcohol that you told me about. The experiences are grouped into boxes <u>different</u> from the one I just showed you. You told me **(REVIEW SX).** I'd like you to tell me whether there has ever been a 12-month period in which you had experiences from 3 or more boxes? **IF YES:** Please tell me the box and number for each experience that occurred during the same 12-month period, even if the problems did not last the full 12 months. **CIRCLE SYMPTOMS THAT CLUSTER. MUST BE FROM 3 DIFFERENT BOXES. <u>DO NOT</u> COUNT SYMPTOMS THAT OCCURRED AS A RESULT OF AN ISOLATED INCIDENT.**

NO (SKIP TO BOX E42) 1
YES 5

B. How old were you the (first/last) time you had experiences from 3 or more boxes occur within a 12-month period?

AGE ONS: ____/____
AGE REC: ____/____

(41)
ICD10

BOX E42 IF 3 OR MORE BOXES MARKED ON TALLY SHEET C, CONTINUE. OTHERS SKIP TO BOX E43.

HAND R ALCOHOL TALLY C.

E42 A. This is (another) list of experiences grouped into boxes that are different from the ones you have already seen. You told me **(REVIEW SX).** I'd like you to tell me whether there has ever been a period lasting a month or longer when you had experiences from 3 boxes occurring together? **IF YES:** Which ones? **CIRCLE SX THAT CLUSTER. MUST BE FROM 3 DIFFERENT BOXES. DO NOT COUNT SYMPTOMS THAT OCCURRED AS A RESULT OF AN ISOLATED INCIDENT.**

NO.. 1 . . . . (SKIP TO C)

YES 5

B. Have experiences from 3 boxes ever occurred together repeatedly within a 12-month period?
IF YES: Which ones? **CIRCLE SX THAT CLUSTER. MUST BE FROM 3 DIFFERENT BOXES. DO NOT COUNT SYMPTOMS THAT OCCURRED AS A RESULT OF AN ISOLATED INCIDENT.**

NO 1 (SKIP TO BOX E43)

YES 5

C. How old were you the (first/last) time?

AGE ONS: ____/____

AGE REC: ____/____

BOX E43 IF 2+ BOXES MARKED ON TALLY SHEET A, CONTINUE. OTHERS SKIP TO E44.

(8) E43 (Since (AGE OF REGULAR DRINKING IN E5)), what is the longest period of time you have gone without drinking? ___ __ MONTHS

IF LESS THAN 3 MONTHS, SKIP TO E44.

A. How many times have you gone without drinking for 3 months or longer? ___ __ TIMES

B. Can you tell me when these periods occurred?
IF MORE THAN 4 ABSTINENT PERIODS, RECORD THE 4 LONGEST.

PERIOD 1: FROM __ __/__ __ __ __ TO __ __/__ __ __ __ ***t***
MO YEAR MO YEAR

PERIOD 2: FROM __ __/__ __ __ __ TO __ __/__ __ __ __ ***t***
MO YEAR MO YEAR

PERIOD 3: FROM __ __/__ __ __ __ TO __ __/__ __ __ __ ***t***
MO YEAR MO YEAR

PERIOD 4: FROM __ __/__ __ __ __ TO __ __/__ __ __ __ *t*

MO YEAR MO YEAR

(38) E44 Have you ever brought up any problem you might have had with drinking with any professional?

NO . . . .(SKIP TO E45) 1

YES 5

A. Did you talk with:

	NO	YES
1.a psychiatrist?	1	5
2.another medical doctor?	1	5
3.a psychologist?	1	5
4.another mental health professional?	1	5
5.a member of the clergy?	1	5
6.another professional? **(IF YES, SPECIFY)**	1	5

SPECIFY:__________________

B. How old were you the (first/last) time you brought up any problem you had with drinking?

AGE ONS: ____/____
1 2 3 4 5

AGE REC: ____/____
1 2 3 4 5

C. With whom did you speak first?
RECORD CODE (1-6)

CODE: _____

REFER TO B9 BEFORE ASKING

(39) E45 Have you ever been treated for a drinking problem?

NO (SKIP TO D) 1

YES 5

A. Were you treated:

	NO	YES
1.at AA or another self-help group?	1	5
2.at an outpatient alcohol program?	1	5
3. at an outpatient program for something other than alcohol?	1	5
4.at an inpatient alcohol program?	1	5

5. when you were an inpatient for medical complications due to alcohol? 1 5

6.at any other place or program? . (**IF YES, SPECIFY**) 1 5

SPECIFY:

____

B. How old were you the (first/last) time you were treated?

AGE ONS: ____/____ 1 2 3 4 5

AGE REC: ____/____ 1 2 3 4 5

C. Where were you first treated? **RECORD CODE (1-6) AND THEN SKIP TO F1, p. 46.**

CODE: ______

D. Did you ever attend a self-help group (like AA) for your drinking?

NO (SKIP TO F1, p. 57) 1

YES 5

1. How old were you the (first/last) time you attended a self-help group for your drinking?

AGE ONS: ____/____ 1 2 3 4 5

AGE REC: ____/____ 1 2 3 4 5

(1) F1 Have you ever used marijuana or — NO. . . . (SKIP TO G1, p. 68) 1; YES 5

A. How many times? — ___ ___ ___ ___ TIMES

IF FEWER THAN 21 TIMES, CODE B "NO" SILENTLY. OTHERS CONTINUE.

B. Did you ever use marijuana at least 21 times in a single year? — NO ....1; YES 5

---

(2) F2 How old were you the first time you used marijuana? — AGE ONS: ___/___ 1 2 3 4 5 *t*

IF AGE ONS 15 OR LATER, SKIP TO B. OTHERS CONTINUE.

A. Did you use marijuana more than once before you were 15? — NO ....1; YES 5

B. How old were you the last time you used marijuana? — AGE REC: ___/___ 1 2 3 4 5 *t*
IF REC CODE=5, SKIP TO D. OTHERS CONTINUE.

1. Did you use marijuana at least 21 times during the past 12 months? — NO ....1; YES(SKIP TO F3) 5

D. Did you ever use marijuana at least once a week for a month or more? — NO ....1; YES 5

BOX F2 IF F1B IS CODED 1, SKIP TO G1, p. 68. OTHERS CONTINUE.

(3) DRFGNC F3 What was the longest period of time you used marijuana almost every day? — ___ ___ ___ ___ UNITS

CODE UNITS:
DAYS 1
WEEKS 2
MONTHS 3
YEARS 4

IF NEVER, CODE 0000 DAYS AND SKIP TO B.
IF LESS THAN 2 WEEKS, SKIP TO B.
OTHERS CONTINUE.

A. How old were you the (first/last) time you used marijuana almost every day for at least two weeks?

AGE ONS: ___/___ *t*
1 2 3 4 5 *t*

AGE REC: ___/___
1 2 3 4 5

B. Please think about the period when you were using marijuana the most. During that period, how many days per month did you use marijuana?

___ ___ DAYS

C. During that period of heaviest use, how much marijuana did you use on an average day?

___ ___ UNITS
CODE UNITS:
HITS..2
JOINTS/CIGS 3
PIPEFULS 4

D. How old were you when that period started?

AGE: __ __ *t*

E. How long did that period last?

__ __ __ MONTHS

(4)
DRFGNC

F4 Have you ever stayed high from marijuana for a whole day or more?

NO(SKIP TO F5) 1
YES 5

A. How old were you the (first/last) time you stayed high from marijuana for a whole day or more?

AGE ONS: ___/___
1 2 3 4 5

AGE REC: ___/___
1 2 3 4 5

BEGIN SCORING MARIJUANA TALLY SHEETS A,B,C.

(5)
DD3RA3/B
DD45
DDICD5

F5 Has there ever been a period of a month or more when a great deal of your time was spent using marijuana, getting marijuana, or getting over its effects?

NO 1
YES 5

A,B, C

---

(6)

			COL. I		COL. II		
	F6	Because of your marijuana use, did you ever experience any of the following: **CODE IN COLUMN I.**	NO	YES	NO	YES	
		1. Feeling depressed or uninterested in things for more than 24 hours to the point that it interfered with your functioning?	1	5	1	5	A,B, C
		2. Having trouble concentrating or having such trouble thinking clearly for more than 24 hours that it interfered with your functioning?	1	5	1	5	A,B, C
		3. Feeling paranoid or suspicious of people for more than 24 hours to the point that it interfered with your relationships?	1	5	1	5	A,B, C
		4. Decreased contact with friends or family?	1	5	1	5	A
		5. Hearing, seeing, or smelling things that weren't really there?	1	5	1	5	A,B, C
		**FOR EACH 5 CODED IN COL.I, ASK F6A.**					
DD3RA6/B DD47 DDICD6 DA3RA1/B		A. Did you continue to use marijuana after you knew it caused this? **CODE IN COLUMN II.**					
DA4A4		**IF F6.4 IS CODED 1, SKIP TO F7. OTHERS CONTINUE.**	NO ....1<br>YES 5				
		B. Did you have decreased contact with friends or family 3 or more times in any 12-month period?					

---

(7) DD3RA2 DD44 DDICD2	F7	Have you <u>often</u> wanted to stop or cut down on marijuana?	NO ....1<br>YES 5	A,B, C

DD3RA2		A. Have you ever tried to stop or cut down on marijuana but found you couldn't? **IF NEVER TRIED TO STOP/CUT DOWN, CODE NO.**	NO, COULD STOP 1<br>YES, COULD NOT STOP 5	A

IF NO, COULD STOP (OR NEVER TRIED), SKIP TO F8. OTHERS CONTINUE.

DD44<br>DDICD2		B. Were you unable to stop or cut down 3 or more times?	NO ....1<br>YES 5	B,C
(8)<br>DD3RA1/B<br>DD43<br>DDICD2	F8	Have you <u>often</u> used marijuana more frequently or in larger amounts than you intended to?	NO ....1<br>YES 5	A,B,<br>C
(9)<br>DD3RA7<br>DD41<br>DDICD4	F9	Did you ever need larger amounts of marijuana to get an effect, or did you ever find that you could no longer get high on the amount you used to use?	NO ....1<br>YES 5	A,B,<br>C

(10) F10 When you stopped, cut down, or went without marijuana, did you ever experience any of these following problems <u>for most of the day for 2 days or longer</u>? Did you..... **CODE IN COLUMN I.**

	COL. I NO	COL. I YES	COL. II NO	COL. II YES
1. feel nervous, tense, restless or irritable?	1	5	1	5
2. have trouble sleeping?	1	5	1	5
3. tremble or twitch?	1	5	1	5
4. sweat or have a fever?	1	5	1	5
5. have nausea or vomiting?	1	5	1	5
6. have diarrhea or stomach aches?	1	5	1	5
7. have a marked increase or decrease in appetite, that is, have a significant change from your <u>normal</u> level?	1	5	1	5

BOX F10A IF NO 5'S CODED IN F10.1-7, SKIP TO F11. OTHERS CONTINUE.

		A. Have you ever used marijuana to keep from having any of these problems (or to make them go away)?	NO. . . (SKIP TO BOX F10B) 1<br>YES 5	
DD3RA9/B<br>DD42B<br>DDICD3		B. Did this happen 3 or more times?	NO ....1<br>YES 5	A,B, C

BOX F10B IF ONLY ONE 5 CODED IN COL. I, SKIP TO F11. OTHERS CONTINUE.

DD3RA8<br>DD42A<br>DDICD3<br>DRFGNA		C. Did these problems ever occur together?	NO. . . . . .(SKIP TO G) 1<br>YES 5	A,B, C
		D. Which ones? **CODE IN COL. II**		
DD3RB<br>DA3RA		E. How many times did you have problems like that (when they occurred together)?	___ ___ TIMES	
DD3RB		F. What was the longest time these problems occurred together?	___ ___ DAYS	
		G. Did these problems interfere with your functioning at work, school, or home?	NO ....1<br>YES 5	
(11)<br>ASP3RC7<br>ASP4A5	F11	Have you ever been under the effects of marijuana when it increased your chances of getting hurt, for instance, when driving a car or boat, using knives, machinery or guns, crossing against traffic, climbing or swimming?	NO(SKIP TO B) 1<br>YES 5	
DD3RA4/B<br>DA3RA2/B		A. Have you been in situations like this 3 or more times?	NO. . . . . (SKIP TO B) 1<br>YES 5	A
DA4A2		1. Did this happen 3 or more times in any 12-month period?	NO ....1<br>YES 5	
		B. Did marijuana ever cause you to have any accidental injuries like a bad fall, cutting or burning yourself, or being hurt in a	NO(SKIP TO F12) 1<br>YES 5	

		traffic accident?		
DD3RA6/B<br>DDICD6<br>DA3RA1/B		C. Did this happen 3 or more times?	NO. . . . (SKIP TO F12) 1<br>YES 5	A,C
DA4A2		1. Did this happen 3 or more times in any 12-month period?	NO ....1<br>YES 5	
(12)	F12	Did your marijuana use ever cause you to have problems with your friends or family?	NO(SKIP TO F13) 1<br>YES 5	
DA4A4		A. Did this happen 3 or more times in any 12-month period?	NO ....1<br>YES 5	
DD3RA6<br>DA3RA1		B. Did you continue to use marijuana after you realized it was causing these problems?	NO ....1<br>YES 5	A
	F13	Have you ever been arrested or had any other trouble with the police because of your marijuana use?<br>**SPECIFY:**_____________________<br>____	NO . . . . .(SKIP TO F14) 1<br>YES. . . . . .(SPECIFY) 5	
DD3RA6		A. Did this happen 3 or more times?	NO . . . . (SKIP TO F14) 1<br>YES. 5	A
DA4A3		1. Did this happen 3 or more times in any 12-month period?	NO ....1<br>YES 5	
(14)<br>DD3RA4/B	F14	Has your being high on marijuana or experiencing its after-effects often interfered with your work, school, household, or child care responsibilities?	NO. . . . .(SKIP TO F16) 1<br>YES 5	A
DA4A1		A. Did this happen 3 or more times in any 12-month period?	NO ....1<br>YES 5	
(16)	F15	OMITTED.		
(15)<br>DDICD1	F16	In situations where you couldn't use marijuana, did you ever have such a strong desire for it that you couldn't think of anything else?	NO. . . . . (SKIP TO F17) 1<br>YES 5	C
		A. How old were you the (first/last)	AGE ONS: ___/___	

		time?	ONS: 1 2 3 4 5<br>AGE REC: ___/___<br>REC:1 2 3 4 5
(13)	F17	Have you given up or greatly reduced important activities like sports, work, or associating with friends or relatives while using marijuana?	NO. . . .(SKIP TO F18) 1<br>YES 5
DD3RA5/B<br>DD46<br>DDICD5		A. Has this happened 3 or more times, or did it last a month or longer?	NO ....1<br>YES ....5<br>A,B, C
(17)	F18	Have you ever used marijuana together with one or more other drugs, including alcohol?<br><br>**IF YES:** Which ones?<br><br>1. ____________________________<br>2. ____________________________<br>3. ____________________________<br>4. ____________________________	NO. . . .(SKIP TO BOX F19) 1<br>ALCOHOL ONLY 3<br>YES(SPECIFY)5<br><br>CODE: ___ ___ ___<br>CODE: ___ ___ ___<br>CODE: ___ ___ ___<br>CODE: ___ ___ ___
DSMIIIR		**BOX F19 IF ONE OR MORE BOXES MARKED ON TALLY SHEET A, CONTINUE. OTHERS SKIP TO BOX F20.**	
(19)	F19	**HAND R MARIJUANA TALLY A.**<br>I have checked on this sheet the experiences with marijuana that you have told me about. You told me **(REVIEW SX)**. When was the (first/last) time that you had any of these experiences?	AGE ONS: ___/___<br>ONS: 1 2 3 4 5<br>AGE REC: ___/___<br>REC:1 2 3 4 5
		**BOX F19A IF 3 OR MORE BOXES MARKED ON TALLY A, CONTINUE. OTHERS SKIP TO BOX F20. NOTE: DO NOT COUNT SYMPTOMS THAT OCCURRED AS A**	

		**RESULT OF AN ISOLATED INCIDENT.**	
DD3RB		A. Thinking about these experiences with marijuana, was there ever a period lasting a month or longer when you had experiences from 3 or more different boxes occurring together? **IF YES:** Please tell me the box and number of those experiences. **CIRCLE SYMPTOMS THAT CLUSTER. NOTE: MUST BE 3 FROM DIFFERENT BOXES.**	NO ....1<br>YES. . . . .(SKIP TO C) 5<br><br>NO. . . .(SKIP TO BOX F20) 1<br>YES 5
		B. Was there ever a period lasting a month or longer when you had experiences from 2 boxes occurring together? **IF YES:** Which ones? **CIRCLE SX. MUST BE FROM 2 DIFFERENT BOXES.**	AGE ONS: ___/___ ***t***<br>ONS: 1 2 3 4 5<br><br>AGE REC: ___/___ ***t***<br>REC:1 2 3 4 5
		C. How old were you the (first/last) time you had experiences from 3(2) boxes occur within a period of a month or more?	

DSM-IV	**BOX F20**	**IF 3 OR MORE BOXES MARKED ON TALLY SHEET B, CONTINUE. OTHERS SKIP TO BOX F21.**	
		**HAND R MARIJUANA TALLY B.**	
DD4	F20	A. Please review this list of experiences which are grouped into boxes that are different from the last one I showed you. You told me **(REVIEW SX)**. Was there ever a 12-month period in which you had experiences from 3 or more of these boxes? **IF YES:** Please tell me the box and number for each experience that occurred during the same 12-month period even if the problem didn't last the full 12 months. **CIRCLE SYMPTOMS THAT CLUSTER. MUST BE FROM 3 DIFFERENT**	NO . . . (SKIP TO BOX F21) 1<br>YES 5

BOXES. <u>DO NOT</u> COUNT SYMPTOMS THAT OCCURRED AS A RESULT OF AN ISOLATED INCIDENT.

B. How old were you the (first/last) time you had experiences from 3 or more boxes within a 12-month period?

AGE ONS: ___/___

AGE REC: ___/___

ICD-10

BOX F21 IF 3 OR MORE BOXES MARKED ON TALLY SHEET C, CONTINUE. OTHERS SKIP TO BOX F22.

(20)

HAND R MARIJUANA TALLY C.

F21 A. Please review these experiences which are grouped into boxes that are different from the other lists I showed you. You told me **(REVIEW SX)**. Was there ever a period lasting a month or longer when you had experiences from 3 or more different boxes occurring together? **IF YES:** Which ones? **CIRCLE SYMPTOMS THAT CLUSTER. MUST BE 3 FROM DIFFERENT BOXES. <u>DO NOT</u> COUNT SYMPTOMS THAT OCCURRED AS A RESULT OF AN ISOLATED INCIDENT.**

NO 1
YES. . . . .(SKIP TO C) 5

B. Have 3 or more experiences from different boxes occurred together repeatedly within a 12-month period even if the problem didn't last for the full 12 months? **IF YES:** Which ones? **CIRCLE SYMPTOMS THAT CLUSTER. <u>DO NOT</u> COUNT SYMPTOMS THAT OCCURRED AS A RESULT OF AN ISOLATED INCIDENT.**

NO. . . (SKIP TO BOX F22) 1
YES 5

C. How old were you the (first/last) time?

AGE ONS: ___/___

AGE REC: ___/___

BOX F22 IF 2+ BOXES MARKED ON TALLY A, CONTINUE. OTHERS SKIP TO F23.

(22) F22 Since the age of (ONS), has there ever been a period of time lasting 3 months or longer when you did not use marijuana at all?

NO(SKIP TO F23) 1
YES 5

A. When did that/these occur?
IF R HAD MORE THAN 4 ABSTINENT PERIODS, RECORD THE 4 LONGEST.

FROM __ __/__ __ TO____/__ _ __ *t*
MO YEAR MO YEAR

FROM __ __/__ __TO__ __/__ __ *t*
MO YEAR MO YEAR

t

FROM __ __/__ __ TO ____/ __ __
MO YEAR MO YEAR *t*

FROM __ __/__ __ TO ___/_ __ __

MO YEAR MO YEAR

(18) F23 Did you ever bring up any problems you might have had with marijuana with any professional?

NO(SKIP TO F24) 1
YES 5

A. To whom did you speak first?
1. A psychiatrist
2. Another medical doctor
3. A psychologist
4. Another mental health professional
5. A member of the clergy
6. Other: **SPECIFY:_________**

CODE: ___

B. How old were you the (first/last) time you brought up problems with marijuana with a professional?

AGE ONS: ___/___
ONS: 1 2 3 4 5

AGE REC: ___/___
REC:1 2 3 4 5

REFER TO B9 BEFORE ASKING

F24 Have you ever been treated for a problem with marijuana?

NO . . . .(SKIP TO D) 1
YES 5

A. Were you ever treated at:	NO	YES
1. NA or another self-help group?.	1	5
2. outpatient drug program?. . . .	1	5

3. outpatient, other?.	1	5
4. inpatient drug program?	1	5
5. inpatient for medical complications due to marijuana use?.	1	5
6. other? (**IF YES, SPECIFY**)	1	5

SPECIFY:

____

B. How old were you the (first/last) time you were treated?

AGE ONS: ___/___
ONS: 1 2 3 4 5

AGE REC: ___/___
REC:1 2 3 4 5

C. Where were you <u>first</u> treated? **RECORD CODE (1-6) AND THEN SKIP TO G1, p. 56.**

CODE: ___

D. Did you ever attend a self-help group (like NA) for your marijuana use?

NO. . . (SKIP TO G1, p. 56) 1
YES 5

1. How old were you the (first/last) time you attended a self-help group for your marijuana use?

AGE ONS:___/___
1 2 3 4 5

AGE REC:___/___
1 2 3 4 5

			1	2	3	4	5	6	7	8	9
	**HAND R CARD G.**		*COC*	*STIM*	*SED*	*OP*	*PCP*	*HAL*	*SOL*	*COMB*	*OTH*
G1	Have you ever used any of these drugs to feel good or high, or to feel more active or alert? Or did you use any prescription drugs when they were not prescribed, or more than prescribed?	NO	1	1	1	1	1	1	1	1	1
		YES	5	5	5	5	5	5	5	5	5

BOX G1 IF ALL NO, SKIP TO I1, p. 88. OTHERS CONTINUE FOR EACH DRUG CODED 5.

		1	2	3	4	5	6	7	8	9
A. How many times in your life have you used (DRUG)?	TIMES	__	___	___	___	___	___	___	___	___
1. **IF DK, ASK:** Would you say 11 or more times?	NO	1	1	1	1	1	1	1	1	1
	YES	5	5	5	5	5	5	5	5	5

B. How old were you the (first/last) time you used (DRUG)?

FOR EACH AGE ONS BEFORE 15, ASK C. OTHERS SKIP TO D.

C. Did you use (DRUG) more than once before you were 15?

	1	2	3	4	5	6	7	8	9
	COC	*STIM*	*SED*	*OP*	*PCP*	*HAL*	*SOL*	*COMB*	*OTH*
AGE ONS	___	___	___	___	___	___	___	__	__
ONS	___	___	___	___	____	___	___	___	
AGE REC	___	___	____	___	____	___	____	__	_
REC	___	___	____	____	____	___	___	___	_
NO	1	1	1	1	1	1	1	1	
YES	5	5	5	5	5	5	5	5	

IF NEVER USED COCAINE, SKIP TO F.

D. When you first started using cocaine, did you find that you got higher or stayed high longer than other people who would use the same amount of cocaine?

NO 1
YES 5

E. Did you ever use alcohol to make yourself feel better when coming down from the effects of cocaine?

NO 1
YES 5

F. Have you ever injected any of these drugs? **IF YES:** Which ones? **IF NO, SKIP TO H.**

NO 1
YES 5

1. How many times? TIMES __ __ __

2. How old were you the (first/last) time?

AGE ONS __ __

ONS __

AGE REC __ ___

REC __

G. Have you ever shared a needle? YES..5

1. How many times? __ __ __ TIMES

2. How old were you the (first/last) time?

AGE ONS: ___/___
ONS: 1 2 3 4 5

AGE REC: ___/___
REC: 1 2 3 4 5

H. Of all the drugs you have used, which one was your favorite (including marijuana)? **DO NOT COUNT ALCOHOL.**

_________________________ __ __ __
(CODE)

IF R USED ONLY 1 DRUG, SKIP TO BOX G2. OTHERS CONTINUE.

I. Have you ever used 2 or more drugs together (other than with marijuana and/or alcohol)?

NO..1
YES................ (SPECIFY) 5

1a. ___________________ b. ___________________ CODE: __ __ __ CODE: __ __ __

2a. ___________________ b. ___________________ CODE: __ __ __ CODE: __ __ __

BOX G2 CHECK G1A. IF NO DRUG USED 11 OR MORE TIMES, SKIP TO I1, p. 88.

IF USED COCAINE, STIMULANTS, SEDATIVES, AND/OR OPIATES 11 OR MORE TIMES, CONTINUE. IF USED OTHER DRUGS 11 OR MORE TIMES, CONTINUE WITH ONE USED MOST AND CODE IN COL. 5.

IF "OTHER" COLUMN USED, RECORD: ________________________ CODE: __ __ __

ASK ONE COLUMN AT A TIME. *COC STIM SED OP OTH*

				COC	STIM	SED	OP	OTH
FGNDRC	G2	What is the longest period you used (DRUG) almost every day? **IF NEVER ALMOST EVERY DAY, CODE 0 DAYS. IF USED DAILY FOR 1 MONTH OR LONGER, CODE G2A "YES" SILENTLY.**	DAYS	__		__	__	__
			WEEKS	__		__	__	__
			MONTHS	__		__	__	__
			YEARS	__		__	__	__
		A. Did you ever use (DRUG) at least once a week for one month or more?	NO	1		1	1	1
			YES	5		5	5	5
		B. Think about the time when you were using (DRUG) the most. During that period, how many days per month did you use (DRUG)?	DAYS PER MO	__		__	__	__	__
		1. How long did that period last?	MONTHS	__		__	__	__	__
		2. During that period of heaviest use, how many times did you use (DRUG) on an average day?	TIMES PER DAY	__		__	__	__	__
		3. How old were you when that period started?	AGE ONS	____	____	____	____	____

				1	2	3	4	5
(3) FGNDRC	G3	Have you ever stayed high from (DRUG) for a whole day or more?	NO	1		1	1	1
			YES	5		5	5	5
		A. **IF YES**: Did this happen 3 or more times?	NO	1	1	1	1	1
			YES	5	5	5	5	5
(4)	G4	OMITTED						

BEGIN SCORING DRUG TALLY SHEETS A, B, & C

(5)<br>DDICD1	G5	Have you ever had such a strong desire for (DRUG) that it was hard to think of anything else?	NO<br>YES	1<br>5	1<br>5	1<br>5	1<br>5	
		A. **IF YES:** How old were you the (first/last) time?	AGE ONS:<br>ONS:<br>AGE REC:<br>REC:	__<br>__<br>__<br>__	__<br>__<br>__<br>__	__<br>__<br>__<br>__	__<br>__<br>__<br>__	
(6)<br>DD3RA3/B<br>DD45<br>DDICD5<br>FGNDRC	G6	Was there ever a period of a month or more when a great deal of your time was spent using (DRUG), getting (DRUG), or getting over its effects?	NO<br>YES	1<br>5	1<br>5	1<br>5	1<br>5	
(7)<br>DD3RA2<br>DD44<br>DDICD2	G7	Have you often wanted to stop or cut down on (DRUG)?	NO<br>YES	1<br>5	1<br>5	1<br>5	1<br>5	
DD3RA2		A. Have you ever tried to stop or cut down on (DRUG) but found that you couldn't?	NO<br>YES	1<br>5	1<br>5	1<br>5	1<br>5	5 A
		**IF NO (COULD STOP), SKIP TO G8. OTHERS CONTINUE.**						
DD44<br>DDICD2		B. Were you unable to stop or cut down 3 or more times?	NO<br>YES	1<br>5	1<br>5	1<br>5	1<br>5	5 B,C
(8)<br>DD3RA7<br>DD41<br>DDICD4	G8<br>get<br>on	Did you ever need larger amounts of (DRUG) to an effect or find that you could no longer get high the amount you used to use?	NO<br>YES	1<br>5	1<br>5	1<br>5	1<br>5	

						1 COC	2 STIM	3 SED	4 OP	5 OTH
(9)	G9	Have you ever given up or greatly reduced important activities while using (DRUG), like sports, work, or associating with friends or relatives? **SPECIFY:**______________________________ ____	NO<br>YES	1<br>5		1<br>5	1<br>5		1<br>5	1<br>5
DD3RA5/B<br>DD46<br>DDICD5		A. **IF YES:** Did this happen 3 or more times or for a month or more?	NO<br>YES	1<br>5		1<br>5	1<br>5		1<br>5	1<br>5 A,B,C
(10)<br>DD3RA1<br>DD43<br>DDICD2	G10	Have you often used (DRUG) more days or in larger amounts than you intended to?	NO<br>YES	1<br>5		1<br>5	1<br>5	1<br>5	1<br>5 A,B,C	

(11) G11 People who stop, cut down, or go without drugs after using drugs steadily for some time may not feel well. These feelings are more intense and can last longer than the usual hangover. When you stopped, cut down, or went without (DRUG), did you ever experience any of the following problems <u>for most of the day for 2 days or longer</u>? **(NO=1, YES=5)**

ASK G11A-F ONE COLUMN AT A TIME.
REPEAT STEM OFTEN.

		1 COC	2 STIM	3 SED	4 OP	5 OTH
A. 1.	Did you feel depressed?	__	__	__	__	__
2.	Did you feel restless?	__	__	__		__
3.	Did you feel tired, sleepy, or weak?	__	__	__		__
4.	Did you have trouble sleeping?	__	__	__	__	__
5.	Did you sleep too much?	__	__			__
6.	Did you have a strong desire or craving for (DRUG)?	__	__		__	__
7.	Did you feel slowed down, like you could hardly move?	__	__			__
8.	Did you have an increase in appetite?	__	__			__
9.	Did you have nightmares?	__	__			__
10.	Did you have diarrhea?				__	__
11.	Did you have stomach aches or stomach cramps?				__	__
12.	Did your eyes run?				__	__
13.	Did your nose run?				__	__
14.	Did you have muscle pains?				__	__
15.	Did you yawn?				__	__
16.	Were your pupils dilated or were your eyes sensitive to light?				__	__
17.	Did you have gooseflesh, goose bumps, or did you get the chills?				__	__
18.	Did your heart race?			__	__	__
19.	Did you sweat?			__	__	__
20.	Did you have a fever?			__	__	__
21.	Did you have nausea, or did you vomit?			__	__	__
22.	Did you have headaches?			__		__
23.	Did you feel nervous, tense, or irritable?			__		__
24.	Did your hands shake?			__		__
25.	Did you tremble or twitch?			__		__
26.	Did you experience dizziness?			__		__
27.	Did you have seizures?			__		__
28.	Did you see, hear, or feel things that weren't really there?			__		__
29.	Did you think that people were plotting to harm you (PARANOID)?			__		__

CONTINUE ASKING ONE COLUMN AT A TIME.
FOR EACH DRUG COLUMN:
IF ALL CODED 1, GO TO NEXT DRUG COLUMN.
IF ONLY ONE CODED 5, SKIP TO E.
IF TWO OR MORE 5'S CODED, CONTINUE.

Code	Question		**1** *COC*	**2** *STIM*	**3** *SED*	**4** *OP*	**5** *OTH*	
DD3RA8 DD42A DDICD3	B. Was there ever a time when 2 or more of these problems occurred together because of stopping, cutting down on, or going without (DRUG)? **REVIEW SX AS NEEDED. IF NO, SKIP TO C.**	NO	1	1	1	1	1	
		YES	5	5	5	5	5	A,B,C
	1. **IF YES:** Did these problems occur <u>together</u> for 2 days or longer? **IF NO, SKIP TO C.**	NO	1	1	1	1	1	
		YES	5	5	5	5	5	
		AGE ONS	__	__	__	__	__	
		ONS	__	__	__	__	__	
	2. **IF YES:** How old were you the (first/last) time?	AGE REC	__	__	__	__	__	
		REC	__	__	__	__	__	
DD3RB	C. Did you have any of these problems 3 or more times?	NO	1	1	1	1	1	
		YES	5	5	5	5	5	
	D. Did these problems interfere with your functioning at work, school, or home?	NO	1	1	1	1	1	
		YES	5	5	5	5	5	
	E. Have you ever used (DRUG) to keep from having any of these problems (or to make them go away)? **IF NO, SKIP TO NEXT DRUG. IF NO DRUG, SKIP TO G12.**	NO	1	1	1	1	1	
		YES	5	5	5	5	5	
	1. **IF YES:** How old were you the (first/last) time?	AGE ONS	__	__	__	__	__	
		ONS	__	__	__	__	__	
		AGE REC	__	__	__	__	__	
DD3RA9/B DD42B DDICD3	2. Did you do that 3 or more times?	NO	1	1	1	1	1	
		YES	5	5	5	5	5	A,B,C

				1 COC	2 STIM	3 SED	4 OP	5 OTH	
(12B-D)	G12	Did using (DRUG) cause you to have any other problems like:							
		A. an overdose?	NO	1	1	1	1	1	
			YES	5	5	5	5	5	
DD3RA6/B<br>DD47<br>DDICD6<br>DA3RA1/B		1. **IF YES:** Did you require medical treatment afterwards?	NO	1	1	1	1	1	
			YES	5	5	5	5	5	
		2. **IF YES:** Did this happen 3 or more times? (overdose that required medical treatment)	NO	1	1	1	1	1	
			YES	5	5	5	5	5	A,B,C
		B. hepatitis?	NO	1	1	1	1	1	
			YES	5	5	5	5	5	
DD3RA6/B<br>DD47<br>DDICD6<br>DA3RA1		1. **IF YES:** Did you continue to use (DRUG) knowing it caused hepatitis?	NO	1	1	1	1	1	
			YES	5	5	5	5	5	A,B,C
		C. other serious health problems? **SPECIFY:** ______________________________	NO	1	1	1	1	1	
			YES	5	5	5	5	5	
DD3RA6<br>DD47<br>DDICD6<br>DA3RA1		1. **IF YES:** Did you continue to use (DRUG) knowing it caused health problems?	NO	1	1	1	1	1	
			YES	5	5	5	5	5	A,B,C
(13A-C)	G13	A. Were there ever objections from or problems with your family, friends, doctor, clergy, boss or people at work or school because of your (DRUG) use?	NO	1	1	1	1	1	
			YES	5	5	5	5	5	
		B. Did you ever get into any physical fights while using (DRUG)?	NO	1	1	1	1	1	
			YES	5	5	5	5	5	

BOX G13 IF A AND B ARE BOTH CODED 1, SKIP TO G14. OTHERS CONTINUE.

				1 COC	2 STIM	3 SED	4 OP	5 OTH	
DA4A4		C. Did (this/either of these experiences) happen 3 or more times in any 12-month period?	NO	1	1	1	1	1	
			YES	5	5	5	5	5	
DD3RA6<br>DA3RA1		D. Did you continue to use (DRUG) after you realized it was causing you any problem?	NO	1	1	1	1	1	
			YES	5	5	5	5	5	A
(13D)	G14	Did you ever have trouble with the police because of (DRUG)? **IF NO, SKIP TO G15.**	NO	1	1	1	1	1	
			YES	5	5	5	5	5	
DA4A3		A. **IF YES:** Did this happen 3 or more times in any 12-month period?	NO	1	1	1	1	1	
			YES	5	5	5	5	5	

DD3RA6<br>DA3RA1		B.	Did you continue to use (DRUG) after you realized it was causing you trouble with the police?	NO<br>YES	1<br>5	1<br>5	1<br>5	1<br>5	1<br>5 A

				1		2		
			COC	*STIM*		*SED*	*OP*	*OTH*
(12A)	G15	Have you accidentally injured yourself when you were using (DRUG); that is had a bad fall, cut or burned yourself badly, got hurt in a traffic accident, or anything like that? **IF NO, SKIP TO G16.**	NO<br>YES	1<br>5	1<br>5	1<br>5	1<br>5	1<br>5
DD3RA4/B<br>DDICD6<br>DA3RA2/B		A. **IF YES:** Did this happen 3 or more times?	NO<br>YES	1<br>5	1<br>5	1<br>5	1<br>5	1<br>5 A,C
DA4A2		B. **IF YES:** Did this happen 3 or more times in any 12-month period?	NO<br>YES	1<br>5	1<br>5	1<br>5	1<br>5	1<br>5

---

(14)<br>DD3RA4/B	G16	Has your being high on (DRUG) or experiencing its after-effects <u>often</u> interfered with your work, school, household, or child care responsibilities? **IF NO, SKIP TO G17.**<br><br>**IF YES, SPECIFY:**_____________________________<br>____	NO<br>YES	1<br>5	1<br>5	1<br>5	1<br>5	1<br>5 A
DA4A1		A. **IF YES:** Did this happen 3 or more times in any 12-month period?	NO<br>YES	1<br>5	1<br>5	1<br>5	1<br>5	1<br>5

---

(16)<br>DD3RA4/B<br>DA3RA2/B<br>ASP3RC7<br>ASP4A5	G17	Have there been 3 or more times when you have been under the influence of (DRUG) in a situation where it increased your chances of getting hurt--for instance, when driving a car or boat; using knives, machinery, or guns; crossing against traffic; climbing; or swimming? **IF NO, SKIP TO G18.**	NO<br>YES	1<br>5	1<br>5	1<br>5	1<br>5	1<br>5 A
DA4A2		A. **IF YES:** Did this happen 3 or more times in any 12-month period?	NO<br>YES	1<br>5	1<br>5	1<br>5	1<br>5	1<br>5

				COC	STIM	SED	OP	OTH
					1	2	3	
(15)	G18	Has your use of (DRUG) ever caused you emotional or psychological problems like:						
		1. Feeling depressed or uninterested in things for more than 24 hours to the point that it interfered with your functioning?	NO<br>YES	1<br>5	1<br>5	1<br>5	1<br>5	1<br>5
		2. Feeling paranoid or suspicious of people for more than 24 hours to the point that it interfered with your relationships?	NO<br>YES	1<br>5	1<br>5	1<br>5	1<br>5	1<br>5
		3. Having trouble concentrating or thinking clearly for more than 24 hours to the point that it interfered with your functioning?	NO<br>YES	1<br>5	1<br>5	1<br>5	1<br>5	1<br>5
		4. Hearing, seeing, or smelling things that weren't really there?	NO<br>YES	1<br>5	1<br>5	1<br>5	1<br>5	1<br>5
		5. Feeling jumpy or easily startled or nervous for more than 24 hours to the point that it interfered with your functioning?	NO<br>YES	1<br>5	1<br>5	1<br>5	1<br>5	1<br>5
		**IF ALL ARE CODED 1, SKIP TO G19. OTHERS CONTINUE.**						
DD3RA6<br>DD47<br>DDICD6<br>DA3RA1		A. Did you continue to use (DRUG) after you knew it caused any of these problems? **REVIEW SX AS NEEDED.**	NO<br>YES	1<br>5	1<br>5	1<br>5	1<br>5	1<br>5 A,B,C

BOX G19 IF ANY MARKS ON TALLY A, CONTINUE. OTHERS SKIP TO BOX G20.

DSMIIIR

		1	2	3	4	5
		COC	*STIM*	*SED*	*OP*	*OTH*

HAND R DRUG TALLY A.

(19) G19 Please review these experiences that you told me about. (**REVIEW SX.)** When was the (first/last) time you had any of these experiences?

AGE ONS:	__	__	__	__	__
ONS:	__	__	__	__	__
AGE REC:	__	__	__	__	__
REC:	__	__	__	__	__

BOX G19A IF 3 OR MORE BOXES MARKED ON TALLY A, CONTINUE. OTHERS SKIP TO BOX G20.

DD3RB

A. Was there ever a period lasting a month or longer when you had experiences from 3 or more boxes occurring together? **IF YES:** Please tell me the box and number for all the experiences that occurred together. **CIRCLE SYMPTOMS THAT CLUSTER. MUST BE 3 FROM DIFFERENT BOXES.** **<u>DO NOT</u> COUNT SYMPTOMS THAT OCCURRED AS A RESULT OF AN ISOLATED INCIDENT.**

NO	1	1	1	1	1
YES	5	5	5	5	5

IF YES, CIRCLE SX THAT CLUSTER AND SKIP TO C.

IF NO, ASK B.

B. Was there ever a period lasting a month or longer when you had experiences from 2 boxes occurring together?

IF YES: Please tell me the box and number for all the experiences that occurred together. **CIRCLE SYMPTOMS THAT CLUSTER. MUST BE FROM 2 DIFFERENT BOXES.** **<u>DO NOT</u> COUNT SYMPTOMS THAT OCCURRED AS A RESULT OF AN ISOLATED INCIDENT.**

NO	1	1	1	1	1
YES	5	5	5	5	5

IF YES, CIRCLE SX THAT CLUSTER AND ASK C.

IF NO, SKIP TO BOX G20.

C. How old were you the (first/last) time you had experiences from 3(2) boxes occur within a period lasting a month or longer?	AGE ONS: __ __ __ __ __ *t*<br>ONS: __ __ __ __ __ *t*<br>AGE REC: __ __ __ __ __ *t*<br>REC: __ __ __ __ __ *t*

DSM-IV	**BOX G20** IF 3 OR MORE BOXES MARKED ON TALLY B, CONTINUE. OTHERS SKIP TO BOX G21.

HAND R DRUG TALLY B.

				1	2	3	4	5
					COC	*STIM*	*SED*	*OP*
OTH DD4	G20	A. Was there ever a 12-month period in which you had experiences from 3 or more boxes? **IF YES:** Please tell me the box and number for all the experiences that occurred during the same 12-month period even if it didn't last the full 12 months. **CIRCLE SX THAT CLUSTER. MUST BE FROM 3 DIFFERENT BOXES.** DO NOT COUNT SX RESULTING FROM AN ISOLATED INCIDENT.	NO	1	1	1	1	1
			YES	5	5	5	5	5

IF YES, CIRCLE SX THAT CLUSTER AND ASK B.
IF NO, SKIP TO BOX G21.

B. How old were you the (first/last) time you had experiences from 3 or more boxes occur together within a period lasting 12 months or longer?	AGE ONS:	__	__	__	__
	ONS:	__	__	__	__
	AGE REC:	__	__	__	__
	REC:	__	__	__	__

BOX G21 IF 3 OR MORE BOXES MARKED ON TALLY C, CONTINUE. OTHERS SKIP TO BOX G22.

HAND R DRUG TALLY C.

				1	2	3	4	5
ICD-10 (20) DDICD	G21	A. Was there ever a period lasting a month or longer when you had experiences from 3 or more boxes occurring together? **IF YES:** Please tell me the box and number for all the experiences that occurred together. **CIRCLE SX THAT CLUSTER. MUST BE FROM 3 DIFFERENT BOXES.** DO NOT COUNT SX RESULTING FROM AN ISOLATED INCIDENT.	NO	1	1	1	1	1
			YES	5	5	5	5	5

IF YES, CIRCLE SX THAT
IF NO, ASK B.

	B. Have experiences from 3 or more boxes occurred together repeatedly within any 12-month period?	NO	1 1 1 1 1
		YES	5 5 5 5 5
	**IF YES:** Please tell me the box and number for each experience. **CIRCLE SX THAT CLUSTER. MUST BE FROM 2 DIFFERENT BOXES.**	**IF YES, CIRCLE SX THAT** **IF NO, SKIP TO BOX G22.**	
DDICD	C. How old were you the (first/last) time you had experiences from 3 or more boxes occur together within a period lasting (1 month/12 months) or longer?	AGE ONS:	__ __ __ __
		ONS:	__ __ __ __ __
		AGE REC:	__ __ __ __ __
		REC:	__ __ __ __ __

BOX G22 IF 2 OR MORE BOXES MARKED ON TALLY A, CONTINUE. OTHERS SKIP TO G23.

(22) G22 Since the age of (ONS), has there ever been a period of time lasting 3 months or longer when you did not use (DRUG) at all? **FOR EACH YES, ASK A.**

	**1**	**2**	**3**	**4**	**5**
	COC	*STIM*	*SED*	*OP*	*OTH*
NO	1	1	1	1	1
YES	5	5	5	5	5

A. When did (that/these) occur?

	MO YEAR		MO YEAR		MO YEAR		MO YEAR	
COC	__ __/__ __ __ __	TO	__ __/__ __ __ __;		__ __/__ __ __ __	TO	__ __/__ __ __ __	*t*
	__ __/__ __ __ __	TO	__ __/__ __ __ __;		__ __/__ __ __ __	TO	__ __/__ __ __ __	*t*
STIM	__ __/__ __ __ __	TO	__ __/__ __ __ __;		__ __/__ __ __ __	TO	__ __/__ __ __ __	*t*
	__ __/__ __ __ __	TO	__ __/__ __ __ __;		__ __/__ __ __ __	TO	__ __/__ __ __ __	*t*
SED	__ __/__ __ __ __	TO	__ __/__ __ __ __;		__ __/__ __ __ __	TO	__ __/__ __ __ __	*t*
	__ __/__ __ __ __	TO	__ __/__ __ __ __;		__ __/__ __ __ __	TO	__ __/__ __ __ __	*t*
OP	__ __/__ __ __ __	TO	__ __/__ __ __ __;		__ __/__ __ __ __	TO	__ __/__ __ __ __	*t*
	__ __/__ __ __ __	TO	__ __/__ __ __ __;		__ __/__ __ __ __	TO	__ __/__ __ __ __	*t*
OTH	__ __/__ __ __ __	TO	__ __/__ __ __ __;		__ __/__ __ __ __	TO	__ __/__ __ __ __	*t*
	__ __/__ __ __ __	TO	__ __/__ __ __ __;		__ __/__ __ __ __	TO	__ __/__ __ __ __	*t*

---

(17) G23 Have you ever brought up any problem you might have had with drugs with any professional?

NO........ (SKIP TO G24) 1
YES 5

A. Did you speak with:

	NO	YES
1. A psychiatrist?	1	5
2. Another medical doctor?	1	5
3. A psychologist?	1	5
4. Another mental health professional?	1	5
5. A member of the clergy?	1	5
6. Anyone else? **SPECIFY:** __________________________	1	5

IF ALL ARE CODED 1, SKIP TO G24.
OTHERS CONTINUE.

B. How old were you the (first/last) time you brought up any problem you had with drugs?

AGE ONS: ___/___
ONS: 1 2 3 4 5

AGE REC: ___/___
REC: 1 2 3 4 5

C. With whom did you speak first?
RECORD CODE (1-6).

CODE: ___

---

(18) G24 Have you ever been treated for a problem with drugs?

NO.......... (SKIP TO D) 1
YES 5

A. Were you treated:

		NO	YES
	1. at NA or another self-help group?	1	5
	2. at an outpatient drug-free program?	1	5
	3. at an outpatient program for something other than drugs?	1	5
FGNDRB	4. at an inpatient drug-free program?	1	5
FGNDRB	5. when inpatient for medical complications due to drug use?	1	5
	6. at any other place or program? **IF YES, SPECIFY.**	1	5

SPECIFY:____

B. How old were you the (first/last) time you were treated for a drug problem?

AGE ONS: ___/___
ONS: 1 2 3 4 5

AGE REC: ___/___
REC: 1 2 3 4 5

C. Where were you treated first?
RECORD CODE (1-6) AND THEN SKIP TO H1, p. 68.

CODE: ___

D. Did you ever attend a self-help group (like NA) (like NA) because you had a problem with drugs?

NO. . . (SKIP TO I1, p. 88) 1
YES....................................... 5

1. How old were you the (first/last) time you attended a self-help group for drug

AGE ONS: ___/___
ONS: 1 2 3 4 5

AGE REC: ___/___
REC: 1 2 3 4 5

Now I'm going to ask you some questions about your mood.

DEPRDCA

I1	Have you ever had a period of time lasting at least one week when you were bothered most of the day, nearly every day, by feeling depressed, sad, blue, or irritable?	NO .... 1<br>YES 5
I2	Have you ever had a period of time lasting at least one week when you lost interest or enjoyment in almost everything, even things you usually liked to do?	NO .... 1<br>YES 5

BOX I2 IF I1 AND I2 BOTH CODED 1, SKIP TO J1, p. 104.
OTHERS CONTINUE.

I3	During the past 30 days, have you been feeling depressed, uninterested in things, or unable to enjoy almost everything most of the day, nearly everyday, for at least one week?	NO .... (SKIP TO I4).............. 1<br>YES 5
	A. For how long have you felt this way?	___ ___ ___ WEEKS

BOX I3 SKIP TO I5.

I4 Please tell me about the time in your life that stands out as the most severe period of feeling depressed, uninterested in things or irritable most of the day, nearly everyday. When did it begin?

___ ___ / ___
MO YEAR

DESCRIPTION:_______________________________________

____

____

A. So you were ____ years old? AGE: ___ ___

B. How long did that episode last? ___ ___ ___ WEEKS

BOX I4

**A.**	**DOES A CURRENT EPISODE EXIST (I3=5)?**	**NO . . (SKIP TO I5) ...1**<br>**YES 5**
**B.**	**IS THE EPISODE IN I4 THE CURRENT EPISODE?**	**NO . . (SKIP TO I5) ...1**<br>**YES 5**
**C.**	**IS THIS EPISODE CLEAN (BOX I13A=5)?**	**NO . . (GO TO I5)..........1**<br>**YES . (SKIP TO I34)..5**

Sometimes people have episodes of depression that follow the death of a loved one, heavy drinking or drug use, a change in medication, or a serious illness (or childbirth).

		CURRENT EPISODE (PAST MONTH)	MOST SEVERE EPISODE
(31)	I5 During the 6 weeks before this episode of feeling (depressed/ uninterested/irritable) began, how many days a week did you typically drink alcohol?	DAYS: ___<br>**IF 0 OR 1, SKIP TO I6. OTHERS CONTINUE.**	DAYS: ____<br>**IF 0 OR 1, SKIP TO I6. OTHERS CONTINUE.**
	A. On the days you drank, how many drinks would you typically have in a day?	DRINKS: __ __	DRINKS: __ __
DEP3RB1<br>DEP4D	**CODE SILENTLY:**<br>B. TYPICALLY 3+ (WOMAN) OR 5+ (MAN) DRINKS FOR 4+ DAYS/WEEK?	NO .... 1<br>YES. . . . (SKIP TO I6) 5*	NO .... 1<br>YES . . . (SKIP TO E) 5
	C. During the 6 weeks before this episode began, what was the largest number of drinks you had in one day?	DRINKS: __ __<br>**IF 4 OR FEWER, SKIP TO I6.**	DRINKS: __ __<br>**IF 4 OR FEWER, SKIP TO I6.**
DEP3RB1<br>DEP4D	D. Did you drink at least 5 drinks 2 or more times a week during the 6 weeks before this episode began?	NO .... 1<br>YES .... 5*<br>**SKIP TO I6.**	NO. . . (SKIP TO I6) .... 1<br>YES.................................... 5
	E. **MOST SEVERE ONLY:** Did you have another episode of feeling (depressed/ uninterested/irritable) for at least one week that did not follow a time when you had been drinking daily or almost daily (or heavily)?		NO. . . . . (SKIP TO I11)..... 1*<br>YES.................................... 5
	F. When did this episode begin?		___ ___ / ___ ___ ___ ___<br>MO YEAR
	1. How old were you?		AGE: ___ ___

**REMIND R WHICH EPISODE AS NEEDED.**	**CURRENT EPISODE (PAST MONTH)**	**MOST SEVERE EPISODE**
**IF NEVER USED MJ OR DRUGS (F1=1 AND G1=1), SKIP TO I7.**		

(30) DEP3RB1 DEP4D	**HAND R CARD I.**<br>I6 During the 6 weeks before <u>this</u> episode of feeling (depressed/ uninterested/irritable) began, did you use any of these street drugs or abuse any prescription drugs? **IF YES:** Which ones? **CIRCLE ON CARD I. CODE THE THREE USED MOST.**	NO. . . . (SKIP TO I7) .... 1<br>YES. . . . (SPECIFY) .... 5<br>1:____________ __ __ __<br>2:____________ __ __ __<br>3:____________ __ __ __	NO. . . . (SKIP TO I7) .... 1<br>YES. . . . (SPECIFY)........... 5<br>1:____________ __ __ __<br>2:____________ __ __ __<br>3:____________ __ __ __
	A. Did you take any of these drugs for a high or intoxication <u>daily or almost daily</u>? **IF YES:** Which ones?	NO. . . . (SKIP TO D) .... 1<br>YES. . . . (SPECIFY) .... 5*<br>CIRCLE DRUG: 1 2 3	NO. . . . (SKIP TO D).......... 1<br>YES . . . .(SPECIFY)........... 5<br>CIRCLE DRUG: 1 2 3
	B. During that time, on average, how many days per week did you take (DRUG) daily or almost daily?	DRUG 1: ___ DAYS<br>DRUG 2: ___ DAYS<br>DRUG 3: ___ DAYS	DRUG 1: ___ DAYS<br>DRUG 2: ___ DAYS<br>DRUG 3: ___ DAYS
	C. What is the <u>average</u> number of times you used (DRUG) on those days you used?	DRUG 1: ___ ___ AVG<br>DRUG 2: ___ ___ AVG<br>DRUG 3: ___ ___ AV	DRUG 1: ___ ___ AVG<br>DRUG 2: ___ ___ AVG<br>DRUG 3: ___ ___ AV
	D. During the 6 weeks before this episode began, what was the <u>largest</u> number of times you used (DRUG) in one day?	DRUG 1: ___ ___ MAX<br>DRUG 2: ___ ___ MAX<br>DRUG 3: ___ ___ MAX	DRUG 1: ___ ___ MAX<br>DRUG 2: ___ ___ MAX<br>DRUG 3: ___ ___ MAX
	E. On how many days during that 6-week period did you use (DRUG) that much (# IN D) in a day?	DRUG 1: ___ ___ DAYS<br>DRUG 2: ___ ___ DAYS<br>DRUG 3: ___ ___ DAYS<br>**SKIP TO I7.**	DRUG 1: ___ ___ DAYS<br>DRUG 2: ___ ___ DAYS<br>DRUG 3: ___ ___ DAYS<br>**IF I6A=1, SKIP TO I7. OTHERS CONTINUE.**
	F. **MOST SEVERE ONLY**: Did you have <u>another</u> episode of feeling (depressed/ uninterested/irritable) for at least one week when it was <u>not</u> after a time when you had been drinking or using drugs daily or almost daily?		NO. . . .(SKIP TO I11) ....1*<br>YES.................................... 5
	G. When did this episode begin?		___ ___ / ___ ___ ___ ___<br>MO YEAR
	1. How old were you?		AGE: ___ ___
	**REMIND R WHICH EPISODE AS NEEDED.**	**CURRENT EPISODE (PAST MONTH)**	**MOST SEVERE EPISODE**

(29) DEP3RB1 DEP4D	I7 Did this episode of feeling (depressed/uninterested/irritable) begin within 6 weeks of starting or changing the dose of prescription medication, such as tranquilizers, pills for high blood pressure, heart medicines, or steroids?	NO. . . . (SKIP TO I8) .... 1<br>YES. . . . .(SPECIFY) .... 5*<br>1. ____________ __ __ __<br>2. ____________ __ __ __<br>**SKIP TO I8.**	NO. . . . (SKIP TO I8) .... 1<br>YES. . . . .(SPECIFY) .... 5<br>1. ____________ __ __ __<br>2. ____________ __ __ __
	A. **MOST SEVERE ONLY**: Did you have another episode of feeling (depressed/ uninterested/irritable) for at least one week that was not after a time when you had a change in prescription medicines and was not after a time when you had been drinking or using drugs daily or almost daily?		NO. . . .(SKIP TO I11) ....1*<br>YES.................................... 5
	B. When did this episode begin?		___ ___ / ___ ___ ___ ___<br>MO YEAR
	1. How old were you?		AGE: ___ ___

**REMIND R WHICH EPISODE AS NEEDED**.		**CURRENT EPISODE (PAST MONTH)**	**MOST SEVERE EPISODE**
(28) DEP3RB2 DEP4E	I8 A. Did this episode of feeling (depressed/uninterested/ irritable) begin within 6 months of learning about the death of someone close to you?	NO. . . . (SKIP TO I9) .... 1<br>YES. . . (SPECIFY) .... 5*<br>RELATIONSHIP:<br>________________________<br>DATE OF DEATH:<br>___ ___ / ___ ___ ___ ___<br>MO YEAR<br>**SKIP TO I9.**	NO. . . .(SKIP TO I9) .... 1<br>YES. . . (SPECIFY)............. 5<br>RELATIONSHIP:<br>________________________<br>DATE OF DEATH:<br>___ ___ / ___ ___ ___ ___<br>MO YEAR
	B. **MOST SEVERE ONLY**: Did you have another episode of feeling (depressed/ uninterested/irritable) for at least one week that did not follow the death of someone close to you, was not after a time when you had a change in prescription medicines, and was not after a time when you had been drinking		NO. . . .(SKIP TO I11) ....1*<br>YES.................................... 5

	or using drugs daily or almost daily?		
	C. When did this episode begin?		___ ___ / ___ ___ ___ ___<br>MO YEAR
	1. How old were you?		AGE: ___ ___

**REMIND R WHICH EPISODE AS NEEDED**.	CURRENT EPISODE (PAST MONTH)	MOST SEVERE EPISODE
(26) DEP3RB1 DEP4D<br>I9 Did <u>this</u> episode of feeling (depressed/uninterested/ irritable) begin within the 6 weeks that followed an episode of a serious physical illness, like thyroid disease, a stroke, multiple sclerosis, a brain tumor, or AIDS?	NO. . (SKIP TO BOX I10).... 1<br>YES. . . . .(SPECIFY) .... 5*<br>________________________<br>—<br>**SKIP TO BOX I10.**	NO. . (SKIP TO BOX I10) ... 1<br>YES. . . .(SPECIFY)............ 5<br>________________________<br>_<br>CODE: __ __ __
A. **MOST SEVERE ONLY**: Did you have <u>another</u> episode of feeling (depressed/ uninterested/irritable) for at least one week that was <u>not</u> after a time when you had a serious physical illness, was <u>not</u> after the death of someone close to you, was <u>not</u> after a change in medication, and <u>not</u> after a time when you had been drinking or using drugs daily or almost daily?		NO. . . .(SKIP TO I11) ....1*<br>YES.................................... 5
B. When did this episode begin?		___ ___ / ___ ___ ___ ___<br>MO YEAR
1. How old were you?		AGE: ___ ___

BOX I10 **IF R IS MALE OR HAS NEVER BEEN PREGNANT, SKIP TO I12. OTHERS CONTINUE.**

**REMIND R WHICH EPISODE AS NEEDED**.	CURRENT EPISODE (PAST MONTH)	MOST SEVERE EPISODE
(27)<br>I10 Did this episode of feeling (depressed/uninterested/irritable) begin around the time of a	NO. . . (SKIP TO I12) .... 1<br>YES 5	NO. . . .(SKIP TO I12) .... 1<br>YES.................................... 5

childbirth, miscarriage, or abortion?		
A. Did it begin between the 2 weeks before to 6 weeks after the (birth/miscarriage/ abortion)?	NO .... 1<br>YES .... 5*<br><br>**SKIP TO I12.**	NO. . . . (SKIP TO I12) .... 1<br>YES.................................... 5
B. **MOST SEVERE ONLY**: Did you have another episode of feeling (depressed/ uninterested/irritable) for at least one week that was not around the time of childbirth, miscarriage, or abortion; was not after a time when you had a serious physical illness; was not after the death of someone close to you; was not after a change in medication; and was not after a time when you had been drinking or using drugs daily or almost daily?		NO. . . .(SKIP TO I11) ....1*<br>YES.................................... 5
C. When did this episode begin?		___ ___ / ___ ___ ___ ___<br>MO YEAR
1. How old were you?		AGE: ___ ___<br><br>**SKIP TO I12.**

I11 **IF NO CLEAN EPISODE EXISTS, CONTINUE THE SECTION ASKING ABOUT THE MOST SEVERE EPISODE CODED IN I4. REMIND R WHICH EPISODE TO FOCUS ON AS FREQUENTLY AS NEEDED.**

I'd like to (return to/focus on) the most severe episode of feeling (depressed/uninterested/irritable) when you were ____ years old **(CHECK I4A)**.

During this current episode . . .<br>During this most severe episode when you were _____ years old . . .	**CURRENT EPISODE (PAST MONTH)**	**MOST SEVERE EPISODE**
BEGIN SCORING +		
(3B/4C)<br>DEP3RA1<br>DEP4A1<br>DEPICDB1<br>FGNA<br><br>(3C/4D)<br><br>I12 Were you feeling depressed, sad, or blue most of the day, nearly every day, for at least 2 weeks during this episode?	NO .... 1<br>YES ....5+	NO .... 1<br>YES.................................. 5+

DEP3RA2<br>DEP4A2<br>DEPICDB2	A. Had you lost interest or enjoyment in most things most of the day, nearly every day, for at least 2 weeks during this episode?	NO .... 1<br>YES .... 5+<br><br>**IF EPISODE BEGAN BEFORE AGE 18, CONTINUE. OTHERS SKIP TO BOX I13.**	NO .... 1<br>YES .... 5+<br><br>**IF EPISODE BEGAN BEFORE AGE 18, CONTINUE. OTHERS SKIP TO BOX I13.**
DEP3RA1<br>DEP4A1	B. Did you feel irritable most of the day, nearly every day, for at least 2 weeks during this episode?	NO .... 1<br>YES .... 5+	NO .... 1<br>YES .... 5+

**BOX I13**	**A. IS EPISODE CLEAN? (DIRTY=ANY * IN I5-I10)**	**NO, DIRTY .... 1<br>YES, CLEAN 5**	**NO, DIRTY .... 1<br>YES, CLEAN 5**
	**B. DOES R ENDORSE LOW MOOD, LOSS OF INTEREST, OR IRRITABILITY? (I12, I12A, OR I12B CODED 5)**	**NO, DENIES .... 1<br>YES, ENDORSES 5**	**NO, DENIES .... 1<br>YES, ENDORSES 5**
	**C. IS MOST SEVERE EPISODE ALSO CURRENT?**		**NO .... 1<br>YES. . . (SKIP TO I34) .... 5<br>N/A 9**

Now I would like to ask you about other experiences you may have had during this episode of feeling (depressed/uninterested/irritable).

During this current episode . . .<br>During this most severe episode when you were _____ years old . . .			**CURRENT EPISODE (PAST MONTH)**	**MOST SEVERE EPISODE**
(5)<br>DEP3RA3<br>DEP4A3<br>DEPICDC7<br>RDCB1	I14	A. Did you have a change in appetite (that was not due to pregnancy, a physical condition, or dieting)?	NO.... (SKIP TO B) .... 1<br>YES .... 5+	NO....(SKIP TO B) .... 1<br>YES .... 5+
FGNB1		1. Increase or decrease?	INCREASE .... 2<br>DECREASE .... 3<br>BOTH 4	INCREASE .... 2<br>DECREASE .... 3<br>BOTH 4
DEP3RA3<br>DEP4A3<br>DEPICDC7<br>RDCB1		B. Did you gain or lose weight when you were not trying to (that was not due to pregnancy, a physical condition, or dieting)?	NO.... (SKIP TO I15) .... 1<br>YES .... 5+	NO.... (SKIP TO I15) .... 1<br>YES .... 5+
FGNB1		1. Gained or lost weight?	GAINED .... 2<br>LOST .... 3<br>BOTH 4	GAINED .... 2<br>LOST .... 3<br>BOTH 4
DEP3RA3<br>DEP4A3		C. What was your weight before	___ ___ ___ LBS	___ ___ ___ LBS

RDCB1	the (gain/loss)? **IF BOTH, CODE THE MORE SIGNIFICANT CHANGE.**		
DEP3RA3<br>DEP4A3<br>RDCB1	D. What was your weight after the (gain/loss)?	___ ___ ___ LBS	___ ___ ___ LBS
DEP3RA3<br>DEP4A3<br>RDCB1	E. Over what period of time did you (gain/lose) this amount of weight?	___ ___ ___ WEEKS	___ ___ ___ WEEKS

During this current episode . . .<br>During this most severe episode when you were _____ years old . . .		**CURRENT EPISODE (PAST MONTH)**	**MOST SEVERE EPISODE**
(6)<br>DEPICDD6<br>RDCB2<br>FGNB2	I15 Did you have more trouble sleeping than usual?	NO . . . . .(SKIP TO F) .... 1<br>YES 5	NO . . . . (SKIP TO F) .... 1<br>YES 5
	A. Were you unable to fall asleep?	NO . . . . (SKIP TO C) .... 1<br>YES 5	NO . . . . (SKIP TO C) .... 1<br>YES 5
DEP3RA4<br>DEP4A4	B. Was this for at least one hour?	NO....................................... 1<br>YES .... 5+	NO....................................... 1<br>YES .... 5+
DEP3RA4<br>DEP4A4	C. Did you wake up in the middle of the night and have trouble going back to sleep?	NO....................................... 1<br>YES .... 5+	NO....................................... 1<br>YES .... 5+
	D. Did you wake up too early in the morning?	NO . . . . (SKIP TO F)........... 1<br>YES 5	NO . . . .(SKIP TO F)........... 1<br>YES 5
DEP3RA4<br>DEP4A4	E. Was this at least one hour earlier than usual?	NO....................................... 1<br>YES .... 5+	NO....................................... 1<br>YES .... 5+
DEP3RA4<br>DEP4A4<br>DEPICDD6<br>RDCB2<br>FGNB2	F. Did you sleep much more than usual?	NO....................................... 1<br>YES .... 5+	NO....................................... 1<br>YES .... 5+
(7)<br>DEP3RA5<br>DEP4A5<br>DEPICDC5<br>RDCB4<br>FGNB4	I16 Were you so fidgety or restless that other people could have noticed?	NO....................................... 1<br>YES .... 5+	NO....................................... 1<br>YES .... 5+
(8)<br>DEP3RA5<br>DEP4A5<br>DEPICDC5<br>RDCB4<br>FGNB4	I17 Were you moving or speaking so slowly that other people could have noticed?	NO....................................... 1<br>YES .... 5+	NO....................................... 1<br>YES .... 5+
(9)<br>DEP3RA2<br>DEP4A2<br>FGNB5<br>RDCB5	I18 Were you much less interested in things or less able to enjoy sex or other pleasurable activities?	NO....................................... 1<br>YES .... 5+	NO....................................... 1<br>YES .... 5+
(10)<br>DEP3RA6<br>DEP4A6<br>DEPICDB3<br>RDCB3<br>FGNB3	I19 Were you feeling a loss of energy or were you more tired than usual?	NO....................................... 1<br>YES .... 5+	NO....................................... 1<br>YES .... 5+

		CURRENT EPISODE (PAST MONTH)	MOST SEVERE EPISODE
(11) DEP3RA7 DEP4A7 DEPICDC2 RDCB6 FGNB6	I20 Were you feeling excessively guilty or that you were a bad person?	NO....... 1<br>YES....... 5+	NO....... 1<br>YES....... 5+
During this current episode...<br>During this most severe episode when you were ____ years old...		**CURRENT EPISODE (PAST MONTH)**	**MOST SEVERE EPISODE**
(12) DEP3RA7 DEP4A7 DEPICDC1 RDCB6 FGNB6	I21 Were you feeling that you were a failure or worthless?	NO....... 1<br>YES....... 5+	NO....... 1<br>YES....... 5+
(13) DEP3RA8 DEP4A8 DEPICDC4 RDCB7 FGNB7	I22 Were you having more difficulty than usual thinking, concentrating, or making decisions?	NO....... 1<br>YES....... 5+	NO....... 1<br>YES....... 5+
(14) DEP3RA9 DEP4A9 DEPICDC3 RDCB8 FGNB8	I23 Did you have thoughts of dying, or taking your life, or wishing you were dead? **DO NOT COUNT THINKING ABOUT THE DEATH OF A RECENTLY DECEASED OR DYING LOVED ONE.**	NO....... 1<br>YES....... 5+	NO....... 1<br>YES....... 5+
DEP3RA9 DEP4A9 DEPICDC3	A. Did you make a plan for committing suicide?	NO....... 1<br>YES....... 5+	NO....... 1<br>YES....... 5+
DEP3RA9 DEP4A9 DEPICDC3	B. Did you try to kill yourself?	NO....... 1<br>YES....... 5+	NO....... 1<br>YES....... 5+
	**BOX I24 COUNT THE BOXES MARKED ON TALLY SHEET I**	**# OF BOXES: _____**<br><br>**IF FEWER THAN 4 BOXES: GO BACK TO I4 AND ASK ABOUT THE MOST SEVERE EPISODE.**<br><br>**IF ONLY 4 BOXES: SKIP TO I26.**<br><br>**IF 5 OR MORE BOXES: CONTINUE TO BOX I25.**	**# OF BOXES: _____**<br><br>**IF FEWER THAN 4 BOXES: CHECK # OF BOXES IN CURRENT EPISODE. IF ALSO FEWER THAN 4 IN CURRENT, SKIP TO I34. IF 4 OR MORE IN CURRENT, RECONCILE WITH SUBJECT AND RECODE BOX I13 AS NECESSARY.**<br><br>**IF ONLY 4 BOXES: SKIP TO I26.**<br><br>**IF 5 OR MORE BOXES: CONTINUE TO BOX I25.**

BOX I25 IF R DENIES LOW MOOD, LOSS OF INTEREST, AND IRRITABILITY (BOX I13B=1), SKIP TO I25B. OTHERS CONTINUE.

HAND R TALLY I.

		CURRENT EPISODE (PAST MONTH)	MOST SEVERE EPISODE
(16) ENDORSES MOOD	I25 A. You told me you experienced the following **(REVIEW HEADINGS OF BOXES ENDORSED)**. Did you feel (depressed/uninterested/ irritable) and have experiences from 4 or more other groups of problems nearly every day, for at least 2 weeks?	NO....... (SKIP TO I26) .... 1<br>YES .... 5<br><br>**IF YES:** Which ones?<br><br>**CIRCLE MOOD AND SX THAT CLUSTER. NOTE: BOX A OR BOX B MUST BE INCLUDED.**<br><br>**SKIP TO D.**	NO...... (SKIP TO I26).......... 1<br>YES .... 5<br><br>**IF YES:** Which ones?<br><br>**CIRCLE MOOD AND SX THAT CLUSTER. NOTE: BOX A OR BOX B MUST BE INCLUDED.**<br><br>**SKIP TO D.**
DENIES MOOD	B. You told me that during this episode you experienced **(REVIEW HEADINGS OF BOXES ENDORSED)**. During this episode, did you have experiences from 4 or more of these groups of problems nearly every day, for at least two weeks?	NO....... (SKIP TO I26) .... 1<br>YES .... 5<br><br>**IF YES:** Which ones?<br><br>**CIRCLE SX THAT CLUSTER.**	NO...... (SKIP TO I26).......... 1<br>YES .... 5<br><br>**IF YES:** Which ones?<br><br>**CIRCLE SX THAT CLUSTER.**
	C. During this period, did you also feel depressed or uninterested, (or irritable) in most things most of the day, nearly every day for at least 2 weeks?	NO....... (SKIP TO I26) .... 1<br>YES 5	NO...... (SKIP TO I26).......... 1<br>YES 5
	D. When did this episode begin (when you had these experiences nearly every day)?	___ ___ / ___ ___ ___ ___ *t*<br>MO YEAR	___ ___ / ___ ___ ___ ___ *t*<br>MO YEAR
DEP3RA DEP4A DEPICDA RDCC FGNC	E. For how long did you feel (depressed/uninterested/ irritable) and have experiences from at least 4 other groups of problems nearly every day?	___ ___ ___ WEEKS	___ ___ ___ WEEKS

(17) DEP4E RDCD	I26	During this episode, did you see or hear things that other people could not see or hear, that is, did you have hallucinations?	CODE: 1 2 3 4 5<br>SPECIFY: ________________<br>________________________<br>WHOM SAW: ____________<br>WHAT TOLD: ____________	CODE: 1 2 3 4 5<br>SPECIFY:________________<br>________________________<br>WHOM SAW:____________<br>WHAT TOLD:____________
DEP4E RDCD		A. During this episode, did you have beliefs or ideas that you later found out were <u>not</u> true?	CODE: 1 2 3 4 5<br>SPECIFY: ________________<br>________________________<br>WHOM SAW: ____________<br>WHAT TOLD: ____________	CODE: 1 2 3 4 5<br>SPECIFY:________________<br>________________________<br>WHOM SAW:____________<br>WHAT TOLD: ____________
			**IF ANY 5 IN I26 OR I26A CONTINUE.<br>OTHERS SKIP TO I27.**	
		B. Did these (beliefs/ideas/ hallucinations) occur before your (depressed mood/loss of interest/irritability)?	NO........ (SKIP TO D) .... 1<br>YES..................................... 5	NO....... (SKIP TO D) ....1<br>YES.....................................5
		C. How long before your (depressed mood/loss of interest/irritability) began did you have these (beliefs/ideas/ hallucinations)?	___ ___ ___ DAYS	___ ___ ___ DAYS
		D. Did you keep having these (beliefs/ideas/hallucinations) after your mood came back to normal?	NO...(SKIP TO BOX I26)...... 1<br>YES..................................... 5<br>EPISODE ONGOING .... 6<br>**IF ONGOING, SKIP TO BOX I26.**	NO.. (SKIP TO BOX I26)......1<br>YES.....................................5<br>EPISODE ONGOING.........6<br>**IF ONGOING, SKIP TO BOX I26.**
DEP3RC RDCD4		E. How long did they last after your mood came back to normal?	___ ___ ___ DAYS	___ ___ ___ DAYS
**BOX I26**		**DID EXAMPLES IN I26 AND I26A HAVE CONTENT THAT WAS ENTIRELY CONSISTENT WITH THEMES OF PERSONAL INADEQUACY, GUILT, POVERTY, PUNISHMENT, ILLNESS, OR CATASTROPHE?**	**NO .... 1<br>YES.................................... 5**	**NO......................................1<br>YES....................................5**
			**CURRENT EPISODE (PAST MONTH)**	**MOST SEVERE EPISODE**
(19)	I27	During this episode, were you seen by a doctor, or other professional?	NO....... (SKIP TO I31) .... 1<br>YES........(SPECIFY) 5	NO...... (SKIP TO I31)..........1<br>YES.......(SPECIFY) 5

			______<br>______	______<br>______
(20)	I28	During this episode, were you prescribed medicine for depression (or were you already taking medicine for depression)?	NO .... 1<br>YES ....(SPECIFY) .... 5<br>1.__________ __ __ __<br>2.__________ __ __ __	NO .... 1<br>YES ....(SPECIFY) .... 5<br>1.__________ __ __ __<br>2.__________ __ __ __
(21)	I29	During this episode, did you receive ECT (shock treatments)?	NO .... 1<br>YES 5	NO .... 1<br>YES 5
(22)<br>RDCC	I30	During this episode, were you hospitalized for depression?	NO....... (SKIP TO I31) .... 1<br>YES 5	NO...... (SKIP TO I31) .... 1<br>YES 5
		A. For how long?	___ ___ ___ DAYS	___ ___ ___ DAYS
(23)	I31	During this episode, were you (working/going to school) full-time?	NO .... 1<br>YES..... (SKIP TO I32) 5	NO .... 1<br>YES.... (SKIP TO I32) 5
		A. What was your major responsibility during this episode?	PART-TIME JOB .... 1<br>HOME .... 2<br>PART-TIME SCHOOL .... 3<br>OTHER: ______ .. 4	PART-TIME JOB .... 1<br>HOME .... 2<br>PART-TIME SCHOOL....... 3<br>OTHER: ______ . 4
(24)<br>DEP4C	I32	Did you have trouble functioning in this role?	NO........ (SKIP TO D) .... 1<br>YES 5	NO....... (SKIP TO D) .... 1<br>YES 5
		A. Did something happen as a result of poor functioning?	NO .... 1<br>YES ....(SPECIFY) .... 5<br>______<br>______	NO .... 1<br>YES ....(SPECIFY) .... 5<br>______<br>______
RDCE		B. Did anyone notice you had trouble functioning? (If no one was around, could someone have noticed this?)	NO .... 1<br>YES 5	NO .... 1<br>YES 5
DEP4C<br>RDCE		C. Were you completely unable to function in this role for at least 2 days in a row?	NO .... 1<br>YES 5	NO .... 1<br>YES 5
		D. Was your functioning in any other area of your life affected? (MINOR ROLE DYSFUNCTION)	NO .... 1<br>YES ....(SPECIFY) .... 5<br>______<br>______	NO .... 1<br>YES ....(SPECIFY) .... 5<br>______<br>______

(25) BOX I33 RATE FUNCTIONING:		
<u>INCAPACITATED</u> --<br>(I32C=5) COMPLETELY UNABLE TO FUNCTION IN PRINCIPAL ROLE FOR 2+ DAYS , OR<br>(I30A=2+) HOSPITALIZED 2+ DAYS , OR<br>(I29=5) ECT , OR<br>(I26 OR I26A=5) PSYCHOTIC SYMPTOMS.<br><br><u>IMPAIRED</u> --<br>(I32B=5 AND I32C=1) A DECREASE, NOTICEABLE TO OTHERS, IN QUALITY OF THE MOST IMPORTANT ROLE PERFORMANCE. THIS USUALLY REQUIRES A DECREASE IN THE AMOUNT OF PERFORMANCE.	INCAPACITATED........... 5<br>IMPAIRED......................... 4<br>NEITHER........................... 1<br><br>GO BACK TO I4 AND ASK ABOUT MOST SEVERE EPISODE.	INCAPACITATED...........5<br>IMPAIRED........................4<br>NEITHER..........................1

(32)
CLEAN

I34 Have you had at least one other severe episode when you were (depressed/uninterested in things/irritable) for at least one week that <u>did not follow</u> the death of a loved one, did <u>not</u> follow daily or (almost daily) use of alcohol or drugs, did <u>not</u> follow a serious physical illness, and did <u>not</u> follow a change in prescription medicines (**IF FEMALE:** and was <u>not</u> around the time of childbirth, miscarriage, or abortion)? **IF MORE THAN ONE ADDITIONAL CLEAN EPISODE, HAVE R PICK THE MOST SEVERE ONE.**

NO. . . (SKIP TO I35)...........1
YES....................................5

A. How old were you then?AGE: ___ ___

B. During this episode:

**COUNT ONLY IF MORE THAN USUAL:**	<u>NO</u>	<u>YES</u>
1. Were you depressed (**IF AGE IN A<18:** or irritable)?	1	5
2. Did you lose interest in pleasurable activities?	1	5
3. Did you have an increase or decrease in your appetite or weight?	1	5
4. Did you have any sleep difficulty or did you sleep too much?	1	5
5. Were you either more restless or more slowed down than usual?	1	5
6. Did you have a loss of energy or were you more tired than usual?	1	5
7. Did you feel excessively guilty or bad about yourself?	1	5
8. Did you have difficulty thinking or concentrating?	1	5
9. Did you have thoughts of dying or committing suicide, or did you make a suicide plan, or did you attempt suicide?	1	5

IF FIVE OR MORE CODED 5 IN B.1-9 (INCLUDING B.1 AND/OR B.2), CONTINUE. OTHERS SKIP TO E.

C. For how long were at least 5 of these problems present <u>nearly every day</u>, including feeling (depressed/uninterested in things/ irritable)? **IF LESS THAN 2 WEEKS, SKIP TO E.**

___ ___ WEEKS

D. When did this episode begin (when you had these experiences

___ ___ / ___ ___ ___ ___ ***t***
MO YEAR

together nearly every day)?

E. Did you have trouble managing your work, school, or household responsibilities?

NO 1
YES. . . . (SPECIFY) 5

SPECIFY:____

____

F. Did you seek help, receive any treatment (such as medications or ECT), or were you hospitalized during this episode?

NO. . . (SKIP TO I35) 1
YES. . . .(SPECIFY) 5

**SPECIFY:**	NO	YES	
1. ..... Received professional help	1	5	
2. Medications: ______________________________	1	5	CODE: __ __ __
______________________________			CODE: __ __ __
3. .... ECT (shock treatment)	1	5	
4. .... Hospitalized	1	5	

---

(33)
DIRTY

I35 Have you had at least one other severe episode when you were (depressed/uninterested in things/irritable) for at least one week that may have followed the death of a loved one, daily (or almost daily) use of alcohol or drugs, a serious physical illness, or a change in prescription medicines (**IF FEMALE:** or childbirth, miscarriage, or abortion)? **IF MORE THAN ONE ADDITIONAL DIRTY EPISODE, HAVE R PICK THE MOST SEVERE ONE.**

NO. . . (SKIP TO I36) 1
YES 5

A. How old were you then?AGE: ___ ___

B. During this episode:

**COUNT ONLY IF MORE THAN USUAL:**	NO	YES
1. Were you depressed (**IF AGE IN A<18:** or irritable)?	1	5
2. Did you lose interest in usually pleasurable activities?	1	5
3. Did you have an increase or decrease in your appetite or weight?	1	5
4. Did you have any sleep difficulty or did you sleep too much?	1	5
5. Were you either more restless or more slowed down than usual?	1	5
6. Did you have a loss of energy or were you more tired than usual?	1	5
7. Did you feel excessively guilty or bad about yourself?	1	5
8. Did you have difficulty thinking or concentrating?	1	5
9. Did you have thoughts of dying or committing suicide, or did you make a suicide plan, or did you attempt suicide?	1	5

IF FIVE OR MORE CODED 5 IN B.1-9 (INCLUDING B.1 AND/OR B.2), CONTINUE. OTHERS SKIP TO E.

C. For how long were at least 5 of these problems present nearly every day, including feeling (depressed/uninterested in things/ irritable)? **IF LESS THAN 2 WEEKS, SKIP TO E.**

___ ___ WEEKS

D. When did this episode begin (when you had these experiences

___ ___ / ___ ___ ___ ___ *t*

together nearly every day)? MO YEAR

E. Did you have trouble managing your work, school, or household responsibilities?

NO....................................... 1
YES. . . .(SPECIFY)............. 5

SPECIFY:____

____

F. Did you seek help, receive any treatment (such as medications or ECT), or were you hospitalized during this episode?

NO. . . (SKIP TO I36).......... 1
YES.......(SPECIFY) 5

SPECIFY:

	NO	YES	
1......Received professional help	1	5	
2.Medications: ______________________________	1	5	CODE: __ __ __
______________________________			CODE: __ __ __
	1	5	
3............ECT (shock treatment)	1	5	
4. ....Hospitalized			

(32F/33F) I36 How many episodes of depression lasting a week or longer (such as the one(s) we have been talking about) have you had over your lifetime, including the one(s) we already talked about?

___ ___ NUMBER

(34) A. How old were you the (first/last) time you had an episode of depression lasting a week or longer?
RECORD ALL EPISODES ON TIMELINE.

AGE ONS: ___/___ *t*
ONS: 1 2 3 4 5

AGE REC: ___/___ *t*
REC: 1 2 3 4 5

IF ANY 5 CODED IN I28, I29, I34F.2/3, OR I35F.2/3, CODE I37 "YES" SILENTLY:

(35) I37 Were you ever treated for depression with medication or ECT (shock treatment)?

NO. .(SKIP TO J1, p. 104)..... 1
YES..................................... 5

A. Did you ever feel high or were you overactive following treatment for depression with medication or ECT?

NO....................................... 1
YES..................................... 5

com.

BOX I38 IF R HAD 1+ BOX MARKED ON ALC, MJ, OR DRUG TALLY SHEET A, CONTINUE.
OTHERS SKIP TO J1, p. 104.

I38 **FOR EACH EPISODE OF DEPRESSION, ASK A.**

A. You said you had an episode of feeling (depressed/sad/down/blue/irritable) that started at (AGE).

IF 3R CLUSTERING ENDORSED ON ALC/MJ/DRUG TALLY SHEET A, HAND TALLY(IES) TO R AND ASK 1. OTHERS SKIP TO 2.

CLUSTERIN G

1. Around the time this episode of feeling (depressed/sad/

PER EPISODE

down/blue/irritable) began, were you having experiences from 3 or more boxes found on this (ALC / MJ / DRUG) sheet?

IF NO, CONTINUE TO 2.

IF YES, RECORD ON TIMELINE AND RETURN TO I38A FOR NEXT EPISODE OF DEPRESSION. IF NO OTHER EPISODES, SKIP TO I38B.

HEAVY USE PER EPISODE WHEN NOT CLUSTERING

2. Around the time this episode of feeling (depressed/sad/ down/blue/irritable) began, were you (drinking heavily/using DRUGS) daily or almost daily?

IF NO, RETURN TO I38A FOR NEXT EPISODE OF DEPRESSION. IF NO OTHERS, SKIP TO I38B.

IF YES, RECORD ON TIMELINE AND RETURN TO I38A FOR NEXT EPISODE OF DEPRESSION. IF NO OTHERS, SKIP TO I38B.

B. So, according to the information on this timeline,

CLUSTERING FOR ALL EPISODES

1. . . . your episodes of feeling (depressed/sad/down/blue/ irritable) (NEVER / SOMETIMES / ALWAYS) started around a time when you were experiencing some problems with alcohol, marijuana, or drugs?

NEVER 1
SOMETIMES...................... 3
ALWAYS (SKIP TO J1, p.104). 5

HEAVY USE FOR THE EPISODES WHEN NOT CLUSTERING

2. . . . your episodes (that did not start when you were having problems with alcohol or drugs) (NEVER / SOMETIMES / ALWAYS) started around a time when you were drinking heavily or using drugs daily (or almost daily)?

NEVER 1
SOMETIMES...................... 3
ALWAYS 5

Now I'm going to ask you some other questions about your mood.

(1) MAN3RA1 MAN4A MANICDA MANFGNA MANRDCA	K1 A.	Have you ever had a period of time lasting 2 days or longer when you felt extremely hyper, elated (unrealistically happy), or manic most of the time, <u>clearly different</u> from your normal self? **DO NOT COUNT RECOVERY FROM DEPRESSION BACK TO NORMAL MOOD.**	NO.................................... 1<br>ALC/DRUGS ONLY .... 3<br>YES 5
MAN3RA2 MAN4A MANICDA MANFGNA MANRDCA	B.	Did you ever have a period of time lasting 2 days or longer (other than when you were depressed/withdrawing from drugs) when you felt unusually irritable most of the time, clearly different from your normal self, so that you would shout at people or start fights or arguments?	NO.................................... 1<br>ALC/DRUGS ONLY .... 3<br>YES 5

BOX K1C DOES R ENDORSE MOOD? (A OR B CODED 5) **DENIES MOOD . . . . . (READ a) 1**
ENDORSES MOOD . . (READ b) 5

D. [a] Did you ever have a period of time lasting 2 days or longer, when you were <u>not</u> under the influence of alcohol or drugs, when you were...**(READ 1-7)**
AFTER THE FIRST YES, ASK: During this period were you <u>also</u>:

[b] You said you had a period of time of feeling (hyper, elated, irritable). I'm going to ask you about several other problems you might have had during this period. During this period were you also.... **(READ 1-7).**

	NO	YES
1. much more active than usual?	1	5
2. much more talkative than usual?	1	5
3. talking unusually fast or were your thoughts racing?	1	5
4. feeling very special, gifted with special powers?	1	5
5. <u>needing</u> much less sleep than usual?	1	5
6. more easily distracted than usual?	1	5
7. doing reckless or foolish things (spending sprees, reckless driving, affairs)?	1	5

DO NOT COUNT RECOVERY FROM DEPRESSION BACK TO NORMAL MOOD. CODE SX ONLY IF MORE THAN USUAL AND ONLY IF LASTED FOR 2 OR MORE DAYS.

BOX K1E IF 2 OR MORE 5'S ARE CODED IN K1D.1-7, CONTINUE TO BOX KIF. OTHERS SKIP TO K36, p.115.

BOX K1F IF R ENDORSES MOOD (BOX K1C=5), SKIP TO

(12) MAN3RA1 MAN4A MANICDA MANFGNA MANRDCA	G. You told me you experienced the following problems **(LIST SX IN K1D.1-7)**. At the time you were having these problems, were you also feeling extremely good, elated, hyper, manic, irritable, or angry, clearly different from your normal self?	NO. (SKIP TO K36, p.106) . 1<br>YES 5
(2)	K2 OMITTED	
(3)	K3 Think about your most severe episode of feeling extremely hyper, elated, or irritable that lasted 2 days or longer.	
	A. When did it begin?	___ ___ / ___ ___ ___ _<br>MO YEAR
	B. So you were ______ years old?	AGE: ___ ___
	C. How long did that episode last?	___ ___ ___ DAYS

K4 Before I ask more questions about this episode of feeling (hyper/elated/irritable), I need to know more about some other experiences you might have had at about the same time.

		**MOST SEVERE EPISODE**
MAN3RF MAN4E MANICDD MANRDCA	K5 During the 2 weeks before this episode of feeling (hyper/elated/irritable) began, how many days a week did you typically drink alcohol?	DAYS: ___<br>**IF 0 OR 1, SKIP TO K6. OTHERS CONTINUE**.
	A. On the days you drank, how many drinks would you typically have?	DRINKS: ___ ___
	**CODE SILENTLY:**<br>B. TYPICALLY 3+ (WOMAN) OR 5+ (MAN) DRINKS FOR 4+ DAYS/WEEK?	NO .... 1<br>YES. . . (SKIP TO E). 5
	C. During the 2 weeks before this episode began, what was the largest number of drinks you had in one day?	DRINKS: ___ ___<br>**IF 4 OR FEWER, SKIP TO K6.**
	D. Did you drink at least 5 drinks 2 or more times a week during the 2 weeks before this episode?	NO. . . (SKIP TO K6) ....1<br>YES 5
	E. Did you have another episode of feeling (hyper/elated/irritable) for 2 days or longer that was not after a time when you had been drinking daily or almost daily?	NO. . . (SKIP TO K11) ....1*<br>YES 5
	F. When did this episode begin?	___ ___ / ___ ___ ___ _<br>MO YEAR

1. How old were you?	AGE: ___ ___
**REMIND R WHICH EPISODE AS NEEDED**.	**MOST SEVERE EPISODE**
**IF NEVER USED MJ OR DRUGS (F1=1 AND G1=1), SKIP TO K7. OTHERS CONTINUE.**	
**HAND R CARD K.**	
MAN3RF MAN4E MANICDD MANRDCA — K6 During the 2 weeks before this episode of feeling (hyper/elated/ irritable) began, did you use any of these street drugs or abuse any prescription drugs? **IF YES:** Which ones? **CIRCLE DRUGS USED ON CARD K. CODE THE THREE USED MOST.**	NO. . . .(SKIP TO K7)......... 1<br>YES. . . . (SPECIFY) 5
DRUG 1:_______________________________________	CODE: ___ ___ ___
DRUG 2:_______________________________________	CODE: ___ ___ ___
DRUG 3:_______________________________________	CODE: ___ ___ ___
A. During the 2 weeks before this episode of feeling (hyper/elated/ irritable) began, were you taking any of the following drugs for a high or intoxication daily or almost daily? **IF YES:** Which ones? **CIRCLE DRUGS.**	NO. . . . (SKIP TO D) .... 1<br>YES. . . . (SPECIFY) ....5*<br>CIRCLE DRUG: 1 2 3
B. During that time, on average, how many days per week did you take (DRUGS)?	DRUG 1: ___ DAYS<br>DRUG 2: ___ DAYS<br>DRUG 3: ___ DAYS
C. What is the average number of times you used (DRUGS) on those days you were using?	DRUG 1: ___ ___ AVG<br>DRUG 2: ___ ___ AVG<br>DRUG 3: ___ ___ AVG
D. During the 2 weeks before this episode began, what was the largest number of times you used (DRUG) in one day?	DRUG 1: ___ ___ MAX<br>DRUG 2: ___ ___ MAX<br>DRUG 3: ___ ___ MAX
E. On how many days during that 2-week period did you use (DRUG) that much in a day?	DRUG 1: ___ ___ DAYS<br>DRUG 2: ___ ___ DAYS<br>DRUG 3: ___ ___ DAYS
	**BOX K6**<br>**IF K6A=1, SKIP TO K7. OTHERS CONTINUE.**
F. Did you have another episode of feeling (hyper/elated/ irritable) for 2 days or longer that was not after a time when you had been drinking or using drugs daily or almost daily?	NO. . . (SKIP TO K11).......1*<br>YES 5
G. When did this episode begin?	___ ___ / ___ ___ ___ ___<br>MO YEAR<br>AGE: ___ ___

1. How old were you?	
**REMIND R WHICH EPISODE AS NEEDED**.	**MOST SEVERE EPISODE**
MAN3RF MAN4E MANICDD MANRDCA K7 Did this episode of feeling (hyper/elated/irritable) begin within 2 weeks of starting or changing the dose of prescription medications such as decongestants, steroids, or antidepressants?	NO. . . .(SKIP TO K8)......... 1<br>YES. . . (SPECIFY) 5
**SPECIFY:** 1. ____	CODE: __ __ __
2.____	CODE: __ __ __
A. Did you have <u>another</u> episode of feeling (hyper/ elated/ irritable) for 2 days or longer that did <u>not</u> follow change in prescription medication, did <u>not</u> follow a serious physical illness, and was <u>not</u> after the daily or almost daily use of alcohol or drugs?	NO. . .(SKIP TO K11)........1*<br>YES 5
B. When did this episode begin?	___ ___ / ___ ___ ___ ___<br>MO YEAR
1. How old were you?	AGE: ___ ___
MAN3RF MAN4E MANICDD MANRDCA K8 Did this episode of feeling (hyper/elated/irritable) begin within the 2 weeks that followed an episode of a serious physical illness like multiple sclerosis, AIDS, hyperthyroidism, lupus, Cushings, or encephalitis?	NO. . . . (SKIP TO K9)........ 1<br>YES. . . .(SPECIFY). 5
**SPECIFY:**____ ________________________________	CODE: __ __ __
A. Did you have <u>another</u> episode of feeling (hyper/ elated/ irritable) for 2 days or longer that did <u>not</u> follow a serious physical illness and was <u>not</u> after the daily or almost daily use of alcohol or drugs?	NO. . .(SKIP TO K11)........1*<br>YES 5
B. When did this episode begin?	___ ___ / ___ ___ ___ ___<br>MO YEAR
1. How old were you?	AGE: ___ ___
MAN3RF MAN4E MANICDD MANRDCA K9 Did this episode of feeling (hyper/elated/irritable) begin shortly after receiving ECT (shock therapy) or bright light therapy?	NO. (SKIP TO BOX K11) ... 1<br>YES 5
A. Did you have <u>another</u> episode of feeling (hyper/ elated/ irritable) for 2 days or longer that did <u>not</u> follow shock or bright light therapy, did <u>not</u> follow change in prescription medication, did <u>not</u> follow a serious physical illness, and was <u>not</u> after the daily or almost daily use of alcohol or drugs?	NO. . . (SKIP TO K10) ....1*<br>YES 5
B. When did this episode begin?	___ ___ / ___ ___ ___ ___<br>MO YEAR
1. How old were you?	AGE: ___ ___

**REMIND R WHICH EPISODE AS NEEDED.**	**MOST SEVERE EPISODE**

K10 **IF NO CLEAN EPISODE EXISTS, CONTINUE THE SECTION ASKING ABOUT THE MOST SEVERE DIRTY EPISODE CODED IN K3. REMIND R WHICH EPISODE TO FOCUS ON AS FREQUENTLY AS NEEDED.**
I'd like to (return to/focus on) the most severe episode of feeling (hyper/elated/irritable) when you were ____ years old. **CHECK K3B.**

**BOX K11 A. IS EPISODE CLEAN? (DIRTY = ANY * ITEM)**	**NO, DIRTY .... 1**<br>**YES, CLEAN 5**

Now I would like to ask you about other experiences you may have had during this episode of feeling (hyper/elated/irritable).<br>During this most severe episode when you were ____years old . . .		**MOST SEVERE EPISODE**
(4)<br>MAN3RB6<br>MAN4B6<br>MANICDB1<br>MANFGNB1<br>MANRDCB1	K12 Were you much more active than usual, either socially, at work, at home, sexually, or were you physically restless?<br>**SPECIFY:**____<br>____	NO......................................1<br>YES . . . (SPECIFY) 5
(5)<br>MAN3RB3<br>MAN4B3<br>MANICDB2<br>MANFGNB2<br>MANRDCB2	K13 Were you much more talkative than usual, or did you feel pressure to keep talking?	NO......................................1<br>YES 5
(6)<br>MAN3RB4<br>MAN4B4<br>MANICDB3<br>MANFGNB3<br>MANRDCB3	K14 Did your thoughts race, or did you talk so fast that it was difficult for people to follow what you were saying (more than usual)?	NO......................................1<br>YES 5
(7)<br>MAN3RB1<br>MAN4B1<br>MANICDB6<br>MANFGNB4<br>MANRDCB4	K15 Did you feel you were a very important person, or that you had special powers, plans, talents, or abilities?<br>**SPECIFY:**____<br>____	NO......................................1<br>YES. . . . (SPECIFY) 5
(8)<br>MAN3RB2<br>MAN4B2<br>MANICDB5<br>MANFGNB5<br>MANRDCB5	K16 Did you <u>need</u> much less sleep than usual for several days in a row?<br>A. How many hours of sleep did you get per night during this episode?<br>B. How many hours do you <u>usually</u> get per night?	NO . . . (SKIP TO K17) ....1<br>YES ....5<br>___ ___ HOURS<br>___ ___ HOURS

(9)<br>MAN3RB5<br>MAN4B5<br>MANICDB7<br>MANFGNB6<br>MANRDCB6	K17 Did your attention keep jumping from one thing to another much more than is usual for you?	NO....1<br>YES....5
	During this most severe episode, when you were __ years old . . .	**MOST SEVERE EPISODE**
(10)<br>MAN3RB7<br>MAN4B7<br>MANICDB8<br>MANRDCB7	K18 Did you do anything that could have gotten you into trouble -- like spending sprees, foolish business investments, reckless driving, or sexual indiscretions?<br><br>**SPECIFY:**____________________<br>____________________	NO....1<br>YES. . . . (SPECIFY)....5
MANICDB9	A. Did your interest in sex become so much stronger than usual that you wanted to have sex a lot more frequently or with people you ordinarily would not be interested in?	NO....1<br>YES....5
MANICDB9	B. Did you talk about sexual activities, or did you approach people in a sexual manner that you ordinarily would not have? Or were you sexually indiscreet in any other way?	NO....1<br>YES....5
**BOX K19**	**COUNT THE BOXES CODED 5 IN K12-18.**<br><br>**<u>IF 0 OR 1 BOX(ES) CODED 5</u>, SKIP TO K30.**<br><br>**<u>IF 2 OR MORE BOXES CODED 5</u>, RECORD EPISODE ON TIMELINE AND CONTINUE.**	**BOXES CODED 5: _____**
	K19 You told me that while you were feeling (hyper/elated/irritable), you also experienced (**LIST SX CODED 5**). When did you start experiencing these together? (**DATE CLUSTERING OF MOOD AND SX TOGETHER**)	___ ___/___ ___ ___ ___<br>MO YEAR
	A. For how long did you experience these together?	___ ___ ___ DAYS
(13)<br>MANRDCC1	K20 During this episode, were you so excited that it was almost impossible to hold a conversation with you?<br><br>**SPECIFY:**____________________<br>____________________	NO....1<br>YES. . . . (SPECIFY)....5
MAN3RC<br>MAN4D	A. Would you say your behavior was provocative, obnoxious, or manipulative enough to cause problems for your family,	NO....1<br>YES. . . . (SPECIFY)....5

MANICDB<br>MANRDCB8	friends, or your co-workers?<br><br>**SPECIFY:**___________________________________<br><br>____	
		**MOST SEVERE EPISODE**
(14)<br>MAN3RD<br>MAN4D<br>MANICDC<br>MANFGNC	K21 During this episode did you see or hear things that other people could not see or hear, that is, did you have hallucinations?<br><br>SPECIFY:____<br><br>WHOM SAW:_____________________________________<br><br>WHAT TOLD:____________________________________	CODE: 1 2 3 4 5
MAN3RD<br>MAN4D<br>MANICDC<br>MANFGNC	A. During this episode, did you have beliefs or ideas that you later found out were not true?<br><br>SPECIFY:____________________________________<br><br>WHOM SAW:________________________________<br><br>WHAT TOLD:_______________________________	CODE: 1 2 3 4 5

BOX K21 IF ANY 5 IN K21 OR K21A, CONTINUE.
OTHERS SKIP TO K23.

	B. Did these (beliefs/ideas/hallucinations) occur before you felt (hyper/elated/irritable)?	NO........ (SKIP TO D) ....1<br>YES....................................5
MAN3RD<br>MANRDCE	C. How long before you felt (hyper/elated/irritable) did you have these (beliefs/ideas/hallucinations)?	___ ___ ___ DAYS
	D. Did these (beliefs/ideas/hallucinations) persist after your mood came back to normal?	NO. . (SKIP TO BOX K22)...1<br>YES....................................5<br>EPISODE ONGOING.........6<br><br>**IF ONGOING, SKIP TO BOX K22.**
MAN3RD<br>MANRDCE	E. How long did they last after your mood came back to normal?	__ ___ ___ DAYS

**BOX K22 DID EXAMPLES IN K21 OR K21A HAVE CONTENT CONSISTENT WITH THEMES OF INFLATED WORTH, POWER,**	**NO......................................1**<br>**YES....................................5**

KNOWLEDGE, IDENTITY, OR WITH A SPECIAL RELATIONSHIP TO A DEITY OR FAMOUS PERSON?			

		During this most severe episode when you were ____ years old . . .	MOST SEVERE EPISODE
(15)	K23	Were you seen by a doctor or other professional?<br><br>**SPECIFY:**____	NO...... (SKIP TO K27) ....1<br>YES ....(SPECIFY)............5
(16)<br>MANRDCD	K24	Did you receive medication?<br><br>**SPECIFY:**<br>1.____<br>2.____	NO......................................1<br>YES ....(SPECIFY)............5<br><br>CODE: __ __ __<br>CODE: __ __ __
(17)<br>MANRDCD	K25	Did you receive ECT (shock treatments)?	NO......................................1<br>YES 5
(18)<br>MAN4A/D<br>MANICDA	K26	Were you hospitalized during this episode for these experiences?<br><br>A. For how long?	NO...... (SKIP TO K27) ....1<br>YES ....5<br><br>___ ___ ___ DAYS
(19)	K27	During this episode, were you (working/going to school) full-time?<br><br>A. What was your major responsibility at that time?	NO......................................1<br>YES .... (SKIP TO K28) ....5<br><br>PART-TIME JOB ....1<br>HOME................................2<br>PART-TIME SCHOOL.......3<br>OTHER. . (SPECIFY) ....4<br>______________________

		During this most severe episode when you were ____ years old . . .	MOST SEVERE EPISODE
(20)	K28	Was your functioning in this role affected?<br><br>A. Did something happen as a result of this change in functioning?<br><br>SPECIFY:____<br>____	NO........ (SKIP TO D) ....1<br>YES ....5<br><br>NO......................................1<br>YES ....(SPECIFY)............5

MAN3RC<br>MAN4D<br>MANICDB<br>MANRDCC2	B. Did anyone notice that your functioning was affected? (If no one was around, could someone have noticed this?)	NO....1<br>YES....5
	.C. Were you completely unable to function in this role for at least 2 days in a row?	NO....1<br>YES....5
(21)<br>MAN3RC<br>MAN4D	D. Was your functioning in any other area of your life affected, or did you get into trouble in any way?<br><br>SPECIFY:____________________<br>____________________	NO....1<br>YES....(SPECIFY)....5

(22)	**BOX K29 RATE FUNCTIONING**<br><br>**INCAPACITATED --**<br>**(K28C=5) COMPLETELY UNABLE TO FUNCTION IN PRINCIPAL ROLE FOR 2+ DAYS , OR**<br>**(K26A=2+) HOSPITALIZED 2+ DAYS , OR**<br>**(K25=5) ECT, OR**<br>**(K21 OR K21A=5) DELUSIONS OR HALLUCINATIONS PRESENT, OR**<br>**(K20=5) INABILITY TO CARRY ON A CONVERSATION.**<br><br>**IMPAIRED --**<br>**(K28B=5 AND K28C=1) A DECREASE, NOTICEABLE TO OTHERS, IN QUALITY OF THE MOST IMPORTANT ROLE PERFORMANCE. THIS USUALLY REQUIRES A DECREASE IN THE AMOUNT OF PERFORMANCE.**<br><br>**IMPROVED --**<br>**(CHECK EXAMPLE IN K28A) IMPROVEMENT IN FUNCTION.**	**INCAPACITATED....5**<br>**IMPAIRED....4**<br>**IMPROVED....3**<br>**NEITHER....1**

(26)
CLEAN

K30 Did you have at least one other episode of 2 days or longer when you felt extremely hyper, elated, or irritable, which was <u>clearly different</u> from your normal self, when it did <u>not</u> follow daily (or almost daily) use of alcohol or drugs, did <u>not</u> follow a serious physical illness, did <u>not</u> follow a change in medicine, and did <u>not</u> follow light therapy or shock therapy?

NO(SKIP TO K31) 1
YES 5

A. How old were you then? AGE: ___ ___

B. During this episode, were you... **(READ 1-7)** **AFTER THE FIRST YES, ASK:** And at that time, were you <u>also</u>:

COUNT ONLY IF SX IS MORE THAN USUAL AND ONLY IF LASTED FOR 2 OR MORE DAYS:

	NO	YES
1. More active than usual?	1	5
2. More talkative than usual?	1	5
3. Having racing thoughts or talking too fast?	1	5
4. Feeling you were an especially important person?	1	5
5. Needing less sleep than usual?	1	5
6. Easily distracted?	1	5
7. Going on spending sprees or having sexual indiscretions?	1	5

IF 2 OR MORE ARE CODED 5, CONTINUE. OTHERS SKIP TO E.

C. When did this episode begin? ___ ___ / ___ ___ ___ ___ *t*
MO YEAR

D. How long did this episode last? ___ ___ ___ DAYS

E. Did you have trouble managing your work, school, or household responsibilities?

NO 1
YES(SPECIFY) 5

SPECIFY: ______________________________

____

F. Did you seek help, receive any treatment (such as medications or ECT), or were you hospitalized during this episode?

NO 1
YES(SPECIFY) 5

**SPECIFY:**	NO	YES	
1. Sought professional help	1	5	
2. Medications: _______________________.........	1	5	CODE: __ __ __
_________________________________			CODE: __ __ _
3. ECT (Shock treatment).......................................	1	5	
4. Hospitalized..	1	5	

(27) K31 How old were you the (first/last) time you had an episode like this?

AGE ONS:___/___ *t*
ONS:1 2 3 4 5

AGE REC:___/___ *t*
REC:1 2 3 4 5

---

(28) K32 How many episodes have you had over your lifetime, including the one(s) we have already talked about? ___ ___ ___ NUMBER

RECORD ALL EPISODES ON TIMELINE.

---

(29)
MAN4C

K33 **MIXED AFFECTIVE STATES:** During any of these episodes of feeling (hyper/elated/irritable), did you also experience:

	NO	YES
1. Depressed mood?	1	5
2. Loss of interest or pleasure?	1	5

BOX K33 IF K33.1 AND K33.2 BOTH CODED 1, SKIP TO K34. OTHERS CONTINUE.

	NO	YES
3. Sleep difficulty?	1	5
4. A change in activity level? (**PSYCHOMOTOR**)	1	5
5. Fatigue or loss of energy?	1	5
6. A change in appetite or weight?	1	5
7. Low self-esteem or guilt?	1	5
8. Decreased concentration?	1	5
9. Thoughts of dying or suicide?	1	5

IF FEWER THAN FIVE ARE CODED 5, SKIP TO K34. OTHERS CONTINUE.

A. How many episodes like this have you had (when you were both manic and depressed some of the time during the episode)? ___ ___ ___ NUMBER

---

(30) K34 Have you ever switched back and forth quickly between feeling (hyper/elated/irritable) and feeling depressed?

NO . . . .(SKIP TO BOX K35) 1
YES 5

A. Did that happen every few hours, every few days, or every few weeks?
IF MORE THAN ONE, CODE THE MOST RAPID CYCLE.

HOURS 2
DAYS 3
WEEKS 4

B. Did you ever have 4 or more episodes like this within a 12-month period?

NO 1
YES 5

BOX K35 IF R HAD 1+ BOX MARKED ON ALC, MJ,

OR DRUG TALLY SHEET A, CONTINUE.

OTHERS SKIP TO L1, p. 117.

K35 **FOR EACH EPISODE OF MANIA, ASK A.**

A. You told me about a time when you felt (unrealistically happy/elated/hyper/irritable) that started at (AGE).

IF 3R CLUSTERING ON ALC/MJ/DRUG TALLY SHEET A, HAND TALLY(IES) TO R AND ASK 1. OTHERS SKIP TO 2.

CLUSTERING PER EPISODE

1. Around the time this episode of feeling (unrealistically happy/elated/hyper/irritable) began, were you having experiences from 3 or more boxes on this (ALC / MJ / DRUG) sheet?
IF NO, CONTINUE TO 2.
IF YES, RECORD ON TIMELINE AND RETURN TO K35A FOR NEXT EPISODE. IF NO OTHER EPISODES, SKIP TO K35B.

HEAVY USE PER EPISODE WHEN NOT CLUSTERING

2. Around the time this episode of feeling (unrealistically happy/elated/hyper/irritable) began, were you (drinking heavily/using DRUGS) daily or almost daily?
IF NO, RETURN TO K35A FOR NEXT EPISODE. IF NO OTHER EPISODES, SKIP TO K35B.
IF YES, RECORD ON TIMELINE AND RETURN TO K35A FOR NEXT EPISODE. IF NO OTHER EPISODES, SKIP TO K35B.

B. So, according to the information on this timeline,

CLUSTERING FOR ALL EPISODES

1. . . . your episodes of feeling (unrealistically happy/elated/ hyper/irritable) (NEVER / SOMETIMES / ALWAYS) started around a time when you were experiencing some problems with alcohol, marijuana, or drugs?

NEVER............. 1
SOMETIMES.... 3
ALWAYS . (SKIP TO L1, p.107). 5

HEAVY USE FOR THE EPISODES WHEN NOT CLUSTERING

2. . . . your episodes (that did <u>not</u> start when you were having problems with alcohol or drugs) (NEVER / SOMETIMES / ALWAYS) started around a time when you were drinking heavily or using drugs daily (or almost daily)?

NEVER............. 1
SOMETIMES.... 3
ALWAYS.......... 5

BOX K36 SKIP TO L1, p. 117.

(31) K36 I have already asked you about episodes of extremely elated moods when you were clearly different from your

NO. . . (SKIP TO L1, p. 117) 1
ALC/DRUG ONLY 3

		normal self. Now I'd like to ask if you have ever had episodes lasting at least 2 days when you felt unusually cheerful, energetic, hyper, or irritable?<br><br>**DO NOT COUNT BRIEF EPISODES LASTING FEWER THAN 2 DAYS THAT CLEARLY FOLLOWED PERSONAL SUCCESSES, MARRIAGES, ENGAGEMENTS, OR RECOVERY FROM DEPRESSION TO NORMAL MOOD.**<br><br>**SPECIFY:**<br><br>____<br><br>**IF K36 IS CODED 5, CONTINUE.**<br>**OTHERS SKIP TO L1, p. 107.**<br><br>A. During this period were you: NO YES<br>1. much more active than usual? .... 1 5<br>2. much more talkative than usual? .... 1 5<br>3. experiencing racing thoughts? .... 1 5<br>4. feeling you were a very important person or had special powers, or talents? .... 1 5<br>5. needing less sleep than usual? .... 1 5<br>6. much more distractible than usual, when your attention kept jumping from one thing to another? 1 5<br>7. doing anything that could have gotten you into trouble, like spending sprees, or sexual indiscretions? 1 5<br>8. very friendly with people you normally would not be friendly with? .... 1 5<br><br>**IF ALL ARE CODED 1, SKIP TO L1, p. 107. OTHERS CONTINUE.**<br><br>B. How long did this period last, when these experiences occurred together with your unusually (cheerful / energetic / hyper / irritable) mood?	YES .... 5<br><br>__ __ __ DAYS
(32)	K37	How many episodes like this have you had?	___ ___ NUMBER
(33)	K38	How old were you the (first/last) time?	AGE ONS:___/___<br>ONS:1 2 3 4 5<br><br>AGE REC:___/___<br>REC:1 2 3 4 5

BEFORE CODING L1-L12, ASK FOR EXAMPLES.

Now I'm going to ask you about very unusual experiences that some people have.

(1) Auditory hallucinations, when fully awake, word heard inside or outside the head.

L1 Did you ever hear things that other people couldn't hear, such as noises, or the voices of people whispering or talking, when you were completely awake?

NO. . . . .(SKIP TO L2)............1
YES.......................................5

A. What did you hear?

EXAMPLES: ________________________________

____

B. For how long did you hear these things? __ __ __

CODE UNIT: DAYS 1
WEEKS..................2
MONTHS...............3
YEARS..................4

More than 2 words heard more than twice - with no relation to expression or elation.

C. How many times did you hear it? __ __ NUMBER

IF HEARD VOICE(S), CONTINUE.
OTHERS SKIP TO G.

D. Did it comment on what you were doing or thinking?

NO...1
YES.......................................5

E. How many voices did you hear? __ __ NUMBER

IF ONLY 1 VOICE, CODE "NO" SILENTLY.

F. Were they talking to each other?

NO...1
YES.......................................5

G. **BEGIN PROBING.**

CODE: 2 3 4 5

WHOM SAW: ________________________________

WHAT TOLD: ________________________________

(2) Visual hallucinations

L2 Did you ever see things that other people could not see or have visions when you were completely awake?

CODE: 1 2 3 4 5

DISTINGUISH FROM AN ILLUSION, I.E., A MISPERCEPTION OF A REAL EXTERNAL STIMULUS.

EXAMPLES: ________________________________

____

WHOM SAW: ________________________________

WHAT TOLD: ________________________________

BOX L2 IF NO 5'S CODED IN L1G AND L2, SKIP TO L5.

(3)<br>Tactile hallucinations	L3	What about strange sensations in your body or on your skin?<br><br>EXAMPLES: ______________________<br>______________________<br>WHOM SAW: ______________________<br>WHAT TOLD: ______________________	CODE: 1 2 3 4 5
(4)<br>Olfactory hallucinations	L4	What about smelling things that other people could not smell?<br><br>EXAMPLES: ______________________<br>______________________<br>WHOM SAW: ______________________<br>WHAT TOLD: ______________________	CODE: 1 2 3 4 5
(5)	L5	Did you ever receive special messages from the TV, radio, or newspaper, or from the way things were arranged around you?<br><br>EXAMPLES: ______________________<br>______________________<br>WHOM SAW: ______________________<br>WHAT TOLD: ______________________	CODE: 1 2 3 4 5
(8)<br>Somatic delusions: Content involves change or disturbance in body functioning.	L6	Did you ever feel that parts of your body had changed or stopped working? (What did your doctor say?)<br><br>EXAMPLES: ______________________<br>______________________<br>WHOM SAW: ______________________<br>WHAT TOLD: ______________________	CODE: 1 2 3 4 5
(10)<br>Other delusions: Guilt, jealousy, nihilism, poverty.	L7	Did you ever feel that you had committed a crime or done something terrible for which you should be punished?<br><br>EXAMPLES: ______________________<br>______________________<br>WHOM SAW: ______________________<br>WHAT TOLD: ______________________	CODE: 1 2 3 4 5
(12)	L8	Did you ever feel as if your thoughts were being	CODE: 1 2 3 4

Thought broadcasting: The delusion that one's thoughts are audible to others.	broadcast out loud so that other people could actually hear what you were thinking?<br><br>EXAMPLES: ______________________<br>______________________<br>WHOM SAW: ______________________<br>WHAT TOLD: ______________________	5
(11) Delusions of being controlled: outside force controlling own feelings, impulses, thoughts.	L9 Did you ever feel that someone or something outside yourself was controlling your thoughts or actions against your will?<br><br>EXAMPLES: ______________________<br>______________________<br>WHOM SAW: ______________________<br>WHAT TOLD: ______________________	CODE: 1 2 3 4 5
Thought insertion.	A. Did you ever feel that certain thoughts, that were not your own, were put into your head?<br><br>EXAMPLES: ______________________<br>______________________<br>WHOM SAW: ______________________<br>WHAT TOLD: ______________________	CODE: 1 2 3 4 5
Thought withdrawal.	B. What about thoughts taken out of your head?<br><br>EXAMPLES: ______________________<br>______________________<br>WHOM SAW: ______________________<br>WHAT TOLD: ______________________	CODE: 1 2 3 4 5

BOX L9 IF NO 5'S IN L5-L9, SKIP TO BOX L13. OTHERS CONTINUE.

(6) Delusions of reference: personal significance is falsely attributed to objects or events in the environment	L10 Did it ever seem that people were talking about you or taking special notice of you?<br><br>EXAMPLES: ______________________<br>______________________<br>WHOM SAW: ______________________<br>WHAT TOLD: ______________________	CODE: 1 2 3 4 5

(7) Grandiose delusions: Content involves exaggerated power, knowledge or importance.	L11 Did you ever feel that you were especially important in some way, or that you had powers to do things that other people could not do?<br><br>EXAMPLES: ________________<br>________________<br>WHOM SAW: ________________<br>WHAT TOLD: ________________	CODE: 1 2 3 4 5
(9) Persecutory delusions: individual or his/her group is being attacked, harassed, cheated, persecuted, or conspired against.	L12 Did you ever feel that people were going out of the way to give you a hard time or trying to hurt you?<br><br>EXAMPLES: ________________<br>________________<br>WHOM SAW: ________________<br>WHAT TOLD: ________________	CODE: 1 2 3 4 5

BOX L13 IF ANY 5 CODED IN L1G-L12, CONTINUE.
OTHERS SKIP TO M1, p. 123.

(13A) Systematized delusions: A single delusion with multiple elaborations or a group of delusions related to a single theme. Bizarre delusions: Involving a phenomenon that R's subculture would regard as totally implausible.	L13 What is your understanding of why you (CONTENT IN L1-L12)?<br><br>RECORD: ________________<br>________________<br>________________	EDITOR/CLINICIAN CODE:<br>Systematized delusions ....1<br>Bizarre delusions...................2<br>Other 3
(14)	L14 Did (EXPERIENCES CODED 5 IN L1-L12) last for 6 months or longer?	NO..1<br>YES......................................5
	A. Did (this experience/any of these experiences) cause you to miss work or school, or affect your ability to function at home?<br><br>**SPECIFY:** ________________<br>________________	NO..1<br>YES. . . . . (SPECIFY).............5

BOX L14 **IF L14=1 AND L14A=1, SKIP TO M1, p. 123.**
OTHERS CONTINUE.

(15) L15 How old were you the (first/last) time you had any of these experiences?

AGE ONS: ___/___ *t*
ONS: 1 2 3 4 5

AGE REC: ___/___ *t*
REC: 1 2 3 4 5

BOX L16 **CHECK I1, I2 (p. 71) AND K1A, K1B (p. 94).**
IF ANY ARE CODED 5, CONTINUE.
OTHERS SKIP TO BOX L17.

(16) L16 Were the episodes of feeling (depressed/elated/irritable) ever present at the same time you were having (beliefs/ experiences) such as (SX CODED 5 IN L1-L12)?

NO...1
YES.......................................5

(17) BOX L17	RESPONDENT'S PRESENT STATE:	NO	YES
A.	CATATONIC BEHAVIOR?	1	5
B.	FLAT AFFECT?	1	5
C.	GROSSLY INAPPROPRIATE AFFECT?	1	5
D.	INCOHERENCE?	1	5
E.	MARKED LOOSENING OF ASSOCIATION?	1	5
F.	EMOTIONAL TURMOIL?	1	5

BOX L18 **IF R HAD 1+ BOX MARKED ON ALC, MJ, OR DRUG TALLY SHEET A, CONTINUE.**
OTHERS SKIP TO M1, p. 123.

L18 **FOR EACH EPISODE, ASK A.**

A. You told me about a time when (NAME SX/your mind was playing tricks on you) when you were (AGE).

IF 3R CLUSTERING ON ALC/MJ/DRUG TALLY SHEET A, HAND TALLY(IES) TO R AND ASK 1. OTHERS SKIP TO 2.

CLUSTERING PER EPISODE

1. Around this time when (NAME SX/your mind was playing tricks on you), were you also having experiences from 3 or more boxes found on this (ALC / MJ / DRUG) sheet?

IF NO, CONTINUE TO 2.
IF YES, RECORD ON TIMELINE AND RETURN TO L18A FOR NEXT EPISODE OF PSYCHOSIS. IF NO OTHER EPISODES, SKIP TO L18B.

HEAVY USE PER EPISODE WHEN NOT CLUSTERING

2. Around this time when (NAME SX/your mind was playing tricks on you), were you (drinking heavily / using DRUGS) daily or almost daily?
IF NO, RETURN TO L18A FOR NEXT EPISODE. IF NONE, SKIP TO L18B.
IF YES, RECORD ON TIMELINE AND RETURN TO L18A FOR NEXT EPISODE. IF NONE, SKIP TO L18B.

B. So, according to the information on this timeline,

CLUSTERING FOR ALL EPISODES

1. . . . the time(s) when (NAME SX/your mind was playing tricks on you) (NEVER / SOMETIMES / ALWAYS) started around a time when you were experiencing some problems with alcohol, marijuana, or drugs?

NEVER 1
SOMETIMES 3
ALWAYS .(SKIP TO M1,p.123). 5

HEAVY USE FOR THE EPISODES WHEN NOT CLUSTERING

2. . . . the time(s) when (NAME SX/your mind was playing tricks on you) (that did <u>not</u> start when you were having problems with alcohol or drugs) (NEVER / SOMETIMES / ALWAYS) started around a time when you were drinking heavily or using drugs daily (or almost daily)?

NEVER 1
SOMETIMES 3
ALWAYS 5

**BEGIN SCORING TALLY SHEET FOR SECTION M.**<br><br>**FOR ANY AGE ONS THAT R SAYS "DK", ASK: Do you think it was before your 13th birthday or was it later than that?**<br><br>**A/D PROBE: Did this <u>ever</u> happen when you were under the influence of alcohol (or drugs)?**<br>**[IF YES:] Did this <u>only</u> happen when you were under the influence of alcohol (or drugs)?**	**UNDER 13 RECORD -1**<br>**13-14 RECORD -2**<br>**15-17 RECORD -3**<br>**18 OR OLDER RECORD -4**<br><br>**ONLY ALC/DRUGS = 3**<br>**NEVER ALC/DRUGS = 5**<br>**BOTH = 6**

Now I'd like to ask you some questions about when you were younger.

	M1	Except for your senior year in high school, did you ever play hooky from school for an entire day?	NO(SKIP TO M2) 1<br>YES 5
	A.	Did this ever happen twice in 1 year?	NO . . . . .(SKIP TO M2) 1<br>YES 5
ASP3RB1<br>CD3RA5<br>CD4A15<br>CDICD18<br>FGNASPA	B.	How old were you the first time you played hooky twice in one year? **MARK TALLY IF AGE ONSET BEFORE 13.**	AGE ONS: ___/___ A
	M2	Were you ever suspended or expelled from school?	NO(SKIP TO M3) 1<br>YES 5
FGNASPA	A.	How old were you the first time?	AGE ONS: ___/___
FGNASPB	M3	Did you ever run away from home overnight?	NO(SKIP TO M4) 1<br>YES 5
	A.	Why did you run away?<br>______________________________	**CODE SILENTLY:**<br>AVOID PHYSICAL ABUSE 2<br>AVOID SEXUAL ABUSE 3<br>OTHER 4
ASP3RB2<br>CD3RA2<br>CD4A14<br>CDICD19	B.	Did you run away overnight more than once?	NO. . . . . . (SKIP TO C) 1<br>YES 5 A
		1. How old were you the (first/last) time you ran away from home overnight?	AGE ONS: ___/___<br>AGE REC: ___/___
		**CODE AGES AND THEN SKIP TO M4.**	
ASP3RB2<br>CD3RA2	C.	After you ran away, did you return home?	NO. . . . . . (SKIP TO 2) 1 A<br>YES 5
CD4A14<br>CDICD19		1. When you ran away, how long did you stay away from home? **CHECK TALLY IF AWAY FOR 7 OR MORE DAYS.**	__ __ __ DAYS A
		2. How old were you?	AGE ONS: ___/___

	M4	Did you ever stay out late at night without permission, either for 2 or more hours after the curfew your parents set or all night without permission?	NO(SKIP TO M5) 1<br>YES 5
	A.	Did this happen 3 or more times?	NO. . . . . .(SKIP TO M5) 1<br>YES 5
CD4A13<br>CDICD12	B.	How old were you the first time?<br>**MARK TALLY IF AGE ONS LESS THAN 13.**	AGE ONS: ___/___ A
	M5	Did you ever sneak out of the house at night after your parents thought you had gone to bed?	NO. . . . . .(SKIP TO M6) 1<br>YES 5
	A.	Did this happen 3 or more times?	NO. . . . . .(SKIP TO M6) 1<br>YES 5
CD4A13<br>CDICD12	B.	How old were you the first time?<br>**MARK TALLY IF AGE ONS LESS THAN 13.**	AGE ONS: ___/___ A

(7)<br>CD3RA11<br>CD4A2	M6	Did you 3 or more times start physical fights with your brothers or sisters?	NO(SKIP TO B) 1<br>ALC/DRUGS ONLY 3<br>YES, CLEAN 5 A,B<br>BOTH A/D & CLEAN 6 A,B
ASP3RB3<br>FGNASPF	A.	At what age did you (first/last) start fights with siblings?<br>A1. UNRELATED TO ALC/DRUGS.<br>A2. IN CONTEXT OF ALC/DRUGS.<br>A3. RECENCY.	AGE ONS: ___/___<br>AGE ONS A/D: ___/___<br>AGE REC: ___/___
CD3RA11<br>CD4A2<br>CDICD10	B.	Did you 3 or more times start physical fights with persons other than your brothers and sisters?	NO(SKIP TO D)<br>ALC/DRUGS ONLY<br>YES, CLEAN 5 A,B<br>BOTH A/D & CLEAN
ASP3RB3<br>FGNASPF	C.	At what age did you (first/last) start fights with persons other than siblings?<br>C1. UNRELATED TO ALC/DRUGS.<br>C2. IN CONTEXT OF ALC/DRUGS.<br>C3. RECENCY.	AGE ONS: ___/___<br>AGE ONS A/D: ___/___<br>AGE REC: ___/___
ASP3RC3<br>ASP4A4<br>FGNASPF<br>DSICDB4	D.	(Even though you didn't start fights,) since your 15th birthday, have you been in 3 or more physical fights (other than in combat or as part of your job)?<br>**DO NOT COUNT FIGHTS WITH SIBLINGS UNLESS SOMEONE WAS HURT.**	NO . . . . .(SKIP TO M7)<br>ALC/DRUGS ONLY<br>YES, CLEAN<br>BOTH A/D & CLEAN
	E.	How old were you the (first/last) time?<br>E1. UNRELATED TO ALC/DRUGS.<br>E2. IN CONTEXT OF ALC/DRUGS.	AGE ONS: ___/___<br>AGE ONS A/D:

		E3. RECENCY.	AGE REC: ___/___
(12) CDICD3	M7	When you were younger did you <u>often</u> challenge your parents, teachers, or other adults by refusing to do things they asked you to do, just because you didn't want to? For example, refusing to do things like chores or running errands, refusing to participate in class, or not behaving well?	NO(SKIP TO M8)<br>YES
	A.	How old were you the first time?	AGE ONS: ___/___
(13) CDICD1 FGNASPF	M8	As a child, when things did not go your way, did you <u>often</u> throw temper tantrums, that is, you would throw things or lie on the ground and scream?	NO(SKIP TO M9)<br>YES 5
	A.	How old were you the first time?	AGE ONS: ___/___
(14) CD4A1 CDICD22	M9	Did people complain that you were <u>often</u> a bully, deliberately hurting, threatening, or being mean to other children?	NO(SKIP TO M10)<br>YES 5 A,B
	A.	How old were you the (first/last) time?	AGE ONS: ___/___<br>AGE REC: ___/___
(15) CD3RA8 CD4A5 CDICD14	M10	Did you ever hurt or injure a pet or any other animal on purpose?<br>**SPECIFY:** ________________________________	NO(SKIP TO M11)<br>YES . . . . . (SPECIFY)
	A.	How many times?	___ ___ TIMES
ASP3RB6	B.	How old were you the (first/last) time?	AGE ONS: ___/___<br>AGE REC: ___/___
(4) CD3RA3	M11	Throughout your life have you told <u>a lot</u> of lies?	NO 1<br>ALC/DRUGS ONLY<br>YES, CLEAN 5 A<br>BOTH A/D & CLEAN
CD3RA3 CD4A11 CDICD9	A.	Did you <u>often</u> lie to get your own way, or to get out of trouble?	NO 1<br>ALC/DRUGS ONLY<br>YES, CLEAN 5 A<br>BOTH A/D & CLEAN
	B.	Have you ever used an alias or a false name? **EXCLUDE MINORS USING FALSE ID TO BUY ALCOHOL OR ENTER A BAR.**	NO(SKIP TO BOX M11)<br>ALC/DRUGS ONLY<br>YES, CLEAN<br>BOTH A/D & CLEAN
CD3RA3 CD4A11 CDICD9		B1. Did you ever do this to take advantage of a person or a situation?	NO 1<br>YES 5 A

BOX M11 IF M11, M11A, AND M11B.1 ARE ALL CODED 1, SKIP TO M12. OTHERS CONTINUE.

ASP3RB10
ASP3RC6
ASP4A2

C. How old were you when you (first/last) (told a lot of lies / used an alias to take advantage of someone)?

C1.	UNRELATED TO ALC/DRUGS.	AGE ONS: ___/___
C2.	IN CONTEXT OF ALC/DRUGS.	AGE ONS A/D: ___/___
C3.	RECENCY.	AGE REC: ___/___

CDICD5
DSICDB6

M12 When something went wrong that was your fault, did you usually try to get out of it by blaming others?

NO(SKIP TO M13)
ALC/DRUGS ONLY 3
YES, CLEAN 5
BOTH A/D & CLEAN

A. How old were you the (first/last) time?

A1.	UNRELATED TO ALC/DRUGS.	AGE ONS: ___/___
A2.	IN CONTEXT OF ALC/DRUGS.	AGE ONS A/D: ___/___
A3.	RECENCY.	AGE REC: ___/___

CD3RA3
CD4A11
CDICD9

M13 Did you often cheat on schoolwork, on exams, in games or anything like that?

NO 1
YES 5 A

CD3RA3
CD4A11
CDICD9

A. Have you often cheated on things as an adult? Examples include cheating at work or on taxes.

NO 1
ALC/DRUGS ONLY
YES, CLEAN 5 A
BOTH A/D & CLEAN

IF M13 AND M13A ARE BOTH CODED 1, SKIP TO M14. OTHERS CONTINUE.

ASP3RB10
ASP3RC6
ASP4A2

B. How old were you the (first/last) time?

B1.	UNRELATED TO ALC/DRUGS.	AGE ONS: ___/___
B2.	IN CONTEXT OF ALC/DRUGS.	AGE ONS A/D: ___/___
B3.	RECENCY.	AGE REC: ___/___

(5)
CD3RA1
CD4A12
CDICD17
DSICDB2

M14 Did you more than once steal money or things from your family, friends, or relatives? **COUNT ONLY IF MORE THAN A FEW DOLLARS.**

NO(SKIP TO B)
ALC/DRUGS ONLY
YES, CLEAN 5 A,B
BOTH A/D & CLEAN

ASP3RB11

A. How old were you the (first/last) time?

A1.	UNRELATED TO ALC/DRUGS.	AGE ONS: ___/___
A2.	IN CONTEXT OF ALC/DRUGS.	AGE ONS A/D: ___/___
A3.	RECENCY.	AGE REC: ___/___

CD3RA1
CD4A12
CDICD17
DSICDB2

B. Did you more than once steal or shoplift from stores or from other people? (NO CONFRONTATION)

NO(SKIP TO D)
ALC/DRUGS ONLY
YES, CLEAN 5 A,B
BOTH A/D & CLEAN

ASP3RB11

	C.	How old were you the (first/last) time?<br>C1. UNRELATED TO ALC/DRUGS.<br>C2. IN CONTEXT OF ALC/DRUGS.<br>C3. RECENCY.	AGE ONS: ___/___<br>AGE ONS A/D: ___/___<br>AGE REC: ___/___
CD3RA1<br>CD4A12<br>CDICD17<br>DSICDB2	D.	Did you <u>more than once</u> forge anyone's signature on a check or credit card without permission?	NO(SKIP TO BOX M14)<br>ALC/DRUGS ONLY<br>YES, CLEAN 5 A,B<br>BOTH A/D & CLEAN
ASP3RB11	E.	How old were you the (first/last) time?<br>E1. UNRELATED TO ALC/DRUGS.<br>E2. IN CONTEXT OF ALC/DRUGS.<br>E3. RECENCY.	AGE ONS: ___/___<br>AGE ONS A/D: ___/___<br>AGE REC: ___/___
		**BOX M14 IF M14, M14B, AND M14D ARE ALL CODED 1, SKIP TO M15. OTHERS CONTINUE.**	
ASP3RC2<br>ASP4A1	F.	Since your 15th birthday, have you stolen things (or forged a signature without permission) 3 or more times?	NO 1<br>YES 5
(18)<br>CD3RA6<br>CD4A10<br>CDICD23<br>DSICDB2	M15	Did you ever break into someone else's home, car, or building (<u>not</u> because you were locked out)?	NO(SKIP TO M16)<br>ALC/DRUGS ONLY<br>YES, CLEAN 5 A,B<br>BOTH A/D & CLEAN
	A.	How old were you the (first/last) time?<br>A1. UNRELATED TO ALC/DRUGS.<br>A2. IN CONTEXT OF ALC/DRUGS.<br>A3. RECENCY.	AGE ONS: ___/___<br>AGE ONS A/D: ___/___<br>AGE REC: ___/___
ASP3RC2<br>ASP4A1	B.	Has this happened 3 or more times since you were 15?	NO 1<br>YES 5
(19)<br>CD3RA12<br>CD4A6<br>CDICD20<br>DSICDB2	M16	Have you ever taken money or property from someone else by threatening them or using force, like snatching a purse or robbing them?	NO(SKIP TO M17)<br>ALC/DRUGS ONLY<br>YES, CLEAN 5 A,B<br>BOTH A/D & CLEAN
ASP3RB12	A.	How old were you the (first/last) time?<br>A1. UNRELATED TO ALC/DRUGS.<br>A2. IN CONTEXT OF ALC/DRUGS.<br>A3. RECENCY.	AGE ONS: ___/___<br>AGE ONS A/D: ___/___<br>AGE REC: ___/___
ASP3RC2<br>ASP4A1	B.	Has this happened 3 or more times since you were 15?	NO 1<br>YES 5

(16)<br>CD3RA4<br>DSICDB2	M17	Did you ever deliberately set fires you were not supposed to?	NO(SKIP TO M18)<br>ALC/DRUGS ONLY<br>YES, CLEAN 5<br>BOTH A/D & CLEAN
CD4A8<br>CDICD16	A.	Did you do this with the intention to damage property?	NO 1<br>YES 5 A,B
	B.	How old were you the (first/last) time?	
ASP3RB9<br>ASP3RC2<br>ASP4A1<br>DSICDB2		B1. UNRELATED TO ALC/DRUGS.	AGE ONS: ___/___
		B2. IN CONTEXT OF ALC/DRUGS.	AGE ONS A/D: ___/___
		B3. RECENCY.	AGE REC: ___/___
ASP3RC2	C.	Has this happened 3 or more times since you were 15?	NO 1<br>YES 5

(6)<br>CD3RA7<br>CD4A9<br>CDICD15<br>DSICDB2	M18	Have you ever damaged someone's property on purpose (other than by fire setting)?<br>**SPECIFY:** ___________________________________<br>____	NO(SKIP TO M19)<br>ALC/DRUGS ONLY<br>YES, CLEAN 5 A,B<br>BOTH A/D & CLEAN
ASP3RB8	A.	How old were you the (first/last) time?	
		A1. UNRELATED TO ALC/DRUGS.	AGE ONS: ___/___
		A2. IN CONTEXT OF ALC/DRUGS.	AGE ONS A/D: ___/___
		A3. RECENCY.	AGE REC: ___/___
		**IF AGE ONS IS LESS THAN 15, ASK B. OTHERS SKIP TO D.**	
	B.	Did you more than once damage someone's property before you turned 15?	NO 1<br>YES 5
	C.	Since your 15th birthday, have you damaged someone else's property on purpose?	NO(SKIP TO M19)<br>YES 5
ASP3RC2<br>ASP4A1	D.	Have you done this 3 or more times since your 15th birthday?	NO 1<br>YES 5
(9)<br>CD3RA13<br>CD4A4<br>CDICD13	M19	(Outside of fighting) have you ever physically injured anyone on purpose?<br>**SPECIFY:**__________________________________<br>____	NO(SKIP TO M20)<br>ALC/DRUG ONLY<br>YES, CLEAN 5 A,B<br>BOTH A/D & CLEAN
ASP3RB7<br>ASP3RC3	A.	How old were you the (first/last) time?	

ASP4A4
FGNASPF
DSICDB4

A1. UNRELATED TO ALC/DRUGS. AGE ONS: ___/___

A2. IN CONTEXT OF ALC/DRUGS. AGE ONS A/D: ___/___

A3. RECENCY. AGE REC: ___/___

---

(8)
CD3RA10
CD4A3
CDICD11
DSICDB4
FGNASPF

M20 Did you ever use a weapon like a stick, gun, or a knife to injure someone (other than in combat or as part of your job)?

NO(SKIP TO M21)
ALC/DRUG ONLY
YES, CLEAN 5 A,B
BOTH A/D & CLEAN

ASP3RB4
ASP3RC3
ASP4A4

A. How old were you the (first/last) time?

A1. UNRELATED TO ALC/DRUGS. AGE ONS: ___/___

A2. IN CONTEXT OF ALC/DRUGS. AGE ONS A/D: ___/___

A3. RECENCY. AGE REC: ___/___

---

(26)
CD3RA9
CD4A7
CDICD21
DSICDB4

M21 Have you ever forced anyone into any sexual activity?

NO(SKIP TO BOX M22)
ALC/DRUG ONLY
YES, CLEAN 5 A,B
BOTH A/D & CLEAN

ASP3RB5
ASP3RC3
ASP4A4

A. How old were you the (first/last) time?

A1. UNRELATED TO ALC/DRUGS. AGE ONS: ___/___

A2. IN CONTEXT OF ALC/DRUGS. AGE ONS A/D: ___/___

A3. RECENCY. AGE REC: ___/___

BOX M22 IF 3 OR MORE MARKS IN PART A OF TALLY M, CONTINUE. OTHERS SKIP TO M23.

(20)
CD3R
CD4A
CDICD

M22 You mentioned that you **(LIST SX IN PART A OF TALLY M).** Did 3 or more of these ever happen within a 6-month period? **IF YES:** Which ones? **CIRCLE SX THAT CLUSTER.**

NO. . . . (SKIP TO M23)
YES 5

A. How old were you the (first/last) time?

AGE ONS: ___/___

AGE REC: ___/___
REC: 1 2 3 4 5

---

(21)

			NO	ALC/ DRUGS ONLY	YES CLEAN	BOTH A/D & CLEAN
	M23	Since your 15th birthday, have you ever....				
ASP3RC2 ASP4A1 DSICDB2	1.	Deliberately written bad checks?	1	3	5	6
ASP3RC2 ASP4A1 DSICDB2	2.	Received, sold, or bought stolen goods (fenced), sold drugs, or "run numbers" (illegally gambled)?	1	3	5	6
ASP3RC2 ASP4A1 DSICDB2	3.	Been paid for having sex with someone?	1	3	5	6
FGNASPG		a. **IF YES (3, 5, OR 6):** Were you paid with drugs?	NO 1<br>YES 5			
ASP3RC2 ASP4A1 DSICDB2 FGNASPG	4.	Found customers for male or female prostitutes or "call girls"?	1	3	5	6

IF ALL CODED 1, CONTINUE.
OTHERS SKIP TO B.

ASP3RC2
ASP4A1

A. Since your 15th birthday, have you ever done anything else that you could have been arrested for, even if you weren't (other than using drugs or underage drinking)?

NO(SKIP TO M24)	1
ALC/DRUGS ONLY	3
YES, CLEAN	5
BOTH A/D & CLEAN	6

SPECIFY:______________________________________

____

B. Did this happen 3 or more times?

NO 1
YES 5

C. How old were you the (first/last) time?

C1.	UNRELATED TO ALC/DRUGS.	AGE ONS: ___/___
C2.	IN CONTEXT OF ALC/DRUGS.	AGE ONS A/D: ___/___
C3.	RECENCY.	AGE REC: ___/___

---

(33)
ASP3RC4
ASP4A6
DSICDB2

M24 Since your 15th birthday, have you often failed to pay debts that you owed? Have you often had things you bought taken back, or often failed to take care of other financial responsibilities? (Examples: defaulting on credit card charges, loans from family or friends, car or house loans.)

NO(SKIP TO M25)	1
ALC/DRUG ONLY	3
YES, CLEAN	5
BOTH A/D & CLEAN	6

A. How old were you the (first/last) time?

A1.	UNRELATED TO ALC/DRUGS.	AGE ONS: ___/___
A2.	IN CONTEXT OF ALC/DRUGS.	AGE ONS A/D: ___/___
A3.	RECENCY.	AGE REC: ___/___

Now I have a few questions about being responsible for a child.

	M25	Before I ask, let me check, have you ever been responsible for a child for one year or longer?	NO. . . . (SKIP TO M26) 1<br>YES 5				
			NO	ALC/ DRUGS ONLY	YES CLEAN	BOTH A/D & CLEAN	
(34) ASP3RC4 ASP4A6 DSICDB2	A.	Have you <u>often</u> not provided financial support for your family when you were supposed to?	1	3	5 B	6 B	
ASP3RC8E ASP4A5 DSICDB2	B.	Have you <u>often</u> left young children under 6 at home alone while you were out shopping or doing anything else?	1	3	5 B	6 B	
ASP3RC8D ASP4A6 DSICDB2	C.	Has a neighbor fed or taken care of a child of yours because no one was taking care of the child at home?	1	3	5 B	6 B	
ASP3RC8A ASP3RC8B ASP3RC8C ASP4A6 DSICDB2	D.	Has a nurse, social worker or teacher said that your child wasn't getting enough to eat, wasn't being kept clean, or wasn't getting needed medical attention? ...	1	3	5 B	6 B	
ASP3RC8F ASP4A3 DSICDB2	E.	Have you more than once run out of money for food for your family because you had spent the food money on yourself or on going out?.........................	1	3	5 B	6 B	

IF ALL CODED 1, SKIP TO M26.
OTHERS CONTINUE.

F. How old were you the (first/last) time this happened?

F1. UNRELATED TO ALC/DRUGS. AGE ONS: ___/___

F2. IN CONTEXT TO ALC/DRUGS. AGE ONS A/D: ___/___

F3. RECENCY. AGE REC: ___/___

---

(27)
ASP3RC3
ASP4A4
DSICDB4
FGNASPE

M26 Have you ever been accused of child abuse or been the subject of a complaint on the child abuse hotline?

NO(SKIP TO M27)	1
ALC/DRUG ONLY	3
YES, CLEAN	5
BOTH A/D & CLEAN	6

A. How old were you the (first/last) time?

A1. UNRELATED TO ALC/DRUGS. AGE ONS: ___/___

A2. IN CONTEXT OF ALC/DRUGS. AGE ONS A/D: ___/___

A3. RECENCY. AGE REC: ___/___

(28)<br>ASP3RC3<br>ASP4A4<br>DSICDB4<br>FGNASPF	M27	Since you were 15, have you often hit, physically attacked, or thrown things at anyone (including your wife/husband/partner/children)?	NO(SKIP TO M28) 1<br>ALC/DRUG ONLY 3<br>YES, CLEAN 5 B<br>BOTH A/D & CLEAN 6 B
	A.	How old were you the (first/last) time?	
		A1. UNRELATED TO ALC/DRUGS.	AGE ONS: ___/___
		A2. IN CONTEXT OF ALC/DRUGS.	AGE ONS A/D:___/___
		A3. RECENCY.	AGE REC: ___/___
(10)<br>ASP3RC7<br>ASP4A5	M28	Have you ever had a traffic ticket for a moving violation (things like speeding, running a red light, or causing an accident)?	NO(SKIP TO M29) 1<br>ALC/DRUG ONLY 3<br>YES, CLEAN 5<br>BOTH A/D & CLEAN 6
FGNASPC<br>DSICDB2	A.	How many tickets have you received in your life?<br>**IF DK, ASK A1. OTHERS SKIP TO B.**	___ ___ TICKETS
FGNASPC<br>DSICDB2		A1. Was it at least 4?	NO 1<br>YES 5
	B.	How old were you the (first/last) time?	
		B1. UNRELATED TO ALC/DRUGS.	AGE ONS:___/___
		B2. IN CONTEXT OF ALC/DRUGS.	AGE ONS A/D: ___/___
		B3. RECENCY.	AGE REC: ___/___

(11)<br>ASP3RC2<br>ASP4A1<br>DSICDB2	M29	Have you ever been arrested for anything other than moving violations? **IF YES, SPECIFY. DO NOT COUNT DRUNK & DISORDERLY CONDUCT OR PUBLIC INTOXICATION.**<br>REASON(S): ______________________________	NO(SKIP TO M30) 1<br>ALC/DRUG ONLY 3<br>YES, CLEAN 5<br>BOTH A/D & CLEAN 6
	A.	How old were you the (first/last) time you were arrested?	
		A1. UNRELATED TO ALC/DRUGS.	AGE ONS:___/___
		A2. IN CONTEXT OF ALC/DRUGS.	AGE ONS A/D: ___/___
		A3. RECENCY.	AGE REC: ___/___
FGNASPC	B.	How many times have you been arrested (other than for moving violations)?	___ ___ TIMES
FGNASPC	C.	Have you ever been convicted of a felony?<br>**SPECIFY:** ______________________________	NO 1<br>YES. . . . . (SPECIFY) 5
	D.	Have you ever spent time in jail for something other than using drugs or alcohol?<br>**SPECIFY:** ______________________________	NO(SKIP TO M30) 1<br>YES. . . . . (SPECIFY) 5
DSICDB5			

	E.	Since you got out of jail have you ever been arrested for things other than using drugs or alcohol? **SPECIFY:** ______________________________	NO 1<br>YES. . . . . (SPECIFY) 5
(29)<br>ASP3RC1C<br>ASP4A3<br>DSICDB2<br>FGNASPD	M30	Since you were 15, have you quit 3 or more jobs before having another job lined up?<br>**IF 5 OR 6, SKIP TO M31. OTHERS CONTINUE.**	NO 1<br>ALC/DRUG ONLY 3<br>YES, CLEAN 5<br>BOTH A/D & CLEAN 6
ASP3RC1C<br>ASP4A3<br>DSICDB2<br>FGNASPD	A.	Since you were 15, have you dropped out of 3 or more academic programs?<br>**INCLUDE GED AND TECHNICAL TRAINING PROGRAMS.**	NO 1<br>ALC/DRUG ONLY 3<br>YES, CLEAN 5<br>BOTH A/D & CLEAN 6
(30)<br>ASP3RC1B<br>ASP4A6<br>DSICDB2	M31	On any job you have had since you were 15, have you frequently been late or absent?	NO. . . . . (SKIP TO M32) 1<br>ALC/DRUG ONLY 3<br>YES, CLEAN 5<br>BOTH A/D & CLEAN 6
	A.	What were some reasons? ____________________<br>____	
	B.	How old were you the (first/last) time?<br>B1. UNRELATED TO ALC/DRUGS.<br>B2. IN CONTEXT OF ALC/DRUGS.<br>B3. RECENCY.	AGE ONS:___/___<br>AGE ONS A/D: ___/___<br>AGE REC: ___/___
	C.	Were you reprimanded 3 or more times or ever fired because you were frequently late or absent?	NO 1<br>YES 5
(31)	M32	In the last 5 years, have you been without a job for 6 months or more?	NO(SKIP TO M33) 1<br>YES 5
ASP3RC1A<br>ASP4A6<br>DSICDB2<br>FGNASPD	A.	Was this when you were in school, laid off, sick, on strike, a full-time homemaker, retired, or in jail?	NO, ANOTHER REASON 1<br>YES. . . . (SKIP TO M33) 5
	B.	Were you having problems with alcohol or drugs at that time?	NO 1<br>YES 5
(32)<br>ASP3RC5<br>ASP4A3<br>DSICDB2<br>FGNASPH	M33	Since your 15th birthday, have you ever traveled around without any arrangements or had no regular place to live for a month or more? **DO NOT COUNT VACATIONS**.	NO(SKIP TO M34) 1<br>YES 5
	A.	How old were you the (first/last) time?	AGE ONS:___/___<br>AGE REC: ___/___
	B.	Were you having problems with alcohol or drugs at that time?	NO 1<br>YES 5

Now I'm going to ask you a few more questions about your relationships and your sexual experiences.

(22)

DSICDB3	M34	Since you were 18, have you ever had a close personal friendship or love relationship that lasted continuously for more than 1 year?	NO 1<br>YES 5<br>N/A . . . (CURRENTLY 18) 9

---

(23)	M35	How old were you when you first had sexual intercourse (voluntarily)?	AGE ONS:___/___

BOX M35 IF NEVER, CODE 00 AND SKIP TO M38.

FGNASPG	A.	How many sexual partners have you had in your life? **IF 1, SKIP TO M37. IF 2-9, SKIP TO M36. OTHERS CONTINUE.**	___ ___ ___ NUMBER
FGNASPG	B.	Have you ever had sex with 10 different people within a single year?	NO 1<br>YES 5
(24)	M36	Have you ever been unfaithful to any person in a romantic or love relationship; that is, when you had an affair or one-night stand?	NO(SKIP TO M37) 1<br>ALC/DRUG ONLY 3<br>YES, CLEAN 5<br>BOTH, A/D & CLEAN 6
(25)<br>DSICDB3<br>FGNASPE	A.	Did this happen 3 or more times?	NO 1<br>YES 5
ASP3RC9<br>DSICDB3	B.	Have you ever been faithful to 1 person for more than 1 year (that is, when you did not have any other sexual relationships)? **IF NEVER HAD A 1-YEAR RELATIONSHIP, CODE 9.**	NO, NEVER FAITHFUL 1<br>YES, WAS FAITHFUL 5<br>N/A 9
ASP3RC7<br>ASP4A5	M37	Have you more than once had unprotected sex (without a condom) with someone you believed could give you a disease, or when you had a disease that could be spread that way?	NO 1<br>ALC/DRUG ONLY 3<br>YES, CLEAN 5<br>BOTH A/D & CLEAN 6

ASP3RC7<br>ASP4A5	M38	Have you <u>often</u> taken chances where you or someone else might get physically hurt? For example, playing with fireworks or guns in a reckless manner?	NO 1<br>ALC/DRUG ONLY 3<br>YES, CLEAN 5<br>BOTH A/D & CLEAN 6

SPECIFY: ___________________________________

ASP3RC7<br>ASP4A5	A.	Have you <u>often</u> taken chances when driving--like racing a train to a crossing, or drag racing?<br><br>**SPECIFY:** _______________________________	NO 1<br>ALC/DRUG ONLY 3<br>YES, CLEAN 5<br>BOTH A/D & CLEAN 6

BOX M38 IF M38 AND M38A ARE BOTH CODED 1, SKIP TO M39. OTHERS CONTINUE.

	B.	How old were you the (first/last) time?<br>B1. UNRELATED TO ALC/DRUGS.<br>B2. IN CONTEXT OF ALC/DRUGS.<br>B3. RECENCY.	AGE ONS: ___/___<br>AGE ONS A/D: ___/___<br>AGE REC: ___/___

---

(17)	M39	Was there ever a time when you really enjoyed conning people to the point that you would <u>often</u> go out of your way to put something over on them?	NO(SKIP TO M40) 1<br>ALC/DRUG ONLY 3<br>YES, CLEAN 5<br>BOTH A/D & CLEAN 6
	A.	How old were you the (first/last) time?<br>A1. UNRELATED TO ALC/DRUGS.<br>A2. IN CONTEXT OF ALC/DRUGS.<br>A3. RECENCY.	AGE ONS: ___/___<br>AGE ONS A/D: ___/___<br>AGE REC: ___/___
ASP3RC6<br>ASP4A2	B.	Did this happen 3 or more times since your 15th birthday?	NO 1<br>YES 5

---

(35)<br>ASP3RC10<br>ASP4A7<br>DSICDB1	M40	Have you <u>often</u> ignored the feelings of others in order to do what <u>you</u> wanted?	NO 1<br>ALC/DRUG ONLY 3<br>YES, CLEAN 5<br>BOTH A/D & CLEAN 6

---

(35)<br>ASP3RC3<br>CDICD6/7<br>DSICDB6	M41	Have you <u>often</u> felt irritable, angry, or resentful (that is, you <u>frequently</u> lost your temper, or it was easy to annoy you or make you mad)?	NO 1<br>ALC/DRUG ONLY 3<br>YES, CLEAN 5<br>BOTH A/D & CLEAN 6

---

(37)<br>CDICD5<br>DSICDB6	M42	Have you <u>often</u> felt that others were to blame for your troubles or your mistakes?	NO 1<br>ALC/DRUG ONLY 3<br>YES, CLEAN 5<br>BOTH A/D & CLEAN 6

BOX M43 REVIEW PART B OF TALLY SHEET M. IF 2 OR MORE ITEMS MARKED, CONTINUE. OTHERS SKIP TO O1, p. 137.

---

(38)	M43	Now I'd like to review some of these behaviors that you told me about. You said that since the age of 15 you **(LIST SX IN PART B).** How old were you the last time you were in any of these situations?	AGE REC: ___/___<br>REC: 1 2 3 4 5
DSICDB5<br>ASP3RC10<br>ASP4A7	A.	When you were involved in any of the situations checked on this list, did you more often than not feel bad or guilty afterwards?	NO 1<br>YES . . (SKIP TO O1, p. 137) 5

DSICDB6	B.	Was that because you felt the person(s) (or animals) involved deserved it more times than not?	NO<br>YES	1<br>5

HAND R CARD O.

PTS3RA
PTS4A1

O1 Please look at this list. Have you ever experienced or witnessed something that is so horrible that it would be distressing or upsetting to almost anyone? Examples are included on this list: military combat; an assault, rape, or kidnapping; seeing someone seriously injured or killed; a flood, earthquake, large fire, or other disaster; an airplane crash or serious car accident; a shooting or bombing; or <u>any</u> situation where you feared there was a serious threat to your life or to the life of another person? **IF YES, RECORD EVENT AND CODE.**

NO. . . (SKIP TO Q1, p. 143) 1
YES.. 5

EVENT 1. ____ CODE: ___ ___
____

EVENT 2. ____ CODE: ___ ___
____

EVENT 3. ____ CODE: ___ ___
____

IF ONLY ONE EVENT, SKIP TO B.
OTHERS CONTINUE.

A. Which event was the most disturbing to you? **CIRCLE EVENT NUMBER AND REFER TO THIS EVENT THROUGHOUT SECTION.**

EVENT: 1 2 3

PTS4A2

B. When this most disturbing event occurred, did you feel intense fear, helplessness, or horror?

NO 1
YES.. 5

C. When did this (EVENT) occur?

___ ___ / ___ ___ ___ ___
MO YEAR

1. How old were you then?

AGE: ___ ___

D. Was there ever a period of time lasting one month or longer when you had strong feelings or thoughts about (EVENT), which made you anxious or upset?

NO . . (SKIP TO Q1, p. 143) ...1
YES 5

1. When did this start?

___ ___ / ___ ___ ___ ___ *t*
MO YEAR

AGE: ___ ___

2. So, that was when you were ___ years old?

I am going to ask you some questions about that period when you were (AGE IN O1D.2), when you were having the most, or most intense, feelings or experiences about (EVENT) . . .

PTS3RB1
PTS4B1

O2 Did memories, visions, thoughts, or feelings about (EVENT) often keep coming to your mind, even though you didn't want them to?

NO 1
YES . . . . . (SPECIFY) 5

IF YES: Can you give me some examples?

____

____

PTS3RB2
PTS4B2

O3 Did you have unpleasant dreams again and again about (EVENT)?

NO 1
YES 5

Still focusing on the period that started (DATE IN O1D.1). . . (that is, the period of a month or longer when you were having the most, or most intense, feelings or experiences about (EVENT))

PTS3RB3
PTS4B3

O4 Did you ever suddenly act or feel as if (EVENT) was happening again? This may include flashbacks or hallucinations, even if they occur when you are just waking up.

NO 1
YES . . . . . (SPECIFY) 5

IF YES: Can you give me some examples?

____

____

PTS3RB4

O5 Did you feel very upset when you were reminded of

NO 1

PTS4B4		(EVENT)? For example, on the anniversary of (EVENT).	YES 5
PTS3RD6<br>PTS4B5	O6	Did things that reminded you of (EVENT) make you sweat, tense up, breathe hard, tremble, or respond in some other physical way?	NO ....1<br>YES 5

BOX O7 IF O2-O6 ALL CODED 1, SKIP TO Q1, p. 143.
OTHERS CONTINUE.

During that period of a month or longer when you were having the most, or most intense, feelings or experiences about (EVENT), **(REMIND R OF DATE IN O1D.1) . . .**

PTS3RC1<br>PTS4C1	O8	Did you ever try to avoid thinking or having feelings about (EVENT) and find that you couldn't?	NO ....1<br>YES 5
PTS3RC2<br>PTS4C2	O9	Did you avoid activities, places, or people that reminded you of (EVENT)?<br><br>**IF YES:** Can you give me some examples?<br><br>____________________________________<br><br>____________________________________	NO ....1<br>YES . . . . . (SPECIFY) 5
PTS3RC3<br>PTS4C3	O10	Did you find that you sometimes could <u>not</u> remember important things about (EVENT)?	NO ....1<br>YES 5
PTS3RC4<br>PTS4C4	O11	During that period of time, did you lose interest in some things or stop doing some things that had been important to you before (EVENT) happened?	NO ....1<br>YES 5
PTS3RC5<br>PTS4C5	O12	During that period of time, did you feel more cut off,	NO 1

		distant, or separated from people than before (EVENT) happened?	YES . . . . . (SPECIFY) 5
		**IF YES:** Can you give me some examples? ____________________ ____________________	
PTS3RC6<br>PTS4C6	O13	Were there times when you believed you had lost your ability to experience emotions that you had before (EVENT) happened? For example, did you feel you couldn't have loving feelings or anything like that?	NO ....1<br>YES 5
PTS3RC7<br>PTS4C7	O14	Were there times when you felt that there was no point in planning for the future--that you might not have a rewarding career; a happy family; or a long, good life?	NO ....1<br>YES 5

BOX O15 REVIEW O8-O14. IF 3 OR MORE CODED 5, CONTINUE. OTHERS, SKIP TO Q1, p. 143.

During that period of a month or longer when you were having the most, or most intense, feelings or experiences about (EVENT), **(REMIND R OF DATE IN O1D.1)** . . .

PTS3RD1<br>PTS4D1	O16	Did you have more trouble falling asleep or staying asleep than before (EVENT)?	NO ....1<br>YES 5
PTS3RD2<br>PTS4D2	O17	Did you find that you got irritated or lost your temper more easily than before (EVENT)?	NO ....1<br>YES 5
PTS3RD3<br>PTS4D3	O18	Were there times when you had more trouble concentrating than before (EVENT)?	NO ....1<br>YES 5

PTS3RD5<br>PTS4D5	O19	Were there times when unexpected noise, movement, or touch startled you more than before (EVENT)?	NO ....1<br>YES 5
PTS3RD4<br>PTS4D4	O20	Were you more watchful or extremely aware of things around you? For example, were you more aware of certain sounds, smells, or sights?	NO ....1<br>YES 5

BOX O21 REVIEW O16-O20. IF 2 OR MORE CODED 5, CONTINUE. OTHERS, SKIP TO Q1, p. 143.

PTS3RE<br>PTS4E	O22	You have told me about things such as reliving the event through dreams, memories, or feelings; avoiding things that reminded you of the event; and problems with sleep, mood, or thinking. Did these experiences last longer than one month?	NO . (SKIP TO Q1, p.143).......1<br>ALC/DRUG ONLY................ 3<br>YES, CLEAN......................... 5<br>BOTH A/D & CLEAN........... 6
	A.	What is the longest amount of time that these experiences lasted?	MONTHS: ___ ___ ___
	B.	How soon after (EVENT) did you begin to experience these things?	___ ___ ___ UNITS *t*<br>**CODE UNITS:**<br>DAYS ....1<br>WEEKS ....2<br>MONTHS ....3<br>YEARS 4
	C.	How old were you the last time you had a period of time like this?	AGE REC: ___/___ *t*<br>REC: 1 2 3 4 5
PTS4F	D.	Did these experiences interfere with your work, school, household activities, or how you got along with other people?<br>**SPECIFY:** ______________________________	NO ....1<br>YES. . . . (SPECIFY)..............5
	O23	Did you ever talk to a doctor or other professional about the problems you had after the (EVENT)?	NO 1

YES. . . . (SPECIFY) 5

SPECIFY: ________________________________

BOX O24 IF R HAD 1+ BOX MARKED ON ALC, MJ, OR DRUG TALLY SHEET A, CONTINUE.
OTHERS SKIP TO Q1, p. 143.

O24 We talked about the time when you had very intense feelings after you experienced (EVENT). I recorded that this troubling period of time started at (AGE).

IF 3R CLUSTERING ON ALC/MJ/DRUG TALLY SHEET A, HAND TALLY(IES) TO R AND ASK A. OTHERS SKIP TO B.

CLUSTERING AT ONSET

A. Around the time you first had these very intense feelings, were you having experiences from 3 or more boxes found on this (ALC / MJ / DRUG) sheet?

NO 1
YES . . . . (SKIP TO Q1, p. 143) 5

HEAVY USE WHEN NOT CLUSTERING

B. Around the time you first had these very intense feelings, were you (drinking heavily/using DRUGS) daily or almost daily?

NO 1
YES... 5

(1) OCD3RA1 OCD4A1/2	Q1	Have you ever had thoughts, images, or impulses that bothered you a lot and kept coming back? Ideas that are senseless -- like thinking your hands are dirty no matter how often you wash them or thinking of hurting someone you love when you're not even mad at them. Other examples are the repeated urge to curse in church or feeling sure many times that you have run over someone with your car.<br><br>Please describe these to me:<br><br>EXAMPLES: ______________________________<br>______________________________<br>______________________________	NO. . . (SKIP TO Q9) .... 1<br>YES 5
	A.	**CODE SILENTLY:** ARE EXAMPLES IN Q1 ONLY ABOUT OWN EMOTIONAL PROBLEMS, ALC/ DRUG PROBLEMS, HEALTH/APPEARANCE, OR REALISTIC FINANCIAL/FAMILY PROBLEMS?	NO. . . .(SKIP TO D) .... 1<br>YES 5
OCD4D/E	B.	Were the kinds of thoughts, images, or impulses that bothered you only about (your emotional problems / problems you had with alcohol or drugs / other problems you had with your health or appearance / realistic money or family problems) ?	NO... 1<br>YES . . . (SKIP TO Q9)................ 5
	C.	What other kinds of thoughts or ideas bothered you?<br><br>EXAMPLE: ______________________________<br>______________________________<br>______________________________	
	D.	**BEGIN PROBING**<br><br>WHOM SAW: ______________________________<br>WHAT TOLD: ______________________________	CODE: 2 3 4 5<br><br>**IF CODED 2, SKIP TO Q9. OTHERS CONTINUE.**
OCD3RA2 OCD4A3	Q2	Did you try to block these thoughts by doing something or thinking about something else?	NO. . . .(SKIP TO Q9) .... 1<br>YES 5
OCD3RA3 OCD4A4	Q3	Were these your own thoughts or were they put in your head by someone else?<br><br>**IF CODED 1, SKIP TO Q9. OTHERS CONTINUE.**	SOMEONE ELSE...................... 1<br>OWN THOUGHTS.................... 5
OCD4B	Q4	Did you think that these (thoughts/images/impulses) were unreasonable or excessive?	NO. . . .(SKIP TO Q9) .... 1<br>YES 5

BOX Q5 IF I25A=5 OR I25C=5, CONTINUE.
OTHERS SKIP TO Q6.

OCD3RA4
OCD4D

Q5 Did these thoughts <u>only</u> occur when you were feeling sad, blue, or depressed, like the times we talked about earlier?

NO 1
YES 5

OCD3RA4
OCD4E

Q6 Did these thoughts <u>only</u> occur when you were using alcohol or drugs or had recently cut down?

NO 1
YES 5

(4)
OCD3RB
OCD4C

Q7 Did these thoughts really upset you or interfere with your normal routine?

NO 1
YES. . . .(SPECIFY) 5

SPECIFY: ____

____

(5)
OCD3RB
OCD4C

A. Did you find yourself having these thoughts or impulses for at least an hour a day?

NO 1
YES 5

BOX Q7 IF Q7 AND Q7A ARE BOTH CODED 1,
SKIP TO Q9.

Q8 When was the (first/last) time you experienced these thoughts to the point that they interfered with your normal routine or caused you to feel really upset?

AGE ONS: ___/___ *t*
ONS: 1 2 3 4 5

AGE REC: ___/___ *t*
REC: 1 2 3 4 5

(3)
OCD3RA1
OCD4A1

Q9 Have you ever found that you <u>had</u> to do or think certain things over and over? For example, washing your hands so often your skin became sore or checking things like doors many times because you thought you hadn't locked them? What about performing behaviors in a set pattern? For example, putting on your clothes in a certain order, counting repeatedly, saying words to yourself over and over, or other rituals like that?

NO. (SKIP TO R1, p.147)............ 1
YES. . . (SPECIFY) 5

SPECIFY: ____

____

OCD3RA2
OCD4A2

A. Did you do those things to keep something bad from happening?

NO 1
YES. . . .(SPECIFY) 5

SPECIFY: ____

____

B. **BEGIN PROBING.**

CODE: 2 3 4 5

WHOM SAW: ______________________________

WHAT TOLD: ______________________________

IF CODED 2, SKIP TO R1, p.147. OTHERS CONTINUE.

OCD3RA2
OCD4A2

Q10 If you tried to stop doing (BEHAVIOR), did you become anxious or very nervous?

NO 1
YES. . . . (SPECIFY) 5

SPECIFY: ______________________________

____

(Q3C)
OCD3RA3
OCD4B

Q11 Did you think that these activities were unreasonable or excessive?

NO 1
YES 5

BOX Q12 IF BOX H5B = 5 OR H11 = 5, CONTINUE. OTHERS SKIP TO BOX Q13.

OCD4D

Q12 Were these activities always related to feelings about your body size or weight?

NO 1
YES. . . .(SPECIFY) 5

SPECIFY: ______________________________

____

BOX Q13 IF I25A = 5 OR I25C = 5, CONTINUE. OTHERS SKIP TO Q14.

OCD4D

Q13 Did you perform these behaviors only when you were feeling sad, blue, or depressed, like the times we talked about earlier?

NO 1
YES 5

OCD4E

Q14 Did these behaviors only occur when you were using alcohol or drugs or had recently cut down?

NO 1
YES 5

(Q4)
OCD3RB
OCD4C

Q15 Did those activities really upset you or interfere with your normal routine?

NO 1
YES. . . .(SPECIFY) 5

SPECIFY: ______________________________

____

(Q5)
OCD3RB
OCD4C

A. Did you find yourself performing these behaviors at least an hour at a time each day?

NO 1
YES 5

BOX Q15 IF Q15 AND Q15A ARE BOTH CODED 1, SKIP TO R1, p.147. OTHERS CONTINUE.

Q16	When was the (first/last) time you performed these activities to the point that they caused you to feel really upset, interfered with your normal routine, or took up a lot of your time?	AGE ONS: ___/___ *t*<br>ONS: 1 2 3 4 5<br>AGE REC: ___/___ *t*<br>REC: 1 2 3 4 5

BOX Q17 IF R HAD 1+ BOX MARKED ON ALC, MJ, OR DRUG TALLY SHEET A, CONTINUE.
OTHERS SKIP TO R1, p. 147.

Q17 You told me about the (thoughts/behaviors) that occurred over and over, which first started at (AGE).

IF 3R CLUSTERING ON ALC/MJ/DRUG TALLY SHEET A, HAND TALLY(IES) TO R AND ASK A OTHERS SKIP TO B.

CLUSTERING AT ONSET	A.	Around the time you first had repeated (thoughts/ behaviors), were you having experiences from 3 or more boxes found on this (ALC / MJ / DRUG) sheet?	NO .... 1<br>YES . . . (SKIP TO R1, p.147) 5
HEAVY USE WHEN NOT CLUSTERIN G	B.	Around the time you first had repeated (thoughts/ behaviors), were you (drinking heavily/using DRUGS) daily or almost daily?	NO .... 1<br>YES 5

(P7)
SP3RA
SP4A

R1 Some people have a strong and persistent fear of doing certain things in front of people like speaking, eating, or writing because they think they might embarrass themselves. These fears are stronger than the feelings that most people have.

Have you ever had a strong and persistent fear of:		NO	YES
1.	starting or keeping up conversations or talking to people you don't know well?	1	5
2.	speaking to your teachers, boss or other people in authority?	1	5
3.	speaking in public or answering questions in a meeting or a class?	1	5
4.	eating or drinking in public?	1	5
5.	writing while someone watches?	1	5
6.	using public restrooms? (inability to perform, not fear of germs)	1	5

IF R1.1-6 ARE ALL NO, CODE 1 AND SKIP TO S1, p. 150. OTHERS, BEGIN PROBING.

SPECIFY: ________________________________

____

WHOM SAW: ________________________________

WHAT TOLD: ________________________________

R1A. EDITOR'S CODE:
CAN SX BE EXPLAINED BY OTHER DISORDER?
NO.......................................1

CODE: 1 2 3 4 5

SP3RB
SP4G/H

IF PHYSICAL DISABILITY/CONDITION MADE THE ACT DIFFICULT, CODE 4.
IF R FEARED REVEALING A PSYCHIATRIC DISORDER OR IF SX WERE DUE TO A PSYCHIATRIC DISORDER, CODE 5.

IF CODED 1 OR 2, SKIP TO S1, p. 150.

(P8)
SP3RC
SP4B

R2 Did being in (this/these) situation(s) almost always make you extremely nervous right away (when you were not using alcohol or drugs)?

NO . . (SKIP TO S1, p. 150) 1
YES 5

(P10)
SP3RD
SP4D

A. Did you almost always avoid that situation?

NO 1
YES. . . . (SKIP TO R3) 5

(P10A)

B. When you had to be in that situation, did you almost

NO . . (SKIP TO S1, p. 150) 1

SP4D always feel extremely nervous or panicky? YES 5

(P12)
SP3RF
SP4C

R3 Did you ever think that your fear was excessive or unreasonable?
NO . . (SKIP TO S1, p. 150) 1
YES 5

(P11)
SP3RE
SP4E

R4 Did this fear or avoiding the situation ever interfere with your job, school, social functioning, or normal routine?
NO 1
YES. . . . (SPECIFY) 5

SPECIFY: ______________________________________

____

SP3RE
SP4E

A. Have you been <u>very</u> upset with yourself for having any of these fears?
NO 1
YES 5

BOX R5 IF R4 AND R4A ARE BOTH CODED 1, SKIP TO S1, p. 145. OTHERS CONTINUE.

R6 About how long did your fear (interfere with your functioning/make you upset with yourself)?
MONTHS: ___ ___ ___

R7 How old were you the (first/last) time (this fear/any of these fears) (interfered with your functioning/made you upset with yourself)?

AGE ONS: ___/___ *t*
ONS: 1 2 3 4 5

AGE REC: ___/___ *t*
REC: 1 2 3 4 5

R8 Did you ever take medicine, begin to drink or use drugs, or increase the amount of alcohol or drugs that you were using because of (this fear/these fears)?
NO. . . (SKIP TO BOX R9) 1
YES. . . . (SPECIFY) 5

SPECIFY:
1. ______________________________________ CODE: ___ ___ ___
2. ______________________________________ CODE: ___ ___ ___

A. Did (taking medicine/drinking alcohol/using drugs) help?
NO 1
YES 5

BOX R9 IF R1.4 = 5 AND EITHER H5B OR H11 = 5, CONTINUE. OTHERS SKIP TO S1, p. 150.

(P14)
SP3RB
SP4H

R10 Did any of these fears occur because you were afraid people would notice you had an eating problem?
NO 1
YES 5

BOX R11 IF R HAD 1+ BOX MARKED ON ALC, MJ, OR DRUG TALLY SHEET A, CONTINUE. OTHERS SKIP TO S1, p. 150.

R11 You told me about feeling very concerned about (SITUATIONS) in public and that first started at (AGE).

IF 3R CLUSTERING ON ALC/MJ/DRUG TALLY SHEET A, HAND TALLY(IES) TO R AND ASK 1. OTHERS SKIP TO 2.

CLUSTERING AT ONSET

A. Around the time you first felt concerned about (SITUATIONS), were you having experiences from 3 or more boxes found on this (ALC / MJ / DRUG) sheet?

NO 1
YES . . (SKIP TO S1, p. 150) 5

HEAVY USE WHEN NOT CLUSTERING

B. Around the time you first felt concerned about (SITUATIONS), were you (drinking heavily / using DRUGS) daily or almost daily?

NO 1
YES 5

AGP3RA<br>AGP4A	S1	Some people have a fear of being in certain places or situations where they feel it would be difficult to leave easily. They are worried that they could not escape or get help if they suddenly became panicky. Some situations like this include being alone away from home; being in a crowd; being in a place where there was a long distance between exits, like in a tunnel or on a bridge; travelling in a bus, car, or train; or being in an elevator. Have you ever had a period of time when you had a fear like that (that you might become panicky and wouldn't be able to leave easily if that happened)?	NO(SKIP TO T1, p.153) 1<br>YES 5

AGP3RA — S2 Did you feel this way about:

		NO	YES
1.	going outside of the house alone?	1	5
2.	being in a crowd or standing in a line?	1	5
3.	being on a bridge or in a tunnel?	1	5
4.	travelling in a bus, train, or car?	1	5
5.	being in an elevator?	1	5

IF ALL ARE CODED 1, CONTINUE.
OTHERS SKIP TO B.

A. What situation did you have in mind when you said some situations made you unreasonably afraid?

NONE. . (SKIP TO T1, p. 148) 1

ANY.............. 5

EXAMPLE:______________________________________

AGP4A — B. Did more than one situation make you feel this way?

NO 1
YES 5

S2D. EDITOR'S CODE:
CODE: 2 3 4 5

AGP4C — C. **BEGIN PROBING. SPECIFY FEAR AND RECORD EXAMPLES.**

What was it about (SITUATIONS) that was frightening to you?

IF CODED 2, SKIP TO T1, p. 153. OTHERS CONTINUE.

EXAMPLES:_____________________________________

____

____

____

WHOM SAW:____________________________________

WHAT TOLD:___________________________________

AGP3RA<br>AGP4B	S3	A. When you were in those situations, did you <u>usually</u>:		NO	YES
		1.	get sweaty?	1	5
		2.	tremble?	1	5
		3.	have a dry mouth?	1	5
		4.	feel dizzy?	1	5
		5.	feel your heart pound?	1	5
		6.	get nauseated or vomit?	1	5
		7.	feel like you couldn't control your bodily functions?	1	5
		8.	feel tightness or pain in your chest or stomach?	1	5
		9.	feel that you, or things around you, seemed unreal?	1	5

AGP4A

B. When you were in situations like (SITUATIONS IN S2), were you afraid that any of these things might happen?

NO 1
YES................ 5

AGP3RA
AGP4B

S4 Did you almost always avoid these situation(s) or stop going places because of your fear that you would feel sick or do something embarrassing?

NO 1
YES(SPECIFY)5

SPECIFY:____

____

AGP3RA
AGP4B

A. Has your fear kept you from going somewhere you wanted to go 3 or more times?

NO 1
YES(SPECIFY)5

SPECIFY: _____________________________________

____

AGP3RA
AGP4B

B. When you had to be in one of these situations, did it almost always make you extremely nervous or panicky?

NO 1
YES(SPECIFY)5

SPECIFY:_____________________________________

____

AGP3RA
AGP4B

C. When you had to be in one of these situations, did you begin to need someone to be with you?

NO 1
YES(SPECIFY)5

SPECIFY:_____________________________________

____

BOX S4 IF S4, S4A, S4B, AND S4C ARE ALL CODED 1, SKIP TO T1, p. 153. OTHERS CONTINUE.

	S5	How old were you the (first/last) time you had this fear and had some other problems like (SX ENDORSED IN S3 AND S4) at the same time?	AGE ONS: ___/___ *t*<br>ONS:1 2 3 4 5<br>AGE REC: ___/___ *t*<br>REC:1 2 3 4 5
	S6	Did you ever take medicine, begin to drink or use drugs, or increase the amount of alcohol or drugs that you were using because of this fear?	NO(SKIP TO BOX S7) 1<br>YES(SPECIFY) 5

SPECIFY:

1. ____ CODE: ___ ___ ___
2. ____ CODE: ___ ___ ___

	A.	Did (taking medicine/drinking alcohol/using drugs) help?	NO .... 1<br>YES................ 5

BOX S7 IF R HAD 1+ BOX MARKED ON ALC, MJ, OR DRUG TALLY SHEET A, CONTINUE. OTHERS SKIP TO T1, p. 153.

S7 You told me you had a concern about being in a situation where you could not escape if something bad would happen to you. I recorded that this started for you at (AGE).

IF 3R CLUSTERING ON ALC/MJ/DRUG TALLY SHEET A, HAND TALLY(IES) TO R AND ASK A. OTHERS SKIP TO B.

CLUSTERING AT ONSET	A.	Around the time you first started feeling concerned about not being able to escape if needed, were you having experiences from 3 or more boxes found on this (ALC / MJ / DRUG) sheet?	NO .... 1<br>YES . . . . (SKIP TO T1, p. 153) 5
HEAVY USE WHEN NOT CLUSTERING	B.	Around the time you first started feeling concerned about not being able to escape if needed, were you (drinking heavily/ using DRUGS) daily or almost daily?	NO .... 1<br>YES................ 5

(O1)
PAN3RA/E
PAN4A
AGPAN3RA
AGPAN4A1

T1 Have you ever had a spell or attack when all of a sudden you felt frightened, anxious, or panicky in situations when most people would not be afraid or anxious; that is, during times when you were not in danger, or were not making a speech, or something like that?

CODE: 1 2 3 4 5
IF CODED 1 OR 2, SKIP TO W1, p. 157 . OTHERS CONTINUE.

EXAMPLE: ______________________________
____
____

WHOM SAW: ______________________________

WHAT TOLD: ______________________________

(O2)
PAN3RB
AGPAN3RA

T2 Have you ever had...

		NO	YES
A.	3 attacks within a three-week period?	1	5
B.	4 attacks within a four-week period?..................	1	5

(O3)
PAN3RB
PAN4A2A
AGPAN3RA
AGPAN4A2A

T3 After having an attack, did you ever have a month or more when you worried a lot about having an attack or you were afraid that you might have another attack?

NO... 1
YES... 5

A. Did you think that having attacks like this must mean that you had a serious illness or that you were going crazy?

NO . . . . . .(SKIP TO B) 1
YES... 5

1. Did you think that for a month or longer?

PAN4A2B
AGPAN4A2B

NO... 1
YES... 5

B. Did having an attack like this cause you to stop doing anything that you used to do or stop going places you used to go?

NO. . . . . . (SKIP TO C) 1
YES... 5

1. Did you stop doing things or going places for a month or longer?

PAN4A2C
AGPAN4A2C

NO... 1
YES... 5

C. After having an attack like this, did you begin to need someone to go with you?

NO. . . . . . (SKIP TO T4)............. 1
YES... 5

1. Did that last for a month or longer?

PAN4A2C
AGPAN4A2C

NO... 1
YES... 5

(O4)
PAN3RC
PAN4A1
AGPAN3RA
AGPAN4A1

T4 During <u>one</u> of your worst attacks, did you have...

		NO	YES
PAN3RC1 PAN4A1.4	1. Shortness of breath or feeling that you were smothering?	1	5
PAN3RC3 PAN4A1.1	2. Palpitations or a pounding heart?	1	5
PAN3RC2 PAN4A1.8	3. Dizziness, light-headedness, unsteadiness, or feeling faint?	1	5
PAN3RC11 PAN4A1.6	4. Chest tightness or chest pain?	1	5
PAN3RC9 PAN4A1.12	5. Numbness or tingling in your face, feet, or fingers?	1	5
PAN3RC6 PAN4A1.5	6. Choking sensation?	1	5
PAN3RC5 PAN4A1.2	7. Sweating?	1	5
PAN3RC4 PAN4A1.3	8. Shaking or trembling?	1	5
PAN3RC10 PAN4A1.13	9. Flushing, hot flashes, or chills?	1	5
PAN3RC8 PAN4A1.9	10. A feeling that things were unreal?	1	5
PAN3RC12 PAN4A1.11	11. A fear that you might die?	1	5
PAN3RC13 PAN4A1.10	12. A fear that you were going crazy or losing control?	1	5
PAN3RC7 PAN4A1.7	13. Nausea or discomfort in your stomach or abdomen?	1	5

BOX T4 IF 4 OR MORE ARE CODED 5 IN T4.1-13, CONTINUE. OTHERS SKIP TO W1, p. 157.

(O5)
PAN3RD
PAN4A1
AGPAN3RA
AGPAN4A1

T5 You mentioned you had attacks of feeling frightened and some problems like (SX IN T4.1-13). How many episodes have you had in your lifetime that had 4 or more of these problems?

___ ___ NUMBER

BOX T5 IF ONLY 1 ATTACK, SKIP TO W1, p. 157. OTHERS CONTINUE.

(O6)
PAN3RD
PAN4A1
AGPAN3RA
AGPAN4A1

T6 During at least several of your attacks, did some of these problems such as: (UP TO 4 SX CODED IN T4) begin suddenly, and get worse in the first 10 minutes of the attacks?

NO.. 1
YES.. 5

PAN4D

PAN4D	T7	A. **IF ANY 5 CODED IN R1.1-6 (SOCPHOB, p. 142), ASK**: Did you have attacks like that when you were (SOCPHOB SITUATIONS CODED 5 IN R1.1-6)?	NO .... 1<br>YES 5
		B. **IF ANY 5 CODED IN S2.1-5 (AGPHOB, p. 145), ASK**: Did you have attacks like that when you were (AGPHOB SITUATIONS CODED 5 IN S2.1-5)?	NO .... 1<br>YES 5
		C. Did being in any (other) particular situations make it likely that you would have an attack like this?<br><br>**SPECIFY**: ______________________________<br>______________________________	NO . . . . . (SKIP TO D) .... 1<br>YES . . . . . (SPECIFY) 5
		D. Have you had these attacks at times when you had no reason to expect one because you were not in any special situation?	NO .... 1<br>YES 5
(O7)	T8	How old were you the (first/last) time you had one of these sudden attacks of feeling frightened or anxious when you had 4 or more problems like (ALL SX CODED 5 IN T4.1-13)?<br><br>**IF DK AND R IS UNDER 40, CODE T8A "YES" WITHOUT ASKING. IF DK AND R IS 40 OR OLDER, ASK A. OTHERS SKIP TO T9.**	AGE ONS: ___/___ ***t***<br>ONS: 1 2 3 4 5<br>AGE REC: ___/___ ***t***<br>REC: 1 2 3 4 5
		A. **IF DK:** Would you say that the first time was before you were 40?	NO .... 1<br>YES 5
(O8)	T9	Have you ever been nervous or anxious much of the time between attacks?	NO .... 1<br>YES 5
(O9)	T10	Did these attacks ever cause you to have difficulty in getting along with your family or to have problems at work or at school?<br>**SPECIFY:** ______________________________	NO .... 1<br>YES . . . . . (SPECIFY) 5
	T11	Did you ever take medicine, begin to drink or use drugs, or increase the amount of the alcohol or drugs that you were using because of these attacks?<br><br>**SPECIFY:**<br>1. ______________________________<br>2. ______________________________	NO . . . (SKIP TO BOX T12) .... 1<br>YES . . . . . (SPECIFY) .... 5<br><br>CODE: ___ ___ ___<br>CODE: ___ ___ ___

A. Did (drinking/using drugs) help?

NO 1
YES 5

BOX T12 IF R HAD 1+ BOX MARKED ON ALC, MJ, OR DRUG TALLY SHEET A, CONTINUE. OTHERS SKIP TO W1, p. 157.

T12 We talked about sudden attacks of feeling panicky, frightened, or nervous. You said that first happened at (AGE).

IF 3R CLUSTERING ON ALC/MJ/DRUG TALLY SHEET A, HAND TALLY(IES) TO R AND ASK A. OTHERS SKIP TO B.

CLUSTERING AT ONSET

A. Around the time the attacks first started, were you having experiences from 3 or more boxes found on this (ALC / MJ / DRUG) sheet?

NO 1
YES . . (SKIP TO W1, p. 157) 5

HEAVY USE WHEN NOT CLUSTERING

B. Around the time the attacks first started, were you (drinking heavily/ using DRUGS) daily or almost daily?

NO 1
YES 5

W: SUBJECT COMMENTS

As you can see, I tried to ask you about a lot of different kinds of emotional problems, physical and medical problems, and habits that people might have. But, of course, everyone is different, and I might have skipped something that has been important to you. Have you had any problems I should have covered but didn't?

RECORD VERBATIM:_______________________________________

Do you have any comments about the interview itself?

RECORD VERBATIM:_______________________________________

RECORD TIME ENDED: ___ ___:___ ___

(USE 24 HOUR CLOCK)

BORDERLINE	= 3
DEFINITE	=4
DOES NOT APPLY	= 9

TYPE OF INTERVIEW: (Choose 1)

...PERSONAL INTERVIEW...................... 1
TELEPHONE INTERVIEW................2
PROXY INTERVIEW.............................3

IF CODED 5 OR 9, SKIP TO NEXT QUESTION.

A. FACIAL EXPRESSION IS NORMAL? NO.........1
YES 5
PHONE..9

1. Sad..3 4 9
2. Gloomy 3 4 9
3. Hostile 3 4 9
4. Worried 3 4 9
5. Avoids gaze 3 4 9
6. Immobile 3 4 9

B. DRESS IS NORMAL? NO.........1
YES..5
PHONE.....................................9

1. Meticulous..3 4 9
2. Clothing, hygiene poor...............................3 4 9
3. Eccentric... ....3 4 9
4. Seductive 3 4 9
5. Inadequate for warmth and protection 3 4 9

C. MOTOR ACTIVITY IS NORMAL? NO.........1
YES...5
PHONE...9

1. Increased amount...................................3 4 9
2. Constantly fiddling, changing position, standing or sitting down.......................... . 3 4 9
3. Agitation..3 4 9
4. Tics...3 4 9
5. Tremor..3 4 9
6. Peculiar posturing 3 4 9
7. Unusual gait.. ..3 4 9
8. Repetitive acts..................................... ...3 4 9
9. Very slow to move; unusual for age & physical condition..................... . 3 4 9
10. Rigid posture 3 4 9

D. FLOW OF THOUGHT IS NORMAL? NO.......1
YES..5

1. Blocking...3 4 9
2. Circumstantial 3 4 9
3. Tangential 3 4 9
4. Perseveration 3 4 9
5. Flight of ideas 3 4 9
6. Indecisive 3 4 9
7. Illogica..3 4 9

E. LEVEL OF CONSCIOUSNESS IS NORMAL? NO.......1 YES......5

1. Hypervigilant 3 4 9
2. Drowsy 3 4 9
3. Stupor.. 3 4 9

F. SPEECH IS NORMAL? NO.......1
YES......5

1. Excessive amount 3 4 9
2. Reduced amount 3 4 9
3. Push of speech 3 4 9
4. Slowed... 3 4 9
5. Loud.. 3 4 9
6. Soft... ... 3 4 9
7. Mute.. 3 4 9
8. Slurred... 3 4 9
9. Stuttering.................................... .. 3 4 9
10. Neologisms................................... 3 4 9
11. Gloomy, voice choking on distressing topic......................... .. 3 4 9
12. Fails to answer, questions need repeating 3 4 9
13. Monotonous voice..........................3 4 9

G. INTERVIEW BEHAVIOR IS NORMAL? NO.......1 YES......5

1. Angry outbursts 3 4 9
2. Irritable... . 3 4 9
3. Impulsive..................................... 3 4 9
4. Hostile.. . 3 4 9
5. Silly.. 3 4 9
6. Sensitive 3 4 9
7. Apathetic...................................... 3 4 9
8. Withdrawn..................................... 3 4 9
9. Evasive. 3 4 9
10. Passive... 3 4 9
11. Aggressive.................................... 3 4 9
12. Naive...3 4 9
13. Overly dramatic...............................3 4 9
14. Manipulative...................................3 4 9
15. Dependent......................................3 4 9
16. Uncooperative.................................3 4 9
17. Demanding.....................................3 4 9
18. Negativistic.....................................3 4 9
19. Callous...3 4 9

H. MOOD AND AFFECT ARE NORMAL? NO......1

YES......5

1. Anxious 3 4 9
2. Inappropriate affect 3 4 9
3. Flat affect 3 4 9
4. Elated mood 3 4 9
5. Depressed mood 3 4 9
6. Labile mood 3 4 9

I. CONTENT OF THOUGHT IS NORMAL? NO......1
YES......5

1. Suicidal thoughts..................................3 4 9
2. Suicidal plans................................ ......3 4 9
3. Assaultive ideas 3 4 9
4. Homicidal thoughts.................................3 4 9
5. Homicidal plans 3 4 9
6. Antisocial attitudes.................................3 4 9
7. Suspiciousness................................ ....3 4 9
8. Poverty of content...................................3 4 9
9. Phobias 3 4 9
10. Obsessions..3 4 9
11. Compulsions.... ....3 4 9
12. Feelings of unreality 3 4 9
13. Feels persecuted 3 4 9
14. Thoughts of running away 3 4 9
15. Somatic complaints..................................3 4 9
16. Ideas of guilt 3 4 9
17. Ideas of hopelessness... ....3 4 9
18. Ideas of worthlessness 3 4 9
19. Excessive religiosity................................3 4 9
20. Sexual preoccupation.......................... .3 4 9
21. Blames others 3 4 9
22. Illusions are present...............................3 4 9
23. Auditory hallucination............................ 3 4 9
24. Visual hallucination 3 4 9
25. Other hallucinations 3 4 9
26. Delusion of persecution..........................3 4 9
27. Delusion of grandeur..............................3 4 9
28. Delusion of reference..............................3 4 9
29. Delusion of influence..............................3 4 9
30. Somatic delusion 3 4 9
31. Other delusions.................................... 3 4 9
32. Delusions are systematized.....................3 4 9

J. ORIENTATION IS NORMAL? NO.......1

YES.....5

1. Time 3 4 9
2. Place................................. .... 3 4 9
3. Person............................... .... 3 4 9

K. MEMORY IS NORMAL? NO.......1
YES......5

1. Clouding of consciousness.............. 3 4 9
2. Inability to concentrate.... ....3 4 9
3. Amnesia 3 4 9
4. Poor recent memory 3 4 9
5. Poor remote memory...................... 3 4 9
6. Confabulation..... ....3 4 9

L. INTELLECT IS NORMAL? NO.......1

YES.......5

1. Above normal 3 4 9
2. Below normal.......................... ..... 3 4 9
3. Paucity of knowledge.... ....3 4 9
4. Vocabulary poor 3 4 9

M. INSIGHT AND JUDGEMENT ARE NORMAL? NO.......1
YES......5

1. Poor insight...................................3 4 9
2. Poor judgement 3 4 9
3. Unrealistic regarding degree of illness..3 4 9
4. Doesn't know why being treated..........3 4 9
5. Unmotivated for treatment.................3 4 9

RATE ACCURACY OF CODES THROUGHOUT SSAGA-II:

NO DIFFICULTY 1
SOME PROBLEMS, BUT MOST RATINGS REASONABLY ACCURATE 2
MAJOR DIFFICULTY IN CONDUCTING EXAM 3
IMPOSSIBLE TO RATE WITH ANY CONFIDENCE..4

CODE: __ __ **Global Assessment of Functioning Scale (GAF Scale)**

Consider psychological, social, and occupational functioning on a hypothetical continuum of mental health-illness. Do not include impairment in functioning due to physical (or environmental) limitations.

Code

90–81 Absent or minimal symptoms (e.g., mild anxiety before an exam), good functioning in all areas, interested and involved in a wide range of activities, socially effective, generally satisfied with life, no more than everyday problems or concerns (e.g., an occasional argument with family members).

80–71 If symptoms are present, they are transient and expectable reactions to psychosocial stressors (e.g., difficulty concentrating after family argument); no more than slight impairment in social, occupational, or school functioning (e.g., temporarily falling behind in school work).

70–61 Some mild symptoms (e.g., depressed mood and mild insomnia) OR some difficulty in social, occupational, or school functioning (e.g., occasional truancy, or theft within the household), but generally functioning pretty well, has some meaningful interpersonal relationships.

60–51 Moderate symptoms (e.g., flat affect and circumstantial speech, occasional panic attacks) OR moderate difficulty in social, occupational, or school functioning (e.g., few friends, conflicts with co-workers).

50–41 Serious symptoms (e.g., suicidal ideation, severe obsessional rituals, frequent shoplifting) OR any serious impairment in social, occupational, or school functioning (e.g., no friends, unable to keep a job).

40–31 Some impairment in reality testing or communication (e.g., speech is at times illogical, obscure, or irrelevant) OR major impairment in several areas, such as work or school, family relations, judgment, thinking, or mood (e.g., depressed man avoids friends, neglects family, and is unable to work; child frequently beats up younger children, is defiant at home, and is failing at school).

30–21 Behavior is considerably influenced by delusions or hallucinations OR serious impairment in communication or judgment (e.g., sometimes incoherent, acts grossly inappropriately, suicidal preoccupation) OR inability to function in almost all areas (e.g., stays in bed all day; no job, home, or friends).

20–11 Some danger of hurting self or others (e.g., suicide attempts without clear expectation of death, frequently violent, manic excitement) OR occasionally fails to maintain minimal personal hygiene (e.g., smears feces) OR gross impairment in communication (e.g., largely incoherent or mute).

10–1 Persistent danger of severely hurting self or others (e.g., recurrent violence) OR persistent inability to maintain minimal personal hygiene OR serious suicidal act with clear expectation of death.
